# Pediatric cancer risk in association with birth defects: A systematic review

**DOI:** 10.1371/journal.pone.0181246

**Published:** 2017-07-27

**Authors:** Kimberly J. Johnson, Jong Min Lee, Kazi Ahsan, Hannah Padda, Qianxi Feng, Sonia Partap, Susan A. Fowler, Todd E. Druley

**Affiliations:** 1 Brown School, Washington University in St. Louis, St. Louis, Missouri, United States of America; 2 Department of Pediatrics, Washington University School of Medicine, Washington University in St. Louis, St. Louis, Missouri, United States of America; 3 Department of Neurology, Stanford University, Palo Alto, California, United States of America; 4 Division of Pediatric Hematology and Oncology, Washington University School of Medicine, Washington University in St. Louis, St. Louis, Missouri, United States of America; National Health Research Institutes, TAIWAN

## Abstract

**Background:**

Many epidemiological studies have examined associations between birth defects (BDs) and pediatric malignancy over the past several decades. Our objective was to conduct a systematic literature review of studies reporting on this association.

**Methods:**

We used librarian-designed searches of the PubMed Medline and Embase databases to identify primary research articles on pediatric neoplasms and BDs. English language articles from PubMed and Embase up to 10/12/2015, and in PubMed up to 5/12/2017 following an updated search, were eligible for inclusion if they reported primary epidemiological research results on associations between BDs and pediatric malignancies. Two reviewers coded each article based on the title and abstract to identify eligible articles that were abstracted using a structured form. Additional articles were identified through reference lists and other sources. Results were synthesized for pediatric cancers overall and for nine major pediatric cancer subtypes.

**Results:**

A total of 14,778 article citations were identified, of which 80 met inclusion criteria. Pediatric cancer risk was increased in most studies in association with BDs overall with some notable specific findings, including increased risks for CNS tumors in association with CNS abnormalities and positive associations between rib anomalies and several pediatric cancer types.

**Conclusions:**

Some children born with BDs may be at increased risk for specific pediatric malignancy types. This work provides a foundation for future investigations that are needed to clarify specific BD types predisposing toward malignancy and possible underlying causes of both BDs and malignancy.

## Introduction

Pediatric cancer is diagnosed in >14,000 U.S. children per year from the ages of 0–19 years and is the leading cause of disease-related death among children aged 1–14 years [1, 2]. Although a few risk factors have been conclusively identified, including exposure to high dose radiation and certain genetic syndromes, the etiology underlying most cases remains unknown.

Evidence accumulated over several decades suggests positive associations between birth defects (BDs) and pediatric malignancy. BDs affect ~1 in 33 U.S. children and are defined as “structural changes present at birth that can affect almost any part or parts of the body” [3]. BDs are often categorized as major and minor with major anomalies generally considered those having “an adverse effect on the individual’s health, functioning or social acceptability” and minor anomalies considered those having “limited social or medical significance”. Major anomaly examples include spina bifida, cleft lip palate, and Down Syndrome [4]. Minor anomaly examples include low set ears, epicanthal folds, and simian crease [5]. Although certain genetic syndromes are known to increase pediatric cancer risk (e.g. Down Syndrome and leukemia), other BDs (including major and minor), independent of known cancer predisposition syndromes, may also be associated with an increased risk.

Recent evidence suggests that 8.5% of children with cancer harbor germline mutations in well-known cancer genes that predispose them to the development of early-onset malignancy [6]. However, when considering a more broad predisposition definition including family history, co-morbidities, and types of pediatric cancer, up to about a third of children with cancer may have a genetic predisposition [7]. Many pediatric cancer predisposition syndromes involve defects in normal development and many pediatric cancers arise from immature cell types (e.g. the “blastomas”). Thus, identifying and understanding connections between abnormal fetal or childhood development and the risk of developing cancer will have implications for surveillance, prognosis, risk stratification and potential personalized therapeutics.

To summarize evidence on associations between pediatric malignancy and BDs, we conducted a systematic literature review to identify articles reporting primary human epidemiological research. This work provides a foundation for future studies and may help to identify high risk populations for malignancy among individuals with BDs that will enable improved surveillance, mechanistic research, targeted treatment and outcomes.

## Methods

Abbreviations used in this review are provided in Table 1. Our review followed the 2009 Preferred Reporting Method for Systematic Reviews (PRISMA) guidelines [8] (See S1 Checklist). A summary of the review protocol is provided below.

**Table 1:** Abbreviations.

Abbreviation	Full name	Abbreviation	Full name
ALL	Acute Lymphocytic Leukemia	MDS	Myelodysplastic Syndrome
AML	Acute Myeloid Leukemia	MPD	Myeloproliferative Disease
ANLL	Acute Non-Lymphoblastic Leukemia	NB	Neuroblastoma
AST	Astrocytoma	NHL	Non-Hodgkin's Lymphoma
BC	British Columbia	NOS	Newcastle-Ottawa scale
BDs	Birth Defects	NA	Not applicable
CBT	Childhood brain tumors	ND	Not determined
CI	Confidence Interval	NR	Not Reported
CNS	Central Nervous System	NRCT	National Registry of Childhood Tumours
CPP	Collaborative Perinatal Project	NWTS	National Wilms’ Tumor Study
DS	Down syndrome	OR	Odds Ratio
EPD	Ependymoma	OS	Osteosarcoma
ES	Ewing's Sarcoma	PNET	Primitive neuro-ectodermal tumor
GCT	Germ cell tumors	PRISMA	Preferred Reporting Method for Systematic reviews
HB	Hepatoblastoma	RB	Retinoblastoma
HD	Hodgkin’s Disease	RB	Retinoblastoma
HGG	High grade glioma	RDD	Random Digit Dialing
HL	Hodgkin's Lymphoma	RMS	Rhabdomyosarcoma
HM	Hematologic malignancy	RMS	Rhabdomyosarcoma
HR	Hazard Ratio	RR	Risk Ratio
IARC	International Agency For Research On Cancer	SNS	Sympathetic Nervous System
ICD	The International Classification of Diseases	STS	Soft Tissue Sarcomas
IRR	Incidence Rate Ratio	WAGR syndrome	Wilms tumor-aniridia intellectual disability syndrome
LGG	Low Grade Glioma	WT	Wilm’s tumor
LHT	Lymphatic and hematopoietic tissues	UPDB	Utah Population database
MB	Medulloblastoma		

### Search strategy

We identified relevant articles in the Medline PubMed [9] and Embase [10] databases on 9/10/2015 and 10/12/2015 using librarian designed search strategies. The review was updated on 5/12/2017 using the PubMed database. (S1 Table). We also examined each article’s reference list from those included in the review and consulted a well-known review published by the International Agency for Cancer Research in 1999 for any additional studies [11]. Finally, the senior author conducted a few PubMed literature searches based on her expert opinion to identify additional qualifying articles.

### Eligibility criteria and review process

The citation lists obtained from each database search were evaluated for initial eligibility by at least two coauthors (KA, JL, and KJ). We excluded review articles, editorial commentaries, meeting abstracts, articles focused solely on syndromes with known genetic etiologies, on treatment or clinical outcomes, solely on adults (≥ 18 years), and articles not providing information on risks specific to children with the exception of the Gelberg et al. study that reported risks for osteosarcoma from 0–24 years [12]. In addition, lab-based studies and case reports/series without external comparison groups were excluded. Reviewers classified each article initially based on title review (and abstract if eligibility was unclear from the title) as: 1) eligible, 2) unclear eligibility, and 3) ineligible. Following reconciliation of articles with coding disagreement by the senior author and reviewers, articles assigned to category one were abstracted.

### Data collection

Data were abstracted from each article using a pre-designed form that captured: study population, study design and description, sources of information on BDs and cancer, study inclusion and exclusion criteria, age groups studied, birth years, cancer diagnosis years, BD case definition, BD types examined, cancer types examined, subject numbers, analysis method, measure of association reported, and key findings. Any additional information relevant to interpretation of study results was captured in a comment field. To the extent possible, we abstracted the number of subjects for each comparison group and included this information where the numbers were reported directly or required minimum assumptions to calculate. Any articles that were identified as ineligible during the abstraction phase were subsequently excluded. During our update of the review, we also abstracted information where reported on classification of major and minor anomalies, maximum age at which BDs were ascertained, and cancer risks by age. We did not attempt to reclassify anomalies from the original reports for summary purposes.

### Quality assessment

We employed a modified Newcastle-Ottawa Scale (NOS), designed for quality assessment of observational studies [13], that uses a star assignment system to determine overall quality for case-control, nested case-control, case-cohort, and cohort studies. Case series studies with external comparison groups were not evaluated. Evaluation criteria are described briefly below.

#### Case-control, nested case-control, and case-cohort studies

**A) Selection.** Up to four stars were awarded to studies based on: 1) adequate case definition, 2) representativeness of cases, 3) control selection, and 4) adequate control definition. A case definition was considered adequate if cases were defined based on either clinical and/or histopathological validation of their cancer diagnosis or were identified through cancer registry data. Cancer cases were considered representative if they were community/population-based versus hospital-based. For control selection, a star was given if controls were selected from the same population as cases. The control definition was considered adequate if the authors provided information to indicate that controls did not have a history of the outcome.

**B) Comparability between the groups.** Up to two stars were awarded to studies matching on or adjusting for maternal age and child’s sex, potential confounders of the association between pediatric malignancy and BDs.

**C) Ascertainment of the exposure.** Up to three stars were awarded to studies if: 1) BDs were ascertained through medical records, physical exam, or birth certificate data, 2) they used the same method of BD ascertainment for cases and controls, and 3) they reported a similar non-response rate for both cases and controls (<10% difference) or the study used registry data for BD ascertainment.

#### Cohort studies

**A) Selection.** Up to four stars were awarded to studies based on: 1) representativeness of the exposed cohort, 2) selection of the non-exposed cohort, 3) ascertainment of exposure, and 4) demonstration that the outcome of interest was not present at the start of the study. Studies were given one star each if the exposed cohort registry/population included all children from a defined geographic region was representative of the community, if the non-exposed cohort was drawn from the same community as the exposed cohort, if medical records or BD registry information was used to identify exposed subjects or if the exposure was based on birth certificate data, and if it was indicated that cancer was not present at the start of the study.

**B) Comparability.** Comparability criteria were the same as for case-control studies.

**C) Outcome.** Up to two stars were awarded based on: 1) assessment of the outcome and 2) follow-up length. One star was awarded if the outcome was assessed independently or through medical records or record linkage (i.e. through administrative data through ICD codes). One star was given if the follow-up period was long enough for outcomes to occur (we set this at ≥ 6 years with consideration for the age distribution of pediatric cancer).

### Statistical methods

Unadjusted odds ratios (ORs) and 95% confidence intervals (CIs) were calculated for case-control studies where authors provided data for calculations but did not include these measures of association. To summarize the individual quality of each study, we computed the total quality points by summing the number of stars received. To compare overall quality of cohort studies vs. other study designs that differed in their maximum achievable quality points (8 for cohort vs. 9 for case-control, nested case-control, and case-cohort studies), we calculated a mean total percent quality for each study design category by dividing the mean total quality points by the total quality points possible.

## Results

**Fig 1:**
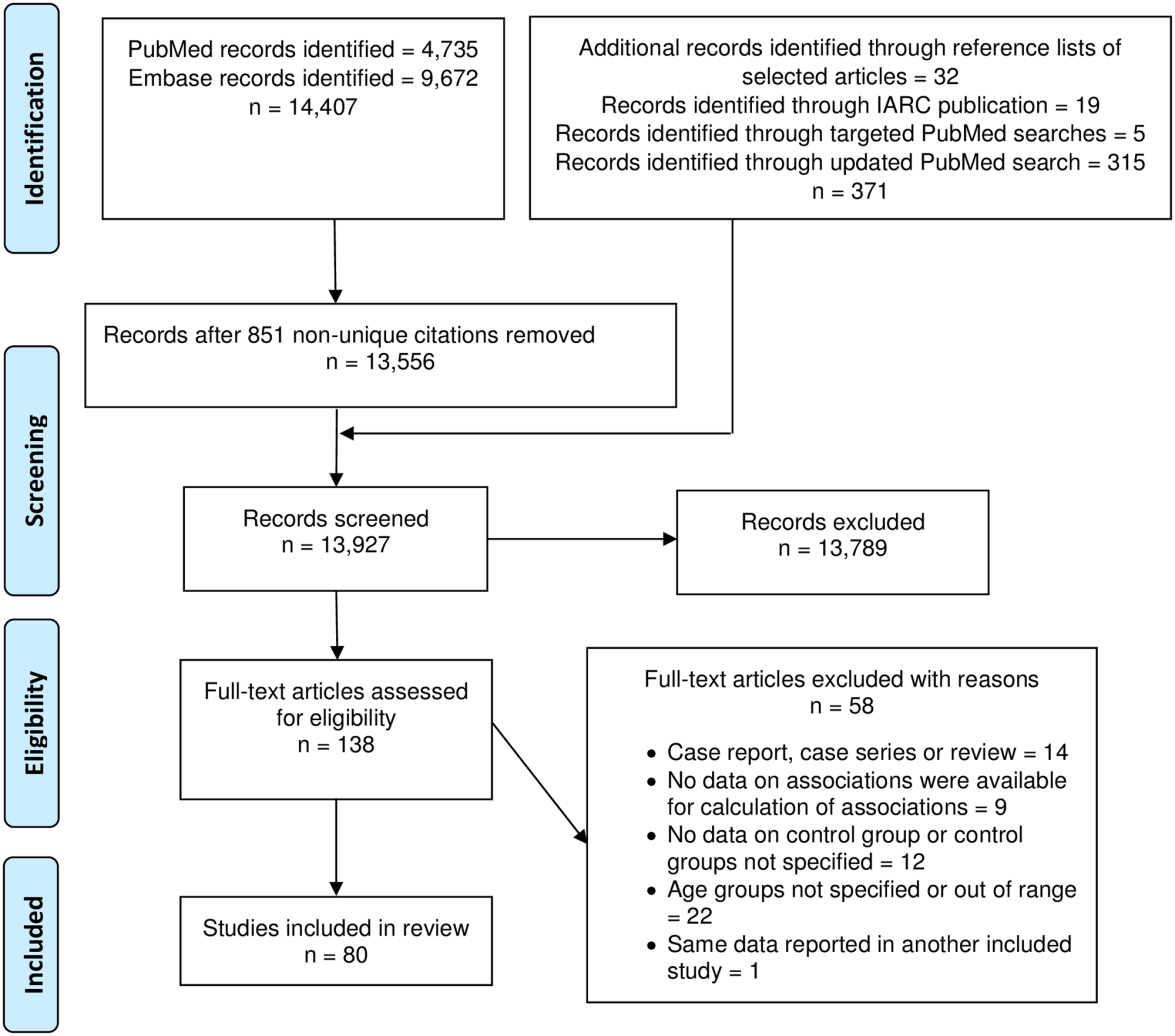
PRISMA flow diagram. The number of records screened is equal to the sum of the number of records initially identified in PubMed and Embase after removing overalapping citations and the number of studies with citations identified through other sources shown in the upper most right text box. After exclusions of non-relevant articles during the screening phase, 138 full-text text articles were abstracted, 58 of which were excluded leaving a total of 80 articles that were included in the review.

A total of 14,407 article citations, 9,672 from Embase and 4,735 from PubMed, were initially identified. After removing 851 non-unique citations (844 in both PubMed and Embase and 7 contained in Embase twice), 13,556 citations remained. With the addition of 371 articles identified through the reference lists of selected articles, the IARC publication [11], additional PubMed searches, and the updated systematic search in PubMed, 13,927 records were screened for eligibility of which 13,789 were excluded. One hundred thirty-eight full-text articles were abstracted and 80 were included (Fig 1).

We organized our review with summaries of the characteristics of included studies followed by their results for pediatric cancer types using the International Classification of Childhood Cancer third edition major diagnostic categories [14]. Within each major cancer type, results are summarized by study design with overall associations generally reported first followed by findings for specific abnormalities/subgroups.

### Characteristics of studies included (Table 2)

**Table 2:** Characteristics of included studies on the association between birth defects and childhood cancer.

Reference (location)	BD ascertainment	Pediatric cancer case ascertainment: cancer types, age group, diagnosis dates/years, case identification source	Comparison group ascertainment
***Case-control studies***			
Stewart et al., 1958 [18] (England and Wales)	Maternal interview	Overall, 0–9 years, 1953–1955 (died of cancer), Registrar General	Controls were selected from birth registers matched to cases on age, sex, and locality.
Ager et al., 1965 [39] (Minnesota, U.S.)	Maternal interview/medical record verification. The authors note that “lesser conditions, such as nevi, were not recorded”.	Leukemia, 0–4 years, 1953–1957 (died of leukemia), Minnesota death certificates	Two control groups were included with a total of two controls per case. If available, sex-matched sibling controls were included with the birthdate closest to the index child and who had reached an age at the time of interview that was the same or older as the age of the index child when they died. Neighborhood controls were matched on sex and birth date within one year.
Swerdlow et al., 1982 [91] (United Kingdom)	The Oxford Survey of Childhood Cancer and antenatal clinical notes	Testicular tumors, 0–15 years, 1953–73 (died of testicular cancer), the Oxford Survey of Childhood Cancer	The comparison group was "all boys who died during the same period from malignant neoplasms other than of genital site or of teratoma histology".
Wilkins et al., 1984 [77] (Ohio, U.S.)	Ohio birth certificate files	WT, NR, 1/1/1950-10/31/1981, Columbus (Ohio) Children's Hospital Tumor Registry	Two controls per case were randomly selected from birth certificate files with one matched to the case on sex, race, and birth year and the other matched on these variables plus mother's county of residence.
Johnson et al., 1985 [66] (Texas, U.S.)	The Texas State Department of Health birth certificates. Both major and minor BDs were included.	NB, 0–14 years, 1964–1978 (died of NB), Texas death certificates	Two controls per case were randomly selected from birth certificates matched to cases on birth year.
Méhes et al., 1985 [19] (Switzerland)	Pediatric examination for minor BDs	Overall, leukemia, solid tumors, 1.6–22 years, NR, Swiss University Children's Hospitals of Basel and Zurich Oncologic Departments	Controls with infectious diseases were matched 1:1 to cases on sex, age, and ethnic origin. Siblings were also examined where available.
Bunin et al., 1987 [78] (Philadelphia, U.S.)	Parental interview	WT, 0–14 years, 1970–1983, three Philadelphia-area hospitals: Children's Hospital of Philadelphia, St. Christopher's Hospital for Children, and Wilmington Medical Center	Controls were selected by RDD matched to cases at a 1:1 ratio on telephone area code and exchange, birth year (within 3 years), and race.
Johnson et al., 1987 [62] (Texas, U.S.)	The Texas State Department of Health birth certificates	CNS, 0–14, deaths from 1964–1980, Texas Department of Health death certificates	Two controls per case were randomly selected from Texas live births frequency matched to cases on race, sex, and birth year.
Neglia et al., 1988 [67] (Minnesota, U.S.)	The Minnesota State Department of Health birth certificates. The authors note that BDs were coded by HICDA-9.	NB, 0–8.9 years, cases born since 1969, hospital review of NB cases seen in Minnesota and bordering states	Four controls per case were selected from live births in Minnesota matched to cases on birth year.
Shu et al., 1988 [48] (Shanghai, China)	Interview of parents, grandmothers, and other relatives	Leukemia, 0–14 years, 7/1/1974-6/30/1986, Shanghai Cancer Institute Tumor Registry	Controls were selected from the Shanghai general population at random, matched to cases 2:1 on sex and birth calendar year.
Baptiste et al., 1989 [55] (New York State, U.S. (except New York City, Westchester, Rockland, Nassau, and Suffolk counties)	Maternal interview	CNS, 0–14 years, 1/1/1968-12/31/1977, New York State Cancer Registry	Controls were randomly selected from the New York State birth certificate files matched to cases at a 2:1 ratio on birth year, sex, and race.
Birch et al., 1990 [56] (United Kingdom)	Parental interview/medical record verification (ICD-9 coded 740–759)	CNS, 0–14 years, 1980–1983 from regional pediatric oncology centers, West Midlands Regional Cancer Registry, Manchester Children's Tumor Registry, Yorkshire Regional Cancer Registry	Two sets of controls were ascertained from general practitioner lists and hospitals matched to cases on sex and age. The second set excluded children with "a genetic or other constitutional disease or malformation known to be associated with increased risk of cancer" and any other major malformation or chronic disease.
Magnani et al., 1990 [47] (Italy)	Interview of parents/closest relatives	ALL, AML, NHL, NR, 1974–1984, Pediatric Hospital in Turin	Controls were a random sample of children hospitalized at the same hospital as cases.
Kajtár et al., 1990 [79] (Hungary)	Examinations of spine for vertebral anomalies on i.v. pyelograms in cases and X-rays in controls	WT, NR, NR, Department of Pediatrics, University Medical School, Hungary	Controls were children with X-ray for acute abdomen or trauma.
Zack et al., 1991 [40] (Sweden)	The Swedish Medical Birth Registry (ICD-8 codes 740–759)	All leukemias, lymphatic leukemias, myeloid leukemias, other or unspecified leukemias, 0–11 years, 1973–1984, the Swedish National Cancer Registry and Swedish Registry of Causes of Death	Five controls per case were selected from the Swedish Medical Birth Registry matched to cases on sex, birth year and month.
Schumacher et al., 1992 [23] (Germany)	Chest X-ray examination for rib anomalies	Overall, yolk sac carcinoma, OS, HD, histiocytosis X, NHL, ES, WT, STS, ALL, brain tumor, NB, 9 months-21years, NR, University Children's Hospital, Langenbecksrasse	Chest roentgenograms from patients without cancer with a similar age distribution as cases (15 months-14 years) were reviewed for comparison.
Mann et al., 1993 [16] (United Kingdom)	Parental interview/medical record verification (ICD-9 coded)	Overall, ALL, other leukemia, HL, other lymphomas, CNS, STS, bone tumors, WT, NB, RB, HB, GCT, epithelial tumors, Other neoplasms, 0–14 years, 1980–1983, regional pediatric oncology centers, West Midlands Regional Cancer Registry, Manchester Children's Tumor Registry, Yorkshire Regional Cancer Registry	Two sets of controls were selected from general practitioner lists and hospitals matched to cases on sex and age. The second set excluded children with "a genetic or other constitutional disease or malformation known to be associated with increased risk of cancer" and any other major malformation or chronic disease.
Savitz et al., 1994 [15] (U.S.)	Maternal interview or alternate respondent. BDs were recorded verbatim and blindly classified as major, minor, or not a defect.	Overall, ALL, brain, lymphoma, STS, 0–14 years, 1/1/1976-12/31/1983, primary data sources were the Colorado Central Cancer Registry and the Colorado Department of Health [106]	Controls were selected through RDD and frequency matching on location, sex, and age within 3 years.
Cordier et al., 1994 [61] (Ile de France, France)	Maternal interview. The authors noted excluding "minor anomalies such as nevi or birthmarks".	Brain, 0–15 years, 7/1/1985-6/30/1987, medical record abstractions from the neurosurgery, neurology, pediatric, pediatric oncology, or radiology departments at 13 hospitals	Controls were selected from the general population through sampling households on a representative list provided by the census bureau and telephone books at random matched to cases on birth year.
Gold et al., 1994 [64] (U.S.)	Parental interview	Brain, 0–17 years, 1/1/1977-12/31/1981 from eight Surveillance, Epidemiology, and End Results tumor registries	Three controls per case were identified through a variety of methods including RDD, lists of non-institutionalized individuals maintained by the Hawaii Department of Health and through a random sample of households (Pierce County, Washington). Controls were individually matched to cases on age, sex, and mother's racial/ethnic classification.
McCredie et al., 1994 [65] (Australia)	Maternal interview	Brain, 0–14 years, 1985–1989, New South Wales Central Cancer Registry	Two controls per case matched on sex and age were ascertained from eligible women on the Electoral Roll.
Yang et al., 1995 [86] (U.S.)	Parental interview. BDs were classified as major and minor.	RMS, 0–20 years, 4/1982-7/1988, Intergroup RMS study of the Children's Cancer Group and the Pediatric Oncology Group	Controls were ascertained by RDD matched to cases on telephone area code and exchange, sex, age, and race.
Shu et al., 1995 [89] (U.S., Canada, Australia)	Self-administered questionnaire	Malignant GCT, 0–14 years, 1982–1989, Children's Cancer Group	Controls were ascertained by RDD as part of the CCG-E04 pool.
Cnattingius et al., 1995 [49] (Sweden)	Swedish Medical Birth Register (ICD-8 to 1986 and ICD-9 after). Registration was completed upon mother and child leaving the hospital.	Lymphatic leukemia, 0–14 years who were born between 1973 and 1989, diagnoses through 1989, National Cancer Register	Five controls per case were selected from the source population who were alive at the case diagnosis and who were matched on sex, birth year and month.
Cnattingius et al., 1995 [41] (Sweden)	Swedish Medical Birth Register (ICD-8 to 1986 and ICD-9 after). Registration completed when mother and child leave the hospital.	Myeloid leukemia, 0–14 years who were born between 1973 and 1989, diagnoses through 1989, National Cancer Register	Same as Cnattingius et al., 1995 [49]
Adami et al., 1996 [54] (Sweden)	Swedish Medical Birth Register (ICD-8 to 1986 and ICD-9 after).	NHL, 0–14 years who were born between 1973 and 1989, diagnoses through 1989, the National Cancer Register	Same as Cnattingius et al., 1995 [49]
Gelberg et al., 1997 [12] (New York State, U.S., excluding New York City)	Telephone interview with the subject and/or parents, birth certificates and school and medical records	OS, 0–24 years, 1978–1988, the New York State Cancer Registry	Controls were ascertained from New York live birth records and matched to cases at a 1:1 ratio on birth year and sex.
Altmann et al., 1998 [17] (Victoria Australia)	The Victorian Perinatal Data Collection Unit Congenital Malformations/BDs Register (BDs coded according to the British Pediatric Association’s modification of the ICD-9). The authors noted that "the register collects information on both structural defects and chromosomal anomalies at birth…The register excludes certain trivial malformations, such as birth marks, skin tags and hydroceles." BDs were ascertained up to the age of 15 years.	Overall, leukemia (ALL, AML), CNS (astrocytoma), SNS (NB), lymphoma, STS (RMS), renal (WT), RB, germ cell/gonadal, bone, and hepatic, 0–14 years, 1/1/1984 to 12/31/1993, the Victorian Cancer Registrar	Four live born controls per who survived the neonatal period were selected from Victorian births at random and matched to cases on date of birth within 6 months.
Méhes et al., 1998 [52] (Germany)	Physical exam for mild errors of morphogenesis	Leukemia, 7 months-14 years, NR, the Department of Pediatrics, University Medical School of Pécs and the University Children's Hospital, Tübingen, Germany	There were two control comparison groups: 1) children with acute infectious diseases and 2) siblings.
Mertens et al., 1998 [43] (U.S., Canada, Western Australia)	Maternal interview (ICD-9 coded 740–759). Some minor BDs were included.	ALL, AML, cases from three different leukemia studies diagnosed at 0–18 months (CCG-E09), 0–17 years (CCG-E14), 0–14 years, 1983–1994 (CCG-E15), the Children's Cancer Group	Controls were selected from regional populations by a modified RDD method and matched on telephone exchange, age, and race (for E14 and E15).
Buck et al., 2001 [68] (New York State, U.S., excluding New York City)	Parental telephone interview/supplemented with data from birth certificates	NB, 0–5 years, 1976–1987, the New York State Cancer Registry	Controls were randomly selected from live birth registries and frequency matched to cases on birth year.
Infante-Rivard et al., 2001 [42] (Québec, Canada)	Parental interview for major BDs (ICD-9 coded 751–754)	ALL, 0–9 years, 1980–1993, Government-sanctioned tertiary care centers	Controls were selected from family allowance files and were individually matched to cases on age within 3 months, sex, and region of residence at diagnosis.
Roganovic et al., 2002 [50] (Rijeka, Croatia)	NR (minor BDs ascertained)	Hematologic, 5 months-16 years, 1983–1997, the Division of Hematology, Department of Pediatrics, University of Rijeka	Controls were healthy children that were the same age and gender as cases.
Méhes et al., 2003 [80] (Pécs, Hungary)	Abdominal roentgenograms and anteroposterior radiographs for cases; radiography for trauma or acute abdomen in controls; physical exam for both cases and controls. Spinal dysraphism ascertained.	WT, 2 months to 9 years for cases, NR, Department of Pediatrics, University Medical School of Pécs, Pécs, Hungary	Controls were children with radiography for trauma or acute abdomen ranging in age from 1–10 years.
Menegaux et al., 2005 [71] (U.S. and Canada)	Maternal interview (ICD-9 coded 740–759, both major and minor BDs were ascertained).	NB, 0–18 years, 1992–1994, the Children's Oncology Group	One control per case was selected by RDD individually matched to cases on date of birth within 6 months for cases aged <3 years and 1 year for cases aged >3 years.
Merks et al., 2005 [26] (The Netherlands)	Review of chest radiographs for rib anomalies	Overall, NB, GCT, RMS, WT, OS, ES, MB, AST, HD, AML, ALL, NHL, Other malignancies, 0–18 years, 1/1/1998-12/31/2002, Late Effects of Childhood Cancer Clinic and the Emma Children's Hospital, Academic Medical Center for newly diagnosed patients	Controls aged 0–18 years were selected from patient chest radiographs ordered by general practitioners and pediatricians in the outpatient ward and emergency room physicians.
Podvin et al., 2006 [44] (Washington State, U.S.)	Birth records	Leukemia, 0–19 years, 1981–2003, Washington State Cancer Registry and the Cancer Surveillance System of Western Washington	Ten controls without leukemia per case were randomly selected from the birth certificate records and were frequency matched to cases on birth year.
Urayama et al., 2006 [73] (California, U.S.)	Birth certificates	NB, 0–4 years, 1988–1997, California's statewide cancer registry	Two controls per case were randomly selected from birth certificates matched to cases on birth date and gender. Controls were replaced if they died younger than their matched case's diagnosis age.
Chow et al., 2007 [70] (Washington, U.S.)	Birth certificates (ICD-9 codes 740–759, major and minor BDs ascertained). For individuals who were born ≥1987 additional ICD-9 codes for discharges were obtained through linkage of birth certificates to the Comprehensive Hospital Abstract Reporting System.	NB, <20 years, 1993–2004, Washington State Cancer Registry & Cancer Surveillance System (CSS) of Western Washington	Ten controls without NB per case were randomly ascertained and were frequency matched on year of delivery.
Munzer et al., 2007 [69] (France)	Maternal telephone interview (ICD-10 coded Q00-Q99). BDs were classified as minor and major according to the European Surveillance of Congenital Anomalies.	NB, 0–14 years, 1/1/2003-12/31/2004, the National Registry of Hematological Cancer and the National Registry of Childhood Solid Tumors	Controls were randomly selected using phone numbers representative of the French population frequency matched to cases on age and gender.
Loder et al., 2007 [25] (Indiana, U.S.)	Chest radiographs were reviewed for rib number.	Overall, solid, lymphoproliferative, and neural, 1–12 years, malignancies cared for from 2001–2005, Riley’s Children’s Hospital Pediatric Tumor Registry	A similar sized control group identified from the Radiology Department logs was selected from children admitted at the same hospital for polytrauma.
Mallol-Mesnard et al., 2008 [58] (France)	Maternal interview. BDs were classified as minor and major according to the European Surveillance of Congenital Anomalies.	CNS, 0–14 years, 1/1/2003-12/31/2004, National Registry of Childhood Haematological Cancers and the National Registry of Childhood Solid Tumors	Controls were selected from the French population by sampling from 60,000 representative addresses taken from the French national telephone directory plus unlisted phone numbers generated randomly. Age and gender quotas were applied.
Merks et al., 2008 [21] (Amsterdam, Netherlands)	Clinical examination by a physician for major and minor anomalies. BD classification was based on Merks JH et al., 2003 [107].	Overall, 0–18 years, 1/2000-3/2003, Clinic for Late Effects of Childhood Cancer Clinic in the Emma Children's Hospital, Academic Medical Center	Controls were recruited from the city of Haarlem and the surrounding semirural and rural area.
Johnson et al., 2009 [88] (U.S. and Canada)	Maternal interview	GCT, 0–14 years, 1/1/1993-12/31/2001, the Children's Oncology Group	The control group was recruited through RDD and frequency was matched to cases on sex and birth year within 1 year at ratios of approximately 1:2 for males and 1:1 for females.
Johnson et al., 2010 [46] (U.S. and Canada)	Maternal interview. Both major and minor BDs were ascertained.	All infant leukemia, ALL, AML, 0–1 year, 1/1/96-10/13/02 (Phase I) 1/1/03-12/31/06 (Phase II), the Children's Oncology Group	Phase one controls (5/1999-10/2002) were sampled from the population through RDD. Phase two controls (10/2003-3/2008) were selected from state birth registries. Controls were frequency matched to cases on birth year and region of residence based on the phase one case distribution.
Durmaz et al., 2011 [20] (Turkey)	Two medical geneticists qualified in pediatric genetic dysmorphology examined patients for age-independent minor BDs by using the London Dysmorphology database.	Overall, hematopoetic, CNS, WT/GCT, RMS, OS, NR, NR, Cases were diagnosed at Ege University Faculty of Medicine, Izmir, Turkey	The control group was randomly recruited from the Pediatric Outpatient Service at the Ege University Medical Faculty.
Partap et al., 2011 [57] (California, U.S.)	California Office of Vital Records' birth certificate database	CNS, LGG, HGG, MB, PNET, GCT, EPD, 0–14 years, 1988–2006, California Cancer Registry	Four controls that matched to each case on birth date and sex were selected from the California birth certificate database.
Citak et al., 2011 [22] (Turkey)	Two pediatric hematologists/oncologists examined patients for minor BDs using the London Dysmorphology database	ALL, AML, chronic myelocytic leukemia, chronic myelomonocytic leukemia, MDS, 1.5–18 years, NR, cases diagnosed at a single institution	The control group consisted of healthy children of the same age, gender, and ethnicity.
Zierhut et al., 2011 [24] (Minnesota, U.S.)	Radiologists' X-Ray examination for rib anomalies	Overall, all acute leukemia, ALL, AML, lymphoma, CNS, NB, renal, bone tumors, sarcomas, 0–19 years, 2003–2009, the University of Minnesota Medical Center-Fairview	Controls were randomly selected pediatric patients who received a chest X-ray at Fairview Ridges Hospital in Burnsville, MN.
Rudant et al., 2013 [45] (France)	Maternal interview with structured questionnaires. BDs were classified as major and minor according to the European Surveillance of Congenital Anomalies.	All acute leukemia, ALL, AML, 0–14 years, 2003–2004, the National Registry of Childhood Hematopoietic Malignancies	Controls were recruited at random from the telephone directory using gender and age quotas in eight strata reflecting the expected distribution of all the cases.
Citak et al., 2013 [22] (Turkey)	Two pediatric hematologists/oncologists examined patients for minor BDs using the London Dysmorphology Database.	Overall, lymphoma, solid tumors, 0.1–18 years, NR, "2 different institutions"	Controls were selected from individuals seen at the healthy child clinic of Mersin University Hospital and Mersin Obstetric, Gynecology and Children Hospital, Department of Pediatric Hematology and Oncology who were within the same age range, sex, and ethnicity as cases.
Parodi et al., 2014 [72] (Italy)	Parental interview, structured questionnaire (ICD-9 coded 740–759)	NB, 0–10 years, 1998–2001, Pediatric Oncology Centers of the Italian Association of Pediatric Hematology and Oncology (AIEOP)	Controls were randomly selected from the National Health Service database matched on gender, date of birth and area of residence.
Venkatramani et al., 2014 [84] (U.S.)	Maternal interview/ the Utah Population Database (UPDB, ICD-9 codes 740–759)	HB, 0–5 years, 2000–2008, the Children's Oncology Group (COG) for the discovery cohort, the UPDB linked to the State Cancer Registry from 1978–2010 for the validation cohort	The discovery control group was identified from the birth registries from 32 states and matched to cases on birth weight, gender, birth year, and region. The validation control group was selected randomly from the Utah population and matched 10:1 to cases on gender and birth year.
Greenop et al., 2014 [60] (Australia)	Mailed exposure questionnaire	Brain tumors, NR (childhood), 2005–2010, 10 Australian oncology centers	Controls were recruited through random digit dialing and matched to childhood CBT cases on age, sex, and state of residence at a 3:1 ratio [108].
Santos et al., 2016 [109] (Brazil)	Exam for café-au-lait spots by two trained dysmorphologists	Solid tumors (clear cell renal cell carcinoma, CNS, EWS, fibrosarcoma, GCT, HB, OS, RB, RMS, synovial sarcoma, STS, WT), 0–18, NR, NR	Cases were from Rio De Janeiro and Sao Paulo. The control group was comprised of school children from Rio de Janeiro without a diagnosis of cancer or a predisposing syndrome.
Rios et al., 2016 [74] (France)	Maternal telephone interview (ICD-10 coded). Minor BDs or unspecified BDs were excluded according to the European Surveillance of Congenital Anomalies.	NB, <6 years, 2003–2004 (ESCALE) and 2010–2011 (ESTELLE), French National Registry of Childhood Cancer	The analysis was based on pooled data from two French case-control studies (ESTELLE and ESCALE). Controls were frequency matched to cases on sex and age so that there would be at least one control per case.
Hall et al.c, 2017 [90] (California, U.S.)	California birth certificates	GCT, yolk sac tumors, teratomas, ≤5 years, 1988–2013, California Cancer Registry	Controls were randomly selected from California birth records frequency matched to cases on birth year.
Bailey et al., 2017 [59] (France)	Maternal telephone interview	Brain tumors, 0–14 years, 2003–2004 (ESCALE) and 2010–2011 (ESTELLE), French National Registry of Childhood Cancer	Same as Rios et al., 2016 [74].
***Cohort studies***			
Windham et al., 1985 [27] (Norway)	Medical Birth Registry (ICD-8 codes 740–759)	Overall, leukemia, nervous system tumors, renal cancer, eye cancer, NB, 0–13 years, 1967–1980, the Norwegian Cancer Registry	Individuals without BDs from the Norwegian Medical Birth Registry comprised the unexposed group.
Mili et al., 1993 [28] (Georgia, U.S.)	The Metropolitan Atlanta Congenital Defects Program (major BDs, six-digit code for reportable BDs, a modification of the British Pediatric Association Code, which uses a modification of ICD-9 codes). BDs were captured in the first year of life.	Overall, leukemia, brain tumors, NB, WT, RB, 0–14, 1/1/1975-12/31/1988, the Georgia Center for Cancer Statistics at Emory University	The expected number of cancer cases was calculated based on Atlanta Surveillance Epidemiology and End Results rates.
Mili et al., 1993 [29] (Iowa, U.S.)	The Iowa Birth Defects Registry (only major BDs, six-digit code for reportable BDs, a modification of the British Pediatric Association Code, which uses a modification of ICD-9 codes). BDs were captured in the first year of life.	Overall, leukemia, brain tumors, NB, sarcoma, 0–7 years, 1/1/1983-12/31/1989, the State Health Registry of Iowa's Cancer Registry (a SEER registry)	The expected number of cancer cases was calculated based on Iowa Surveillance Epidemiology and End Results rates.
Agha et al., 2005 [33] (Ontario, Canada)	Canadian Congenital Anomalies Surveillance System (ICD-9 codes 740.0–759.9). BDs were captured in the first year of life.	Overall, leukemia, lymphoma, CNS, sympathetic nervous system, RB, renal tumors, bone tumors, STS, GCT, trophoblastic and other gonadal carcinoma, and malignant epithelial, 0–19 years, 1979–1996, the Ontario Cancer Registry	Children without BDs were selected from the Birth Certificate File of Ontario. For every child with a BD, one child without BDs was selected matched on birth year, maternal age, birth order, mother's marital status, and parent's place of birth (Ontario vs. other).
Johnson et al., 2007 [37] (U.S.)	Both maternal interview using standardized tools and medical examinations of the children for birthmarks	Overall, 0–8 years, 1959–1966, the Collaborative Perinatal Project (CPP) subject population	Children without birthmarks in the CPP cohort were used as the comparison group.
Bjørge et al., 2008 [75] (Norway)	The Medical Birth Registry of Norway	NB, 0–14 years, 1967–2004, the Cancer Registry of Norway	The comparison group included all live born children in Norway during 1967–2004 without reported congenital malformations.
Rankin et al., 2008 [30] (Northern Region, United Kingdom)	Northern Congenital Abnormality Survey (ICD-10 coded BDs). The authors note including only "major congenital anomaly subtypes" BDs were captured in the first year of life.	Overall, ALL, AML, other leukemia, HL and NHL, brain, NB, WT, RB, RMS, and others, NR, 1985–2001, the Northern Region Young Persons Malignant Disease Registry	Children without BDs born in the Northern Region were used as the comparison group.
Carozza et al., 2012 [31] (Texas, U.S.)	Texas Birth Defects Registry (1979 British Pediatric Association Classification of Diseases and the 1979 ICD-9-CM, as modified by the U.S. CDC and the Texas Department of State Health Services). Major structural and chromosomal BDs were included. More minor defects were included if the individual also had a major BD. BDs were captured in the first year of life.	Overall, leukemia, lymphoma, CNS, NB, RB, renal tumors, hepatic tumors, malignant bone tumors, STS, GCT, other epithelial, 0–14 years, 1996–2005, the Texas Cancer Registry	All children live born in Texas and not registered in the Texas Birth Defects Registry who were identified through birth certificates were included as the controls.
Fisher et al., 2012 [36] (California, U.S.)	The California Birth Defects Monitoring Program and birth certificates (major BDs were classified based on the British Pediatric Association Classification of Diseases codes, as modified by the CDC). BDs were captured in the first year of life.	Overall, leukemia, lymphoma, CNS, NB, WT, Non-CNS germ cell, RMS, 0–14 years, 1988–2006, the California Cancer Registry	Children without major BDs born from 1988–2004 were used as the comparison group.
Botto et al., 2013 [32] (Utah, Arizona, Iowa, U.S.)	State Birth Defect Surveillance Program (selected major BDs as defined by the National Birth Defect Prevention Network); BD’s were captured up to 15 years of age.	Overall, leukemia, MDS/MPD, lymphoma, brain tumor, NB spectrum, RB, kidney tumor, liver tumor, sarcoma, germ cell, trophoblastic and gonadal tumor, 0–14 years, 1968–2005 for AZ, 1983–2005 for IA, 1994–2008 for Utah, Arizona and Utah state cancer registries	The comparison group included individuals without BDs selected randomly from state birth certificates who were frequency matched 3:1 to the cases by birth year.
Sun et al., 2014 [38] (Denmark)	Danish National Hospital Register (Danish version of ICD-8 codes from 1977–1993: 740–743, 746–747, 759, ICD-10 codes from 1994 onwards Q00-07, Q20-28, Q90-99)	Overall, CNS, mesothelial and soft tissue, skin, lymphatic and haematopoietic, other systems, 0–19 years, 1/1/1977-12/31/2007, Danish Cancer Registry	The comparison group consisted of all children without BDs live born in Denmark between 1977–2007 after excluding missing data, adopted children, twins, and chromosomal anomalies.
Dawson et al., 2015 [34] (Western Australia)	Western Australian Register of Development Anomalies (British Pediatric Association Classification of Diseases, a five-digit extension of ICD-9). BDs were captured in the first year of life.	Overall, leukemia, lymphoma, CNS, NB, RB, renal tumors, hepatic, bone, STS, gonadal and germ cell, other epithelia/melanoma, >90 days-14 years, 1982–2007, Western Australia Cancer Registry	The comparison group included all live born children with > 90 days of follow-up born in Western Australia from 1982–2007 BDs.
Janitz et al., 2016 [35] (Oklahoma, U.S.)	Oklahoma Birth Defects Registry (as defined by the CDC British Pediatric Association codes for congenital anomaly categories and classified according to the National Birth Defects Prevention Network (2004)). BDs were reported up to age 6 years but signs/symptoms must have been present by age 2 years.	Overall, leukemia, lymphoma, CNS, hepatic tumors, STS, GCT, 0–12 years, 1/1/1997-3/31/2009, Oklahoma Central Cancer Registry	The comparison group comprised all singleton births in Oklahoma from 1/1/1997 to 3/31/2009 who were not linked to the Oklahoma Birth Defects Registry.
***Case-cohort studies***			
Johnson et al., 2008 [76] (Minnesota, U.S.)	Minnesota Birth Registry records	NB, 28 days-14 years, 1988–2004, Minnesota Cancer Surveillance System	The sub-cohort was comprised of four individuals per childhood cancer case randomly matched to cases on birth year who were born from 1976–2004.
Puumala et al., 2008 [82] (Minnesota, U.S.)	Minnesota Birth Registry records	WT, 28 days-14 years, 1988–2004, Minnesota Cancer Surveillance System	Same as Johnson et al., 2008 [76]
Spector et al., 2008 [85] (Minnesota, U.S.)	Minnesota Birth Registry records	HB, 28 days-14 years, 1988–2004, Minnesota Cancer Surveillance System	Same as Johnson et al., 2008 [76]
***Nested case-control studies***			
Wanderas et al., 1998 [92] (Norway)	Medical Birth Registry (ICD-8 codes and classifications by the Medical Birth Registry and Statistics Norway). The authors indicate BDs of "all types" were included (Table 2). Recorded “presentation anomalies” (present at birth).	Testicular GCTs, 0–28 years, 1967 to June 1996, Norwegian Cancer Registry	Approximately 100 controls per case were obtained from the Norwegian Birth Registry for the birth period of 1967–1995.
Lindblad et al., 1992 [81] (Sweden)	Swedish Medical Birth Registry. BDs captured up to one month of age.	WT, 0–11 years, 1973–1984, Swedish National Cancer Registry	Five sex and birth month and year matched controls without cancers were selected for each case from the Medical Birth Register.
Linet et al., 1996 [63] (Sweden)	The National Medical Birth Register (ICD-8 codes 740–759)	Brain tumors, 0–17 years, 1973–1989, the National Cancer and Death Registers	Same as Lindblad et al., 1992 [81]
***Case series studies with external comparison groups***			
Breslow et al., 1982 [83] (The National Wilms’ Tumor Study (NWTS))[110]	The NWTS registration form (ICD-9 codes 741–759)	WT, 0–15 years, 10/1969–3/1981, the NWTS Statistical Center; histologic confirmation was available for 75% of the patients	Survey findings were compared to the CPP and results from the CDC Surveillance system on BDs.
Ruymann et al., 1988 [87] (Ohio, U.S.)	Autopsy reports (major and minor ascertained)	RMS, NR, NR, Autopsy results from children in the Intergroup Rhabdomyosarcoma Studies I and II	Rates were compared with those from the NWTS and the CPP.
Narod et al., 1997 [53] (United Kingdom)	A postal questionnaire to family physicians of children diagnosed with cancer and who were alive at the end of 1988 (ICD-9 codes 7400–7599)	Leukemia, lymphoma, brain and spinal cord, NB, RB, WT, liver, OS, ES, STS, gonadal and germ cell, 0–14 years, 1971–1986, National Registry of Childhood Tumors	The expected number of cancer cases was calculated based on two groups: 1) the frequency of BDs among children in the study group, and 2) the frequency of BDs recorded in the British Columbia Health Surveillance Registry (BC Registry).

Studies from 17 countries (Australia, Brazil Canada, China, Croatia, Denmark, France, Germany, Hungary, Italy, the Netherlands, Norway, Sweden, Switzerland, Turkey, the United Kingdom, and the United States) were published from 1958–2017. Study designs included 13 cohort, 58 case-control, 3 case-cohort, 3 nested case-control and 3 case series with external comparison groups. BDs were measured through a variety of methods including interviews, questionnaires, medical records, physical exams, administrative data linkages and from physician interview/report. Cancers were most commonly ascertained through hospital or population-based tumor registries and death certificates.

### Pediatric cancers overall (Table 3)

**Table 3:** Studies on the association between birth defects and childhood cancer.

Reference	Comparison group 1^¥^	Comparison group 2^¥^	Cancer type	Birth defect	Risk estimate (95% CI)	Comments
***Overall case-control studies***						
Stewart et al., 1958 [18]	1,416 (25)	1,416 (25)	Overall	Any BD	1.0 (0.57–1.75)^a^^,^^b^	BDs excluding DS and naevi.
Méhes et al., 1985 [19]	104 (72)	104 (36)	Overall	Minor	4.25 (2.38–7.59)^a^^,^^b^	
Schumacher et al., 1992 [23]	1,000 (242)	200 (11)	Overall	Rib	5.49 (2.94–10.25)^a^^,^^b^	
	1,000 (22)	200 (1)	Overall	Cervical rib left	4.48 (0.60–33.40)^a^^,^^b^	
	1,000 (21)	200 (1)	Overall	Cervical rib right	4.27 (0.57–31.92)^a^^,^^b^	
	1,000 (161)	200 (7)	Overall	Cervical rib bilateral	5.29 (2.44–11.46)^a^^,^^b^	
	1,000 (5)	200 (0)	Overall	Synostoses	ND	
	1,000 (12)	200 (1)	Overall	Aplasia/hypo-	2.42 (0.31–18.69)^a^^,^^b^	
	1,000 (21)	200 (1)	Overall	Bifurcation	4.27 (0.57–31.92)^a^^,^^b^	
Mann et al., 1993 [16]	555 (60)	555 (27)	Overall	Any BD	2.37 (1.48–3.79)^a^^,^^b^	Data not shown for 555 hospital controls.
Savitz et al., 1994 [15]	242 (21)	212 (11)	Overall	Major BD	2.1 (0.9–5.0)^b^	Estimate adjusted for diagnosis year.
Altmann et al., 1998 [17]	570 (55)	2,280 (58)	Overall	Any BD	4.5 (3.1–6.7)^b^	Adjusted for 6-month calendar period, gender, birth weight, gestational age, and maternal age.
	570 (18)	2,280 (5)	Overall	Chromosomal (758)	16.7 (6.1–45.3)^b^	
	570 (6)	2,280 (3)	Overall	Chromosomal excluding DS (758)	9.2 (2.3–37.3)^b^	
	570 (5)	2,280 (3)	Overall	Nervous system (740–742, 340–2, 344, 350–9)	6.5 (1.5–27.8)^b^	
	570 (8)	2,280 (4)	Overall	Cardiac septal/bulbous cords (745)	8.6 (2.6–29.0)^b^	
	570 (3)	2,280 (4)	Overall	Ventricular septal defect (745.40–49)	4.4 (0.9–22.3)^b^	
	570 (4)	2,280 (4)	Overall	Ventricular septal defect excluding DS (745.40–49)	4.1 (1.0–16.8)^b^	
	570 (9)	2,280 (7)	Overall	Other heart/circulatory system (746–747)	5.5 (2.0–15.0)^b^	
	570 (6)	2,280 (7)	Overall	Other heart circulatory excluding DS (746–747)	3.6 (1.2–10.8)^b^	
	570 (3)	2,280 (1)	Overall	Respiratory system (748)	14.5 (1.5–142)^b^	
	570 (5)	2,280 (3)	Overall	Eye/face/neck (743, 744)	7.3 (1.7–30.9)^b^	
	570 (7)	2,280 (11)	Overall	Gastrointestinal system (750,751)	3.3 (1.2–9.0)^b^	
	570 (13)	2,280 (22)	Overall	Musculoskeletal system (754–756)	2.7 (1.3–5.4)^b^	
	570 (4)	2,280 (5)	Overall	Congenital dislocation of hip (754.30)	3.2 (0.9–12.5)^b^	
	570 (6)	2,280 (9)	Overall	Genitourinary system (752–753)	2.9 (1.0–8.1)^b^	
	570 (3)	2,280 (5)	Overall	Hypospadias (752.60)	2.6 (0.6–10.9)^b^	
	570 (2)	2,280 (1)	Overall	Endocrine/metabolic (240–279)	8.4 (0.8–93.2)^b^	
	570 (2)	2,280 (2)	Overall	Cleft lip and/or palate (749)	9.0 (0.8–100)^b^	
Merks et al., 2005 [26]	906 (78)	881 (54)	Overall	Cervical rib	1.44 (1.01–2.07)^a^^,^^b^	
	906 (48)	881 (58)	Overall	Aplasia 12th ribs	0.79 (0.54–1.18)^a^^,^^b^	
	906 (8)	881 (8)	Overall	Lumbar ribs	0.97 (0.36–2.60)^a^^,^^b^	
	906 (5)	881 (6)	Overall	Bifurcation	0.81 (0.25–2.66)^a^^,^^b^	
	906 (2)	881 (3)	Overall	Synostosis-Bridging	0.65 (0.11–3.88)^a^^,^^b^	
	906 (1)	881 (0)	Overall	Segmentation	ND	
Loder et al., 2007 [25]	218 (39)	200 (16)	Overall	Abnormal rib number (normal = 24 ribs)	2.5 (1.4–4.6)^b^	Children with a known BD or DS were excluded.
Merks et al., 2008 [21]	903 (65)	923 (6)	Overall	Blepharophimosis	11.1 (4.8–25.4)^b^	Cases and controls were examined by two different observers. Although the authors report that "11% of controls and 7% of patients were scored independently by 2 observers, resulting in high scores", they do not report the data associated with this comment.
	903 (58)	923 (2)	Overall	Asymmetric lower limbs	29.6 (7.3–121.0)^b^	
	903 (30)	923 (3)	Overall	Sydney crease	10.2 (3.1–33.4)^b^	
	903 (26)	923 (3)	Overall	Broad foot	8.9 (2.7–29.2)^b^	
	903 (14)	923 (0)	Overall	Isolated short metatarsals	∞ (1.8-∞)^b^	
	903 (13)	923 (0)	Overall	Short distal phalanx of thumb	∞ (1.6-∞)^b^	
	903 (18)	923 (2)	Overall	Port-wine stain (Major anomaly)	9.2 (2.1–39.5)^b^	
	903 (12)	923 (0)	Overall	Hyperconvex nails	∞ (1.5-∞)^b^	
	903 (43)	923 (16)	Overall	Retrognathia	2.8 (1.6–4.8)^b^	
	903 (25)	923 (6)	Overall	Hypoplastic alae nasi	4.3 (1.8–10.3)^b^	
	903 (52)	923 (24)	Overall	Prominent ears	2.2 (1.4–3.6)^b^	
	903 (15)	923 (2)	Overall	Broad hand	7.7 (1.8–33.4)^b^	
	903 (32)	923 (12)	Overall	Scoliosis	2.7 (1.4–5.3)^b^	
	903 (51)	923 (26)	Overall	Hypertelorism	2.0 (1.3–3.2)^b^	
	903 (15)	923 (3)	Overall	Tall stature	5.1 (1.5–17.6)^b^	
	903 (18)	923 (3)	Overall	Microcephaly	6.1 (1.8–20.8)^b^	
	903 (13)	923 (3)	Overall	Macrocephaly	4.4 (1.3–15.5)^b^	
Durmaz et al., 2011 [20]	200 (192^a^)	200 (70^a^)	Overall	Any minor	44.6 (20.7–95.7)^a^^,^^b^	
Zierhut et al., 2011 [24]	455 (31)	1,133 (51)	Overall	Any rib	1.60 (1.00–2.65)^b^	Estimates adjusted for sex and age.
	455 (29)	1,133 (47)	Overall	Rib number (<24 or >24)	1.66 (1.00–2.74)^b^	
	455 (6)	1,133 (9)	Overall	Cervical ribs	1.63 (0.55–4.80)^b^	
Citak et al., 2013 [22]	116 (24)	116 (6)	Overall	Eye	4.78 (1.88–12.20)^b^	Minor anomalies for major sites are presented; includes only patients with lymphoma and solid tumors.
	116 (46)	116 (7)	Overall	Ear	10.23 (4.37–23.94)^b^	
	116 (75)	116 (21)	Overall	Mouth	8.28 (4.51–15.18)^b^	
	116 (35)	116 (5)	Overall	Hand	9.59 (3.60–25.56)^b^	
	116 (41)	116 (8)	Overall	Feet	7.38 (3.27–16.64)^b^	
***Overall cohort studies***						
Windham et al., 1985 [27]	42 (22,856)	NA	Overall	Any BD	1.9 (1.4–2.5)^c^*	Did not exclude any known genetic syndromes. Estimates are age standardized.
	NR (NR)	NA	Overall	Any BD (Male)	1.7^c^*	
	NR (NR)	NA	Overall	Any BD (Female)	2.1^c^*	
	NR (NR)	NA	Overall (0–4 years)	Any BD (Male)	1.5^c^	
	NR (NR)	NA	Overall (0–4 years)	Any BD (Female)	2.4^c^*	
	NR (NR)	NA	Overall (5–9 years)	Any BD (Male)	2.7^c^*	
	NR (NR)	NA	Overall (5–9 years)	Any BD (Female)	1.5^c^	
Mili et al., 1993 [28]	31 (19,373)	400 (524,931)	Overall	Any BD	2.2 (1.5–3.2)^d^	Did not exclude any known genetic syndromes. Estimates adjusted for age. The authors also note adjusting for sex and race in some analyses.
Mili et al., 1993 [29]	16 (10,891)	290 (241,473)	Overall	Any BD	2.0 (1.2–3.3)^d^	Did not exclude any known genetic syndromes. Estimates adjusted for age and sex. The authors note that "by age 5 years, the risk of cancer for children with BDs was 2.0 times the risk for the general population; by age 8 years, the risk of cancer for children with BDs was 1.5 times the risk for the general population."
Agha et al., 2005 [33]	139 (45,200)	73 (45,200)	Overall	Any BD	2.0 (1.8–2.4)^c^	Did not exclude any known genetic syndromes.
	33 (NR)	6 (NR)	Overall (<1 year)	Any BD	5.8 (3.7–9.1)^c^	
Johnson et al., 2007 [37]	5 (NR)	40 (NR)	Overall (0–8 years)	Birthmarks	2.81 (1.11–7.13)^e^	Individuals with suspected birthmarks were excluded. The authors note that none of the individuals were recorded as having a genetic syndrome.
	3	33 (NR)	Overall (1–8 years)	Birthmarks	2.03 (0.62–6.62)^e^	
Rankin et al., 2008 [30]	39 (NR)	812^a^ (NR)	Overall	Any BD	2.86 (2.11–3.89)^f^; 1.8 (1.2–2.7)^f^ DS excluded	Four cases of DS were observed in the cohort.
Carozza et al., 2012 [31]	239 (115,686)	2,112 (3,071,255^a^)	Overall	Any BD	3.05 (2.65–3.50)^f^	Genetic syndromes were not excluded. The authors note that excluding subjects with chromosomal anomalies resulted in a lower IRR for leukemia and total cancers but not for other cancers.
	17 (115,686)	NR (3,071,255^a^)	Overall	CNS	3.61 (2.10–5.79)^f^	
	4 (115,686)	NR (3,071,255^a^)	Overall	Neural tube	3.03 (0.83–7.78)^f^	
	6 (115,686)	NR (3,071,255^a^)	Overall	Eye and ear	3.47 (1.27–7.56)^f^	
	5 (115,686)	NR (3,071,255^a^)	Overall	Anophthalmia/microphthalmia	6.91 (2.24–16.14)^f^	
	91 (115,686)	NR (3,071,255^a^)	Overall	Cardiac and circulatory	3.50 (2.81–4.31)^f^	
	7 (115,686)	NR (3,071,255^a^)	Overall	Conotruncal	3.14 (1.26–6.47)^f^	
	60 (115,686)	NR (3,071,255^a^)	Overall	Septal	3.05 (2.32–3.94)^f^	
	54 (115,686)	NR (3,071,255^a^)	Overall	Left ventricular outflow tract	4.22 (3.16–5.53)^f^	
	5 (115,686)	NR (3,071,255^a^)	Overall	Respiratory	3.58 (1.16–8.36)^f^	
	11 (115,686)	NR (3,071,255^a^)	Overall	Oral clefts	2.69 (1.34–4.82)^f^	
	13 (115,686)	NR (3,071,255^a^)	Overall	Gastrointestinal	1.69 (0.90–2.89)^f^	
	5 (115,686)	NR (3,071,255^a^)	Overall	Gastrointestinal atresia/stenosis	2.56 (0.83–5.97)^f^	
	34 (115,686)	NR (3,071,255^a^)	Overall	Genitourinary	2.37 (1.64–3.32)^f^	
	5 (115,686)	NR (3,071,255^a^)	Overall	Musculoskeletal	0.88 (0.29–2.06)^f^	
	1 (115,686)	NR (3,071,255^a^)	Overall	Limb reduction	0.80 (0.02–4.49)^f^	
	3 (115,686)	NR (3,071,255^a^)	Overall	Abdominal wall	2.26 (0.47–6.62)^f^	
	55 (115,686)	NR (3,071,255^a^)	Overall	Chromosomal (includes trisomies 21, 13, and 18)	15.52 (11.66–20.27)^f^	
Fisher et al., 2012 [36]	132 (59,258)	NR (NR)	Overall	Non-chromosomal	1.58 (1.33–1.87)^e^	Children with leukemia were excluded from the non-chromosomal analysis. Results for specific BDs exclude children with chromosomal anomalies.
	90 (6,327)	NR (NR)	Overall	Chromosomal	12.44 (10.10–15.32)^e^	
	0 (488)	NR (NR)	Overall	Amniotic bands (658)	-	
	0 (423)	NR (NR)	Overall	Anencephalus (740)	-	
	3 (1,124)	NR (NR)	Overall	Spina bifida (741)	3.19 (1.03–9.89)^e^	
	35 (7,678)	NR (NR)	Overall	Other BD of nervous system (742)	5.83 (4.18–8.14)^e^	
	24 (6,392)	NR (NR)	Overall	BD of eye (743)	4.90 (3.28–7.32)^e^	
	26 (11, 025)	NR (NR)	Overall	BD of ear, face, neck (744)	2.94 (2.00–4.33)^e^	
	25 (10,151)	NR (NR)	Overall	Bulbus cordis anomaly/cardiac septal closure (745)	3.36 (2.27–4.98)^e^	
	23 (7,990)	NR (NR)	Overall	Other BD of heart (746)	3.99 (2.65–6.01)^e^	
	21 (6,840)	NR (NR)	Overall	Other BD of circulatory system (747)	4.28 (2.79–6.57)^e^	
	24 (7,958)	NR (NR)	Overall	BD of respiratory system (748)	4.25 (2.85–6.35)^e^	
	5 (4,662)	NR (NR)	Overall	Cleft lip and palate (749)	1.25 (0.52–3.02)^e^	
	23 (10,608)	NR (NR)	Overall	Other BD of upper alimentary (750)	2.45 (1.62–3.69)^e^	
	11 (5,419)	NR (NR)	Overall	Other BD of digestive system (751)	2.44 (1.35–4.40)^e^	
	22 (9,522)	NR (NR)	Overall	BD of genital organs (752)	2.62 (1.72–3.98)^e^	
	15 (5,989)	NR (NR)	Overall	BD or urinary system (753)	3.15 (1.90–5.23)^e^	
	23 (10,263)	NR (NR)	Overall	Certain congenital musculoskeletal deformities (754)	2.56 (1.70–3.86)^e^	
	23 (11,329)	NR (NR)	Overall	Other BD of limbs (755)	2.37 (1.57–3.57)^e^	
	30 (11,168)	NR (NR)	Overall	Certain congenital musculoskeletal anomaly (756, excluding 754)	3.37 (2.35–4.83)^e^	
	31 (11,444)	NR (NR)	Overall	BD of integument (757)	3.09 (2.17–4.40)^e^	
	15 (3,161)	NR (NR)	Overall	Other and unspecified BD (759)	7.80 (4.70–12.94)^e^	
Botto et al., 2013 [32]	123 (44,151)	161 (147,940)	Overall	Any BD	2.9 (2.3–3.7)^f^	Children with chromosomal anomalies were excluded. The relative hazard of cancer for children with non-chromosomal anomalies was highest in the first 3 years of life (figure 3).
	77 (39,726)	161 (147,940)	Overall	Non-chromosomal	2.0 (1.5–2.6)^f^	
	9 (4,311)	161 (147,940)	Overall	Brain	2.2 (1.1–4.3)^f^	
	<5 (1,334)	161 (147,940)	Overall	Neural tube defects, all	2.3 (0.7–7.3)^f^	
	<5 (1,108)	161 (147,940)	Overall	Spina bifida w/out anencephalus	2.7 (0.9–8.4)^f^	
	0 (226)	161 (147,940)	Overall	Encepahalocele	0.0^f^	
	5 (1,801)	161 (147,940)	Overall	Microcephaly	2.9 (1.2–7.1)^f^	
	<5 (63)	161 (147,940)	Overall	Holoprosencephaly	24.5 (3.4–175.3)^f^	
	<5 (1,271)	161 (147,940)	Overall	Hydrocephalus (no spina bifida)	0.9 (0.1–6.3)^f^	
	8 (928)	161 (147,940)	Overall	Eye	9.4 (4.6–19.1)^f^	
	<5 (432)	161 (147,940)	Overall	Anophtalmia/microphthalmia	8.4 (2.7–26.3)^f^	
	6 (530)	161 (147,940)	Overall	Congenital cataract	11.6 (5.1–26.1)^f^	
	<5 (31)	161 (147,940)	Overall	Aniridia	30.8 (4.3–219.9)^f^	
	<5 (626)	161 (147,940)	Overall	Ear (anotia/microtia)	1.7 (0.2–11.9)^f^	
	<5 (422)	161 (147,940)	Overall	Craniosynotosis	3.4 (0.5–24.4)^f^	
	28 (11,211)	161 (147,940)	Overall	Heart	2.9 (1.9–4.3)^f^	
	<5 (465)	161 (147,940)	Overall	Complex heart defects	3.6 (0.5–25.6)f	
	<5 (172)	161 (147,940)	Overall	Common truncus	13.1 (1.8–93.3)^f^	
	<5 (687)	161 (147,940)	Overall	Transposition of great arteries	3.7 (0.9–14.9)^f^	
	<5 (759)	161 (147,940)	Overall	Tetralogy of Fallot	1.6 (0.2–11.4)^f^	
	<5 (350)	161 (147,940)	Overall	Atrioventricular septal defect (AV canal)	9.1 (2.2–36.6)^f^	
	<5 (64)	161 (147,940)	Overall	Total anomalous pulmonary venous return	20.5 (2.9–146.4)^f^	
	0 (182)	161 (147,940)	Overall	Pulmonary valve atresia	0.0^f^	
	0 (153)	161 (147,940)	Overall	Tricuspid valve atresia and stenosis	0.0^f^	
	0 (141)	161 (147,940)	Overall	Ebstein anomaly	0.0^f^	
	0 (509)	161 (147,940)	Overall	Hypoplastic left heart syndrome	0.0^f^	
	<5 (1,024)	161 (147,940)	Overall	Coarctation of the aorta	1.1 (0.1–7.6)^f^	
	<5 (540)	161 (147,940)	Overall	Aortic valve stenosis	4.5 (1.1–18.1)^f^	
	0 (203)	161 (147,940)	Overall	Other major congenital heart defects	0.0^f^	
	<5 (1,037)	161 (147,940)	Overall	Pulmonary valve stenosis	2.2 (0.5–8.9)^f^	
	5 (2,330)	161 (147,940)	Overall	Ventricular septal defect, membranous	2.5 (1.0–6.0)^f^	
	<5 (1,276)	161 (147,940)	Overall	Ventricular septal defect, NOS	2.2 (0.8–6.1)^f^	
	6 (1,404)	161 (147,940)	Overall	Atrial septal defect	5.1 (2.2–11.4)^f^	
	6 (4,756)	161 (147,940)	Overall	Orofacial	1.3 (0.6–2.8)^f^	
	5 (1,656)	161 (147,940)	Overall	Cleft palate/without cleft lip	3.2 (1.3–7.9)^f^	
	<5 (3,100)	161 (147,940)	Overall	Cleft lip with or without cleft palate	0.3 (0–2.2)^f^	
	0 (387)	161 (147,940)	Overall	Choanal atresia	0.0^f^	
	13 (7,207)	161 (147,940)	Overall	Gastrointestinal (GI)	1.7 (1.0–3.0)^f^	
	<5 (1,367)	161 (147,940)	Overall	GI atresias, all	2.4 (0.8–7.6)^f^	
	<5 (562)	161 (147,940)	Overall	Esophageal atresia/TE Fistula	2.0 (0.3–14)^f^	
	0 (67)	161 (147,940)	Overall	Duodenal atresia	0.0^f^	
	0 (79)	161 (147,940)	Overall	Jejunal/Ileal atresia	0.0^f^	
	0 (9)	161 (147,940)	Overall	Small intestinal atresia	0.0^f^	
	<5 (1,025)	161 (147,940)	Overall	Rectal/intestinal atresia/stenosis	3.2 (1.0–10)^f^	
	6 (5,071)	161 (147,940)	Overall	Pyloric stenosis	1.1 (0.5–2.4)^f^	
	<5 (377)	161 (147,940)	Overall	Hirschsprung disease	5.2 (1.3–20.8)^f^	
	<5 (163)	161 (147,940)	Overall	Bilary atresia	15.2 (3.8–61.3)^f^	
	<5 (1,479)	161 (147,940)	Overall	Abdominal wall defects and variants	2.3 (0.7–7.2)^f^	
	<5 (368)	161 (147,940)	Overall	Omphalocele	3.2 (0.4–22.8)^f^	
	<5 (972)	161 (147,940)	Overall	Gastroschisis	2.3 (0.6–9.3)^f^	
	0 (17)	161 (147,940)	Overall	Cloacal exstrophy	0.0^f^	
	0 (66)	161 (147,940)	Overall	Bladder exstrophy	0.0^f^	
	0 (131)	161 (147,940)	Overall	Epispadias	0.0^f^	
	<5 (605)	161 (147,940)	Overall	Diaphragmatic hernia	5.3 (1.3–21.5)^f^	
	20 (10,877)	161 (147,940)	Overall	Genitourinary (GU)	1.8 (1.2–2.9)^f^	
	12 (4,349)	161 (147,940)	Overall	Renal, all	3.3 (1.8–5.9)^f^	
	5 (977)	161 (147,940)	Overall	Renal agenesis/hypoplasia	8.3 (3.4–20.2)^f^	
	8 (3,561)	161 (147,940)	Overall	Obstructive GU defect	2.5 (1.2–5.1)^f^	
	8 (6,691)	161 (147,940)	Overall	Hypospadias (includes 1^st^ degree)	1.1 (0.5–2.2)^f^	
	<5 (1,019)	161 (147,940)	Overall	Limb deficiencies	1.9 (0.5–7.7)^f^	
	<5 (663)	161 (147,940)	Overall	Transverse	3.0 (0.7–12.2)^f^	
	<5 (299)	161 (147,940)	Overall	Preaxial	4.0 (0.6–28.2)^f^	
	0 (332)	161 (147,940)	Overall	Postaxial	0.0^f^	
	0 (439)	161 (147,940)	Overall	Limb deficiency, Not elsewhere classfied/Not otherwise specified	0.0^f^	
	218 (0)	161 (147,940)	Overall	Amniotic bands	0.0^f^	
	46 (4,425)	161 (147,940)	Overall	Chromosomal	13.2 (9.5–18.3)^f^	
Sun et al., 2014 [38]	14 (4,484)	207 (1,547,126)	Overall (<1 year)	Nervous system (cohort entry on day of birth)	23.41 (13.62–40.22)^e^	Children with chromosomal anomalies were excluded. Estimates adjusted for calendar year and sex.
	NR (NR)	NR (NR)	Overall (<1 year)	Nervous system (cohort entry at BD diagnosis)	23.86 (11.23–50.70)^e^	
	36 (4,484)	1,824 (1,547,126)	Overall (1–15 years)	Nervous system (cohort entry on day of birth)	6.58 (4.73–9.16)^e^	
	NR (NR)	NR (NR)	Overall (1–15 years)	Nervous system (cohort entry at BD diagnosis)	5.55 (3.7–8.23)^e^	
	12 (24,643)	<207 (1,547,126)	Overall (<1 year)	Circulatory system (cohort entry on day of birth)	3.64 (2.03–6.51)^e^	
	NR (NR)	NR (NR)	Overall (<1 year)	Circulatory system (cohort entry at BD diagnosis)	1.12 (0.28–4.51)^e^	
	93 (4,484)	1,824 (1,547,126)	Overall (1–15 years)	Circulatory system (cohort entry on day of birth)	3.31 (2.68–4.07)^e^	
	NR (NR)	NR (NR)	Overall (1–15 years)	Circulatory system (cohort entry at BD diagnosis)	1.36 (0.95–1.94)^e^	
Dawson et al., 2015 [34]	94 (32,310)	894^a^ (608,726^a^)	Overall	Any BD	1.96 (1.57–2.43)^e^	Any BD included all BDs. Other risk estimates excluded syndromes known to be associated with cancer (e.g. DS) (N category).
	3 (1,062)	894^a^ (608,726^a^)	Overall	Chromosomal	2.03 (0.66–6.31)^e^	
	54 (31,211)	894^a^ (608,726^a^)	Overall	Non-chromosomal	1.16 (0.88–1.53)^e^	
	0 (1,409)	894^a^ (608,726^a^)	Overall	Nervous	0, -^e^	
	0 (645)	894^a^ (608,726^a^)	Overall	Eye	0, -^e^	
	3 (1,479)	894^a^ (608,726^a^)	Overall	Ear, face, neck	1.41 (0.46–4.38)^e^	
	14 (5,617)	894^a^ (608,726^a^)	Overall	Cardiovascular	1.76 (1.04–2.99)^e^	
	1 (228)	894^a^ (608,726^a^)	Overall	Respiratory	3.64 (0.52–25.63)^e^	
	3 (3,318)	894^a^ (608,726^a^)	Overall	Gastrointestinal	0.61 (0.20–1.88)^e^	
	14 (9,577)	894^a^ (608,726^a^)	Overall	Urogenital	0.97 (0.57–1.65)^e^	
	14 (8,222)	894^a^ (608,726^a^)	Overall	Musculoskeletal	1.14 (0.67–1.93)^e^	
	7 (2,348)	894^a^ (608,726^a^)	Overall	Skin	1.98 (0.94–4.17)^e^	
	5 (3,307)	894^a^ (608,726^a^)	Overall	Other	1.04 (0.43–2.51)^e^	
	57 (32,273)	894^a^ (608,726^a^)	Overall (all ages > 90 days of follow-up)	N BDs	1.19 (0.91–1.56)^e^	
	44 (32,273)	468^a^ (608,726^a^)	Overall (3 months– 4 years)	N BDs	1.74 (1.28–2.37)^e^	
	7 (32,273)	83^a^ (608,726 ^a^)	Overall (3–12 months)	N BDs	1.59 (0.73–3.43)^e^	
	37 (31,368)	385^a^ (586,277^a^)	Overall (1–4 years)	N BDs	1.77 (1.26–2.48)^e^	
	12 (26,598)	232^a^ (485,396^a^)	Overall (5–9 years)	N BDs	0.95 (0.53–1.70)^e^	
	1 (19,590)	194^a^ (367,319^a^)	Overall (10–14 years)	N BDs	0.10 (0.01–0.72)^e^	
Janitz et al., 2016 [35]	56 (23,368)	475 (567,867)	Overall	Any BD (w/chromosomal)	3.0 (2.2–3.9)^e^^†^	HRs were estimated at each age. Specific type BD analyses exclude children with chromosomal anomalies unless specifically noted that they are included (w/chromosomal). The authors note that the proportional hazards assumption was not met for the overall association and that a continuous time interaction model was used for those noted with an ‘^†^’. The HRs for genitourinary and musculoskeletal(non-chromosomal) were adjusted for maternal education and prenatal care, respectively.
	NA	NA	Overall (<1 year)	Any BD (w/chromosomal)	14.1 (8.3–23.7)^e^	
	NA	NA	Overall (3 years)	Any BD (w/chromosomal)	2.3 (1.6–3.2)^e^	
	NA	NA	Overall (6 years)	Any BD (w/chromosomal)	1.1 (0.7–1.9)^e^	
	NA	NA	Overall (9 years)	Any BD (w/chromosomal)	0.8 (0.4–1.4)^e^	
	NA	NA	Overall (12 years)	Any BD (w/chromosomal)	0.6 (0.3–1.1)^e^	
	12^a^ (1,259)	NR (NR)	Overall	Chromosomal	11.9 (6.7–12.2)^e^^†^	
	NA	NA	Overall (<1 year)	Chromosomal	84.7 (33.8–211.9)^e^	
	NA	NA	Overall (3 years)	Chromosomal	7.3 (3.2–16.6)^e^	
	NA	NA	Overall (6 years)	Chromosomal	2.9 (0.8–9.6)^e^	
	NA	NA	Overall (9 years)	Chromosomal	1.6 (0.4–7.1)^e^	
	NA	NA	Overall (12 years)	Chromosomal	1.1 (0.2–5.9)^e^	
	NR (2,143)	NR (NR)	Overall	CNS (w/chromosomal)	3.0 (1.2–7.2)^e^^†^	
	NA	NA	Overall (<1 year)	CNS (w/chromosomal)	18.8 (4.6–77.8)^e^	
	NA	NA	Overall (3 years)	CNS (w/chromosomal)	2.0 (0.6–6.5)^e^	
	NA	NA	Overall (6 years)	CNS (w/chromosomal)	0.8 (0.1–4.9)^e^	
	NA	NA	Overall (9 years)	CNS (w/chromosomal)	0.5 (0.1–4.3)^e^	
	NA	NA	Overall (12 years)	CNS (w/chromosomal)	0.3 (0.0–4.0)^e^	
	NR (1,179)	NR (NR)	Overall	Eye/Ear (w/chromosomal)	3.6 (1.4–9.7)^e^^†^	
	NA	NA	Overall (<1 year)	Eye/Ear (w/chromosomal)	27.9 (6.1–127.0)^e^	
	NA	NA	Overall (3 years)	Eye/Ear (w/chromosomal)	2.2 (0.5–9.1)^e^	
	NA	NA	Overall (6 years)	Eye/Ear (w/chromosomal)	0.8 (0.1–6.8)^e^	
	NA	NA	Overall (9 years)	Eye/Ear (w/chromosomal)	0.4 (0.0–5.9)^e^	
	NA	NA	Overall (12 years)	Eye/Ear (w/chromosomal)	0.3 (0.0–5.4)^e^	
	NR (7,059)	NR (NR)	Overall	Cardiovascular (w/chromosomal)	4.4 (2.9–6.6)^e^^†^	
	NA	NA	Overall (<1 year)	Cardiovascular (w/chromosomal)	22.5 (11.0–46.1)^e^	
	NA	NA	Overall (3 years)	Cardiovascular (w/chromosomal)	3.2 (1.9–5.4)^e^	
	NA	NA	Overall (6 years)	Cardiovascular (w/chromosomal)	1.5 (0.7–3.2)^e^	
	NA	NA	Overall (9 years)	Cardiovascular (w/chromosomal)	0.9 (0.4–2.5)^e^	
	NA	NA	Overall (12 years)	Cardiovascular (w/chromosomal)	0.7 (0.2–2.1)^e^	
	NR (1997)	NR (NR)	Overall	Orofacial (w/chromosomal)	2.5 (0.9–6.6)^e^	
	NR (3,736)	NR (NR)	Overall	Gastrointestinal (w/chromosomal)	2.4 (1.4–5.1)^e^^†^	
	NA	NA	Overall (<1 year)	Gastrointestinal (w/chromosomal)	21.1 (7.0–63.2)^e^	
	NA	NA	Overall (3 years)	Gastrointestinal (w/chromosomal)	1.1 (0.3–3.8)^e^	
	NA	NA	Overall (6 years)	Gastrointestinal (w/chromosomal)	0.3 (0.1–2.2)^e^	
	NA	NA	Overall (9 years)	Gastrointestinal (w/chromosomal)	0.2 (0.0–1.6)^e^	
	NA	NA	Overall (12 years)	Gastrointestinal (w/chromosomal)	0.1 (0.0–1.3)^e^	
	NR (4,124)	NR (NR)	Overall	Genitourinary (w/chromosomal)	2.5 (1.3–5.1)^e^^†^	
	NA	NA	Overall (<1 year)	Genitourinary (w/chromosomal)	15.6 (5.0–48.6)^e^	
	NA	NA	Overall (3 years)	Genitourinary (w/chromosomal)	1.6 (0.6–4.3)^e^	
	NA	NA	Overall (6 years)	Genitourinary (w/chromosomal)	0.7 (0.2–2.9)^e^	
	NA	NA	Overall (9 years)	Genitourinary (w/chromosomal)	0.4 (0.1–2.4)^e^	
	NA	NA	Overall (12 years)	Genitourinary (w/chromosomal)	0.3 (0.0–2.1)^e^	
	NR (6,825)	NR (NR)	Overall	Musculoskeletal (w/chromosomal)	1.2 (0.6–2.6)^e^^†^	
	NA	NA	Overall (<1 year)	Musculoskeletal (w/chromosomal)	5.3 (1.4–20.5)^e^	
	NA	NA	Overall (3 years)	Musculoskeletal (w/chromosomal)	1.0 (0.4–2.5)^e^	
	NA	NA	Overall (6 years)	Musculoskeletal (w/chromosomal)	0.6 (0.2–2.0)^e^	
	NA	NA	Overall (9 years)	Musculoskeletal (w/chromosomal)	0.4 (0.1–1.9)^e^	
	NA	NA	Overall (12 years)	Musculoskeletal (w/chromosomal)	0.3 (0.0–1.8)^e^	
	44 (22,109^a^)	475 (567,867)	Overall	Non-chromosomal	2.5 (1.8–3.3)^e^^†^	
	NA	NA	Overall (<1 year)	Non-chromosomal	10.7 (6.0–19.1)^e^	
	NA	NA	Overall (3 years)	Non-chromosomal	2.0 (1.4–2.9)^e^	
	NA	NA	Overall (6 years)	Non-chromosomal	1.0 (0.6–1.8)^e^	
	NA	NA	Overall (9 years)	Non-chromosomal	0.7 (0.4–1.4)^e^	
	NA	NA	Overall (12 years)	Non-chromosomal	0.5 (0.3–1.2)^e^	
	NR (1,998)	NR (NR)	Overall	CNS	1.9 (0.6–5.9)^e^^†^	
	NR (1,179)	NR (NR)	Overall	Eye/Ear	2.0 (0.5–8.1)^e^	
	NR (3,369)	NR (NR)	Overall	Cardiovascular	2.8 (1.7–4.8)^e^^†^	
	NA	NA	Overall (<1 year)	Cardiovascular	11.1 (4.2–29.4)^e^	
	NA	NA	Overall (3 years)	Cardiovascular	2.4 (1.3–4.4)^e^	
	NA	NA	Overall (6 years)	Cardiovascular	1.3 (0.5–3.2)^e^	
	NA	NA	Overall (9 years)	Cardiovascular	0.9 (0.3–2.8)^e^	
	NA	NA	Overall (12 years)	Cardiovascular	0.7 (0.2–2.6)^e^	
	NR (1,887)	NR (NR)	Overall	Orofacial	2.6 (1.0–6.9)^e^	
	NR (3,552)	NR (NR)	Overall	Gastrointestinal	2.2 (1.0–4.9)^e^^†^	
	NA	NA	Overall (<1 year)	Gastrointestinal	18.5 (5.7–60.2)^e^	
	NA	NA	Overall (3 years)	Gastrointestinal	1.0 (0.3–3.8)^e^	
	NA	NA	Overall (6 years)	Gastrointestinal	0.3 (0.0–2.4)^e^	
	NA	NA	Overall (9 years)	Gastrointestinal	0.2 (0.0–1.8)^e^	
	NA	NA	Overall (12 years)	Gastrointestinal	0.1 (0.0–1.5)^e^	
	NR (3,932)	NR (NR)	Overall	Genitourinary	1.7 (0.7–4.0)^e^^†^	
	NA	NA	Overall (<1 year)	Genitourinary	3.6 (0.5–26.4)^e^	
	NA	NA	Overall (3 years)	Genitourinary	1.2 (0.4–3.6)^e^	
	NA	NA	Overall (6 years)	Genitourinary	0.8 (0.2–3.8)^e^	
	NA	NA	Overall (9 years)	Genitourinary	0.7 (0.1–4.5)^e^	
	NA	NA	Overall (12 years)	Genitourinary	0.6 (0.1–5.1)^e^	
	NR (6,575)	NR (NR)	Overall	Musculoskeletal	1.3 (0.6–2.8)^e^	
***Leukemias*, *myeloproliferative diseases*, *myelodysplastic diseases*, *and lymphoma case-control studies***						
Ager et al., 1965 [39]	112 (5)	Siblings: 105 (3); Neighborhood: 112 (1)	Leukemia	Any BD	Siblings: 1.59 (0.37–6.82)^a^^,^^b^; Neighborhood: 5.19 (0.60–45.13)^a^^,^^b^	DS cases were excluded.
Méhes et al., 1985 [19]	49 (37)	37 (25) sibling, 49 (19) controls	Leukemia	Minor BD	Siblings: 1.48 (0.57–3.82)^a^^,^^b^; Controls: 4.87 (2.04–11.60)^a^^,^^b^	Major anomalies were also reported; however there was insufficient detail to evaluate associations. The authors note two ALL cases with DS and fetal hydantoin syndrome were excluded.
Shu et al., 1988 [48]	309 (0)	618 (0)	Leukemia	Any BD	ND	The only BD noted was one DS case.
Magnani et al., 1990 [47]	142 (4)	307 (6)	ALL	Any BD	1.45 (0.40–5.24)^a^^,^^b^	DS cases were excluded.
	22 (0)	307 (6)	AML	Any BD	-	
	19 (0)	307 (6)	NHL	Any BD	-	
Zack et al., 1991 [40]	411 (31)	2,055 (99)	All leukemias	Any BD	1.6 (1.1–2.4)^b^	The authors note that only children with DS or cleft lip or palate had an increased risk of leukemia.
	411 (3)	2,055 (3)	All leukemias	Cleft lip or cleft palate	5.0 (1.0–24.8)^b^	
	411 (19)	2,055 (80)	Lymphatic leukemias	Any BD	1.2 (0.7–2.0)^b^	
	411 (3)	2,055 (3)	Lymphatic leukemias	Cleft lip or cleft palate	5.0 (1.0–24.8)^b^	
	411 (10)	2,055 (11)	Myeloid leukemias	Any BD	5.2 (2.1–12.9)^b^	
	411 (0)	2,055 (0)	Myeloid leukemias	Cleft lip or cleft palate	ND	
	411 (2)	2,055 (8)	Other or unspecified leukemias	Any BD	1.3 (0.3–6.5)^b^	
	411 (0)	2,055 (8)		Cleft lip or cleft palate	ND	
Schumacher et al., 1992 [23]	227 (61^a^)	200 (11)	ALL	Rib	6.31 (3.21–12.40)^a^^,^^b^	
	63 (7^a^)	200 (11)	NHL	Rib	2.15 (0.80–5.80)^a^^,^^b^	
	54 (4^a^)	200 (11)	HD	Rib	1.37 (0.42–4.50)^a^^,^^b^	
Mann et al., 1993 [16]	148 (11)	148 (8)	ALL	Any BD	1.41 (0.55–3.60)^a^^,^^b^	DS occurred in three leukemia cases. A variety of BDs were reported in single cases and controls but no formal analysis was done. Estimates adjusted for diagnosis age, sex, and diagnosis year. Pedigree was shown for one case with a ALL.
	23 (3)	23 (0)	Other leukemias	Any BD	ND	
	32 (4)	32 (1)	HL	Any BD	4.43 (0.47–42.02)^a^^,^^b^	
	31 (3)	31 (4)	Other lymphomas	Any BD	0.72 (0.15–3.54)^a^^,^^b^	
Savitz et al., 1994 [15]	71 (6)	212 (11)	ALL	Major	2.1 (0.8–7.8)^b^	DS exclusion not noted. Estimates adjusted for diagnosis age, sex, and diagnosis year.
	26 (2)	212 (11)	Lymphoma	Major	1.6 (0.3–7.5)^b^	
Cnattingius et al., 1995 [49]	613 (NR)	3,065 (NR)	Lymphatic leukemia	Cleft lip or palate (ICD-8 code 749)	2.0 (0.4–10.3)^b^	Authors’ note: "Among infants with congenital malformations, only those with Down's syndrome … had an increased risk of lymphatic leukemia." The authors did not report risk estimates for BDs overall.
Cnattingius et al., 1995 [41]	84 (5)	490 (18)	Myeloid leukemia	Any BD	1.4 (0.5–3.8)^b^	DS cases were excluded.
	84 (3)	490 (3)	Myeloid leukemia	Heart	5.0 (1.01–24.8)^b^	
Adami et al., 1996 [54]	168 (5)	840 (37)	NHL	Any BD (ICD-9 codes 740–759)	0.7 (0.3–1.7)^b^	DS exclusion not noted.
Altmann et al., 1998 [17]	224 (19)	NR (NR)	Leukemia	Any BD	3.7 (2.2–6.5)^b^	DS cases were not excluded except where noted. Any BD estimates adjusted for 6-month calendar period, gender, birth weight, gestational age, and maternal age.
	185 (9)	NR (NR)	ALL	Any BD	2.0 (0.96–4.1)^b^	
	32 (7)	NR (NR)	AML	Any BD	11.6 (4.6–29.4)^b^	
	32 (1)	NR (3)	AML	Non-DS chromosomal	20.3 (1.8–224)^b^	
	45 (2)	NR (NR)	Lymphoma	Any BD	2.1 (0.5–9.2)^b^	
Méhes et al., 1998 [52]	100 (82^a^)	Controls: 200 (83^a^); Siblings: 80 (69^a^)	Leukemia	Mild errors of morphogenesis (55 MEMs based on Pinsky et al.[111])	NR	DS exclusion not noted. Case and control mean MEM numbers were 1.59 and 0.69 (p<0.01); Cases and sibling mean MEM numbers were 1.59 and 1.51 (not significant).
Mertens et al., 1998 [43]	2,117 (526)	2,368 (443)	ALL	Overall (at least 1 BD)	1.43 (1.25–1.66)^a^^,^^b^	Estimates adjusted for maternal race, maternal education, and family income. After exclusion of DS cases, the significant associations between pancreatic or digestive tract abnormalities and ALL and between multiple birthmarks and both ALL and AML remained.
	2,117 (664)	2,368 (552)	ALL	Total no. of abnormalities	1.50 (1.32–1.72)^b^	
	2,117 (4)	2,368 (10)	ALL	Cleft lip or palate	0.45 (0.14–1.38)^b^	
	2,117 (5)	2,368 (7)	ALL	Club foot	0.80 (0.25–2.51)^b^	
	2,117 (161)	2,368 (136)	ALL	Large or multiple birthmarks	1.35 (1.07–1.71)^b^	
	2,117 (8)	2,368 (8)	ALL	Deafness or impaired hearing	1.12 (0.42–2.99)^b^	
	2,117 (20)	2,368 (18)	ALL	Blindness or difficulty seeing	1.25 (0.66–2.36)^b^	
	2,117 (10)	2,368 (4)	ALL	Eyes different colors or missing iris	2.81 (0.92–8.53)^b^	
	2,117 (5)	2,368 (3)	ALL	Water on the brain or hydrocephalus	1.87 (0.46–7.64)^b^	
	2,117 (4)	2,368 (3)	ALL	Spina bifida or other spinal	1.49 (0.34–6.61)^b^	
	2,117 (2)	2,368 (7)	ALL	Cerebral palsy	0.32 (0.07–1.42)^b^	
	2,117 (3)	2,368 (2)	ALL	Unusually small head or microcephaly	1.68 (0.29–9.86)^b^	
	2,117 (3)	2,368 (2)	ALL	Unequal sized limbs or hemihypertrophy	1.68 (0.29–9.86)^b^	
	2,117 (73)	2,368 (72)	ALL	Any skeletal deformity	1.14 (0.82–1.59)^b^	
	2,117 (98)	2,368 (75)	ALL	Hole in heart or other heart	1.48 (1.09–2.01)^b^	
	2,117 (29)	2,368 (13)	ALL	Pancreas or digestive tract	2.52 (1.33–4.75)^b^	
	2,117 (47)	2,368 (49)	ALL	Any other BDs	1.08 (0.72–1.61)	
	2,117 (39)	2,368 (31)	ALL	Genitourinary	1.42 (0.88–2.27)^b^	
	605 (149)	769 (115)	AML	Overall (at least 1 BD)	1.86 (1.42–2.44)^a^^,^^b^	
	605 (231)	769 (135)	AML	Total no. of abnormalities	2.90 (2.27–3.70)^b^	
	605 (5)	769 (1)	AML	Cleft lip or palate	6.40 (0.98–41.64)^b^	
	605 (1)	769 (2)	AML	Club foot	0.64 (0.06–6.88)^b^	
	605 (39)	769 (27)	AML	Large or multiple birthmarks	1.89 (1.15–3.11)^b^	
	605 (5)	769 (1)	AML	Deafness or impaired hearing	6.40 (0.98–41.64)^b^	
	605 (3)	769 (7)	AML	Blindness or difficulty seeing	0.54 (0.14–2.07)^b^	
	605 (0)	769 (0)	AML	Eyes different colors or missing iris	-	
	605 (3)	769 (2)	AML	Water on the brain or hydrocephalus	1.91 (0.33–11.14)^b^	
	605 (3)	769 (1)	AML	Spina bifida or other spinal defect	3.83 (0.47–31.45)^b^	
	605 (3)	769 (0)	AML	Unusually small head or microcephaly	ND	
	605 (2)	769 (0)	AML	Unequal sized limbs or hemihypertrophy	ND	
	605 (16)	769 (12)	AML	Any skeletal deformity	1.71 (0.81–3.62)^b^	
	605 (27)	769 (17)	AML	Hole in heart or other heart	2.07 (1.13–3.78)^b^	
	605 (6)	769 (4)	AML	Pancreas or digestive tract	1.92 (0.55–6.68)^b^	
	605 (7)	769 (11)	AML	Genitourinary	0.81 (0.31–2.09)^b^	
	605 (10)	769 (17)	AML	Other BDs	0.74 (0.34–1.63)^b^	
Infante-Rivard et al., 2001 [42]	491 (49)	491 (46)	ALL	Any BD	1.07 (0.70–1.65)^b^	DS exclusion not noted. Estimates adjusted for maternal education, paternal and maternal age.
Roganovic et al., 2002 [50]	64 (55)	64 (43)	Hematologic malignancies (HM)	Any minor	2.98 (1.24–7.17)^a^^,^^b^	DS exclusion not noted. The authors also reported data for ALL, AML, NHL and HD but did not report the overall number of cases who had at least one minor anomaly for each of these subtypes. The authors also noted “a statistically significant excess of multiple birthmarks”.
Merks et al., 2005 [26]	26 (0)	881 (54)	AML	Cervical rib	-	
	132 (16)	881 (54)	ALL	Cervical rib	2.11 (1.17–3.81)^a^^,^^b^	
	92 (5)	881 (54)	HD	Cervical rib	0.88 (0.34–2.26)^a^^,^^b^	
	106 (8)	881 (54)	NHL	Cervical rib	1.25 (0.58–2.70)^a^^,^^b^	
Podvin et al., 2006 [44]	595 (11)	5,950 (93)	Leukemia	Other malformations (not DS)	1.2 (0.6–2.2)^b^	Estimates adjusted for maternal age.
Loder et al., 2007 [25]	109^a^ (16)	200 (16)	ALL, AML, lymphoma	Abnormal rib number (normal = 24 ribs)	2.0 (1.0–4.1)^b^	Children with a known BD or DS were excluded.
Johnson et al., 2010 [46]	443 (77)	324 (40)	All infant leukemia	Any BD	1.2 (0.8–1.9)^b^	DS cases excluded. Estimates adjusted for birth year and follow-up time.
	443 (41)	324 (18)	All infant leukemia	Large or multiple birthmarks	1.3 (0.7–2.4)^b^	
	443 (9)	324 (9)	All infant leukemia	Urogenital system	0.7 (0.2–2.0)^b^	
	443 (29)	324 (15)	All infant leukemia	Other	1.4 (0.7–2.8)^b^	
	264 (47)	324 (40)	Infant ALL	Any BD	1.4 (0.8–2.3)^b^	
	264 (26)	324 (18)	Infant ALL	Large or multiple birthmarks	1.5 (0.8–3.0)^b^	
	264 (5)	324 (9)	Infant ALL	Urogenital system	0.8 (0.2–2.6)^b^	
	264 (17)	324 (15)	Infant ALL	Other	1.5 (0.7–3.3)^b^	
	172 (30)	324 (40)	Infant AML	Any BD	1.4 (0.8–2.4)^b^	
	172 (15)	324 (18)	Infant AML	Large or multiple birthmarks	1.4 (0.6–2.9)^b^	
	172 (4)	324 (9)	Infant AML	Urogenital system	0.7 (0.2–3.2)^b^	
	172 (12)	324 (15)	Infant AML	Other	1.6 (0.7–3.8)^b^	
Citak et al., 2011 [51]	109 (70)	109 (29)	HM	Minor	4.95 (2.78–8.82)^a^^,^^b^	Cases with major malformations and syndromes were excluded. Among minor anomalies, pigmented nevi and café-au-lait spots were significantly more frequent in the cases.
Durmaz et al., 2011 [20]	112 (109^a^)	200 (70^a^)	HM	Any minor	67.48 (20.7–220.3)^a^^,^^b^	Patients with known genetic syndromes were excluded. Data on 19 specific minor anomalies was also reported (not shown).
	112 (22)	200 (13)	HM	Hand	3.5 (1.69–7.29)^b^	
	112 (34)	200 (7)	HM	Feet	12.02 (5.11–28.25)^b^	
	112 (53)	200 (37)	HM	Eye	3.9 (2.36–6.62)^b^	
	112 (9)	200 (7)	HM	Nose	2.4 (0.87–6.65)^b^	
	112 (64)	200 (27)	HM	Mouth	8.5 (4.92–14.83)^b^	
	112 (49)	200 (8)	HM	Ear	18.7 (8.4–41.53)^b^	
Zierhut et al., 2011 [24]	221 (15)	1,133 (51)	Leukemias, MPD, MDS	Abnormal ribs (normal = 24 ribs)	1.55 (0.84–2.88)^b^	Estimates adjusted for sex and age.
	105 (6)	1,133 (51)	Lymphoid leukemias	Abnormal ribs	1.27 (0.51–3.11)^b^	
	78 (8)	1,133 (51)	AML	Abnormal ribs	2.29 (1.02–5.13)^b^	
	20 (0)	1,133 (51)	Chronic myeloproliferative diseases	Abnormal ribs	-	
	18 (1)	1,133 (51)	Other specified or unspecified leukemias	Abnormal ribs	1.16 (0.15–8.98)^b^	
	50 (1)	1,133 (51)	Lymphomas and reticuloendothelial neoplasms	Abnormal ribs	0.37 (0.05–2.89)^b^	
	29 (0)	1,133 (51)	HL	Abnormal ribs	-	
	12 (1)	1,133 (51)	NHL (except Burkitt's lymphoma)	Abnormal ribs	1.67 (0.21–13.41)^b^	
	9 (0)	1,133 (51)	Other specified or unspecified lymphomas	Abnormal ribs	-	
Rudant et al., 2013 [45]	764 (35)	1,681 (51)	Acute leukemia	Any BD	1.5 (1.0–2.4)^b^	DS cases excluded. Estimates adjusted for age x gender, maternal age at child's birth, parental professional category. P-values were calculated by Fisher’s exact test.
	764 (26)	1,681 (32)	Acute leukemia	Minor	1.8 (1.0–3.1)^b^	
	764 (9)	1,681 (19)	Acute leukemia	Major	1.1 (0.5–2.4)^b^	
	764 (3)	1,681 (6)	Acute leukemia	Face/tongue/mouth	0.9 (0.2–3.7)^b^	
	764 (3)	1,681 (9)	Acute leukemia	Heart	0.7 (0.2–2.8)^b^	
	764 (4)	1,681 (1)	Acute leukemia	Digestive tract	0.01<p<0.05	
	764 (7)	1,681 (10)	Acute leukemia	Urinary system	1.7 (0.6–4.7)^b^	
	764 (3)	1,681 (6)	Acute leukemia	Genital organs	1.0 (0.2–4.2)^b^	
	764 (9)	1,681 (23)	Acute leukemia	Skeleton	0.8 (0.4–1.9)^b^	
	764 (5)	1,681 (0)	Acute leukemia	Skin	0.001<p<0.01	
	764 (2)	1,681 (0)	Acute leukemia	Nervous System	NR	
	648 (28)	1,681 (51)	ALL	Any BD	1.4 (0.9–2.2)^b^	
	648 (20)	1,681 (32)	ALL	Minor	1.6 (0.9–2.8)^b^	
	648 (8)	1,681 (19)	ALL	Major	1.1 (0.5–2.5)^b^	
	648 (2)	1,681 (6)	ALL	Face/tongue/mouth	0.7 (0.1–3.5)^b^	
	648 (2)	1,681 (9)	ALL	Heart	0.5 (0.1–2.6)^b^	
	648 (4)	1,681 (1)	ALL	Digestive tract	0.01< p<0.05	
	648 (6)	1,681 (10)	ALL	Urinary system	1.6 (0.6–4.9)^b^	
	648 (2)	1,681 (6)	ALL	Genital organs	0.8 (0.1–3.9)^b^	
	648 (8)	1,681 (23)	ALL	Skeleton	0.9 (0.4–2.0)^b^	
	648 (5)	1,681 (0)	ALL	Skin	NR	
	648 (0)	1,681 (0)	ALL	Nervous System	NR	
	101 (6)	1,681 (51)	AML	Any BD	2.3 (1.0–5.7)^b^	
	101 (5)	1,681 (32)	AML	Minor	3.0 (1.1–8.2)^b^	
	101 (1)	1,681 (19)	AML	Major	NR	
	18 (0)	NR (NR)	Pro-B	Any BD	-	
	508^a^ (22)	NR (NR)	Pre-B/Common B	Any BD	1.3 (0.8–2.2)^b^	
	32 (0)	NR (NR)	B mature	Any BD	-	
	67 (5)	NR (NR)	T-cell	Any BD	2.4 (0.9–6.3)	
***Leukemias*, *myeloproliferative diseases*, *myelodysplastic diseases*, *and lymphoma cohort studies***						
Windham et al., 1985 [27]	20 (22,856)	NR (NR)	Leukemia	Any BD	2.4 (1.6–3.7)^c^	The authors note that most of the excess was due to DS cases. Estimates are age standardized.
	NR (NR)	NR (NR)	Leukemia	Any BD (Male)	2.6^c^*	
	NR (NR)	NR (NR)	Leukemia	Any BD (Female)	2.2^c^*	
	NR (NR)	NR (NR)	Leukemia (0–4 years)	Any BD (Male)	2.4^c^*	
	NR (NR)	NR (NR)	Leukemia (0–4 years)	Any BD (Female)	2.4^c^*	
	NR (NR)	NR (NR)	Leukemia (5–9 years)	Any BD (Male)	3.9^c^*	
	NR (NR)	NR (NR)	Leukemia (5–9 years)	Any BD (Female)	1.7^c^*	
Mili et al., 1993 [28]	8 (19,373)	116 (544,304)	Leukemia	Any BD	2.0 (0.8–3.9)^d^; 1.2 (0.40–2.9)^d^ DS excluded	Did not exclude any known genetic syndromes. Estimates adjusted for age. The authors also note adjusting for sex and race in some analyses.
Mili et al., 1993 [29]	3 (10,891)	102 (241,473)	Leukemia	Any BD	1.1 (0.2–3.2)^d^; 0.36 (0.009–2.0)^d^ DS excluded	Leukemia was only associated with DS. Estimates adjusted for age and sex.
Agha et al., 2005 [33]	48 (45,200)	19 (45,200)	Leukemia	Any BD	2.7 (2.1–3.6)^c^	Did not exclude any known genetic syndromes. O/E ratios are age standardized.
	NA	NA	Leukemia	Chromosomal	8.8 (5.2–14.8)^g^	
	8 (45,200)	9 (45,200)	Lymphoma	Any BD	1.0 (0.6–1.6)^c^	
	NA	NA	Lymphoma	Certain musculoskeletal deformities	3.5 (1.5–8.4)^g^	
Rankin et al., 2008 [30]	10 (NR)	218^a^ (NR)	ALL	Any BD	2.73 (1.49–5.02)^f^	The authors report that the association between leukemia and BDs was still present after excluding DS cases.
	7 (NR)	19^a^ (NR)	AML	Any BD	21.97 (12.07–39.96)^f^	
	1 (NR)	8^a^ (NR)	Other leukemia	Any BD	7.45 (1.27–43.60)^f^	
	5 (NR)	56^a^ (NR)	HL and NHL	Any BD	5.32 (2.35–12.04)^f^	
Carozza et al., 2012 [31]	84 (115,686)	742 (3,071,255^a^)	Leukemia	Any BD	1.39 (1.09–1.75)^f^	Genetic syndromes were not excluded. The authors note that excluding subjects with chromosomal anomalies resulted in a lower IRR for leukemia and total cancers but not for other cancers.
	8 (115,686)	137 (3,071,255^a^)	Lymphoma	Any BD	1.80 (0.76–3.64)^f^	
Fisher et al., 2012 [36]	29 (59,258)	NR	Leukemia	Non-chromosomal	0.96 (0.66–1.38)^e^	
	77 (6,327)	NR	Leukemia	Chromosomal	28.99 (23.07–36.42)^e^	
	33 (6,327)	NR	ALL	Chromosomal	14.95 (10.59–21.11)^e^	
	35 (6,327)	NR	AML	Chromosomal	101.22 (70.87–144.57)^e^	
	17 (59,258)	NR	Lymphoma	Non-chromosomal	2.24 (1.38–3.63)^e^	
Botto et al., 2013 [32]	13 (44,151)	45 (147,940)	Leukemia	Structural	1.19 (0.6–2.2)^f^	Children with chromosomal anomalies were excluded.
	12 (44,151)	36 (147,940)	ALL	Structural	1.38 (0.7–2.6)^f^	
	<5 (44,151)	5 (147,940)	AML	Structural	0.83 (0.1–7.1)^f^	
	0 (44,151)	<5 (147,940)	Other and unspecified	Structural	-	
	<5 (44,151)	<5 (147,940)	MDS/MPD	Structural	12.41 (1.3–119.3)^f^	
	<5 (44,151)	10 (147,940)	Lymphoma	Structural	1.65 (0.5–5.3)^f^	
	<5 (44,151)	6 (147,940)	NHL	Structural	0.69 (0.1–5.7)^f^	
	<5 (44,151)	<5 (147,940)	HL	Structural	1.03 (0.1–9.2)^f^	
	<5 (44,151)	0 (147,940)	Other, unspecified	Structural	-	
Sun et al., 2014 [38]	1 (4,484)	47 (1,547,126)	LHT (<1 year)	Nervous system (cohort entry on day of birth)	7.35 (1.01–53.3)^e^	Children with chromosomal anomalies were excluded. Estimates adjusted for calendar year and sex.
	NR (NR)	NR (NR)	LHT (<1 year)	Nervous system (cohort entry at BD diagnosis)	-	
	5 (4,484)	787 (1,547,126)	LHT (1–15 years)	Nervous system (cohort entry on day of birth)	2.12 (0.88–5.11)^e^	
	NR (NR)	NR (NR)	LHT (1–15 years)	Nervous system (cohort entry at BD diagnosis)	1.56 (0.50–4.85)^e^	
	4 (24,643)	47 (1,547,126)	LHT (<1 year)	Circulatory system (cohort entry on day of birth)	5.34 (1.92–14.83)^e^	
	NR (NR)	NR (NR)	LHT (<1 year)	Circulatory system (cohort entry at BD diagnosis)	2.36 (0.33–17.08)^e^	
	40 (4,484)	787 (1,547,126)	LHT (1–15 years)	Circulatory system (cohort entry on day of birth)	3.27 (2.38–4.49)^e^	
	NR (NR)	NR (NR)	LHT (1–15 years)	Circulatory system (cohort entry at BD diagnosis)	1.43 (0.84–2.43)^e^	
Dawson et al., 2015 [34]	13 (32,273)	291^a^ (608,726)	Leukemia MPD and MPS	N BDs	0.83 (0.48–1.44)^e^	Excluded syndromes known to be associated with cancer (e.g. DS) (N category).
	9 (32,273)	242^a^ (608,726)	ALL	N BDs	0.69 (0.36–1.34)^e^	
	1 (32,273)	36^a^ (608,726)	AML	N BDs	0.52 (0.07–3.76)^e^	
	7 (32,273)	9^a^ (608,726)	Other leukemias	N BDs	4.30 (1.23–15.09)^e^	
	3 (32,273)	85^a^ (608,726)	Lymphoma	N BDs	1.63 (0.75–3.53)^e^	
Janitz et al., 2016 [35]	18 (23,368^a^)	194^a^ (567,867)	Leukemia	Any BD (w/chromosomal)	2.3 (1.4–3.8)^e^	For lymphoma, the exact number of cases was suppressed.
	10 (22,109^a^)	194^a^ (567,867)	Leukemia	Non-chromosomal	1.4 (0.7–2.6)^e^	
	≤5 (22,109^a^)	≤39^a^ (567,867)	Lymphoma	Any BD (w/chromosomal)	2.8 (1.0–7.9)^e^	
	≤5 (22,109^a^)	≤39^a^ (567,867)	Lymphoma	Non-chromosomal	3.0 (1.1–8.4)^e^	
***Leukemias*, *myeloproliferative diseases*, *myelodysplastic diseases*, *and lymphoma case series with an external comparison group studies***						
Narod et al., 1997 [53]	6,588 (6)	NA	Leukemia	Spina bifida	0.76 (NRCT); 1.8 (BC)	Two hundred seventy-five cases with an established genetic cause were removed; authors report RRs but do not define the RR abbreviation.
	6,588 (8)	NA	Leukemia	Cardiac septal	0.54; 0.28	
	6,588 (36)	NA	Leukemia	Genitourinary	0.64; 0.56	
	6,588 (6)	NA	Leukemia	Spine and rib	0.4; 3.1*	
	2,191 (3)	NA	Lymphoma	Cardiac septal	0.61; 0.31	
	2,191 (20)	NA	Lymphoma	Genitourinary	1.1; 0.94	
	2,191 (7)	NA	Lymphoma	Spine and rib	1.3; 10.9**	
***Central nervous system and miscellaneous intracranial and intraspinal neoplasms case-control studies***						
Johnson et al., 1987 [62]	499 (NR)	998 (NR)	CNS	Any BD	NR	Genetic syndromes were not noted as excluded; The authors concluded that there was no association.
Baptiste et al., 1989 [55]	338 (14)	676 (28)	CNS	Any BD	1.00 (0.49–2.00)^h^	Does not include Neurofibromatosis Type 1 cases.
Birch et al., 1990 [56]	78 (16)	78 (7)	CNS	Any BD	2.6 (0.9–7.6)^h^	Hospital controls and children with single small birthmarks were excluded.
	78 (10)	78 (4)	CNS	Medically confirmed BDs	2.7 (0.7–10.9)^h^	
Schumacher et al., 1992 [23]	234 (64^a^)	200 (11)	Brain	Rib	6.47 (3.30–12.67)^a^^,^^b^	
Mann et al., 1993 [16]	78 (10)	78 (3)	CNS	Any BD	3.68 (0.97–13.92)^a^^,^^b^	No CNS tumor cases were noted with genetic syndromes; Four cases were reported by Birch et al. [56] but one case with a hairy mole/naevus was excluded.
Cordier et al., 1994 [61]	75 (NR)	113 (NR)	Brain	BDs (excluding minor)	NR	The authors note that the percentage of children reported with BDs (excluding minor anomalies) was higher among controls (11%) than cases (7%).
Gold et al., 1994 [64]	361 (1)	1,086 (6)	Brain	Ear/Congenital deafness	0.50 (0.06–4.17)^a^^,^^b^	
	361 (3)	1,086 (3)	Brain	Vertebral/back/curvature of the spine	3.03 (0.61–15.06)^a^^,^^b^	
	361 (3)	1,086 (6)	Brain	Eye	1.50 (0.38–6.06)^a^^,^^b^	
	361 (0)	1,086 (1)	Brain	Cleft lip or palate	-	
	361 (1)	1,086 (0)	Brain	Hydrocephalus	-	
	361 (0)	1,086 (9)	Brain	Congenital heart disease	-	
	361 (0)	1,086 (7)	Brain	Polydactyly/other digit	-	
	361 (4)	1,086 (0)	Brain	Neurofibromatosis	-	
	361 (0)	1,086 (4)	Brain	Absent/extra/deformed kidney	-	
	361 (0)	1,086 (1)	Brain	Spina bifida	-	
	361 (5)	1,086 (3)	Brain	Club foot	5.07 (1.21–21.32)^a^^,^^b^	
	361 (1)	1,086 (10)	Brain	Digestive System	0.30 (0.04–2.34)^a^^,^^b^	
	361 (2)	1,086 (2)	Brain	Other urogenital system	3.02 (0.42–21.51)^a^^,^^b^	
	361 (14)	1,086 (27)	Brain	Other/multiple	1.58 (0.82–3.05)^a^^,^^b^	
McCredie et al., 1994 [65]	82 (17)	164 (32)	Brain	Birthmarks/deformities	1.1 (0.5–2.1)^b^	Estimate adjusted for years of schooling of father.
Savitz et al., 1994 [15]	47 (3)	212 (11)	CNS	Major	1.2 (0.3–4.7)^b^	Genetic syndromes were not noted as excluded. All cases were noted to have had a CT scan but not controls.
Altmann et al., 1998 [17]	92 (9)	NR (NR)	CNS	Any BD	4.7 (2.2–10.0)^b^	DS cases were not excluded. Any BD estimates adjusted for 6-month calendar period, gender, birth weight, gestational age, and maternal age.
	92 (4)	NR (3)	CNS	Nervous system	27.8 (6.1–127)^b^	
	92 (2)	NR (11)	CNS	Eye/face/neck	16.8 (2.7–103)^b^	
	39 (3)	NR (NR)	AST	Any BD	3.5 (0.98–12.5)^b^	
Merks et al., 2005 [26]	21 (2)	881 (54)	MB	Cervical	1.61 (0.37–7.10)^a^^,^^b^	
	22 (4)	881 (54)	AST	Cervical	3.40 (1.11–10.41)^a^^,^^b^	
Loder et al., 2007 [25]	37 (13)	200 (16)	Neural (CNS)	Abnormal rib number (normal = 24 ribs)	6.23 (2.7–14.5)^b^	Children with a known BD or DS were excluded.
Mallol-Mesnard et al., 2008 [58]	209 (8)	1,681 (57)	All CNS	Any BD	1.1 (0.5–2.3)^b^	Estimates adjusted for age and sex.
	209 (6)	1,681 (38)	All CNS	Major	1.2 (0.5–2.9)^b^	
	33 (1)	1,681 (57)	EPD	Any BD	1.0 (0.1–7.2)^b^	
	100 (3)	1,681 (57)	Embryonal	Any BD	0.8 (0.2–2.6)^b^	
	100 (2)	1,681 (38)	Embryonal	Major	0.8 (0.2–3.5)^b^	
	26 (1)	1,681 (57)	AST	Any BD	1.2 (0.2–9.2)^b^	
	26 (1)	1,681 (38)	AST	Major	1.9 (0.2–14)^b^	
	45 (3)	1,681 (57)	Other gliomas	Any BD	2.0 (0.6–6.6)^b^	
	45 (3)	1,681 (38)	Other gliomas	Major	2.9 (0.9–10)^b^	
Durmaz et al., 2011 [20]	28 (9)	200 (13)	CNS	Hand	6.8 (2.57–18.01)^b^	Patients with known genetic syndromes were excluded. Data on 19 specific minor anomalies was also reported.
	28 (9)	200 (7)	CNS	Feet	13.06 (4.37–39.01)^b^	
	28 (16)	200 (37)	CNS	Eye	5.9 (2.56–13.46)^b^	
	28 (4)	200 (7)	CNS	Nose	4.6 (1.25–16.85)^b^	
	28 (24)	200 (27)	CNS	Mouth	38.4 (12.37–119.43)^b^	
	28 (11)	200 (8)	CNS	Ear	15.5 (5.50–43.80)^b^	
Partap et al., 2011 [57]	3,733 (45)	14,932 (90)	CNS	Any BDs	1.82 (1.25–2.65)^b^	Estimates adjusted for birth weight, birth order, race, Hispanic ethnicity, and maternal age.
	NR (NR)	NR (NR)	CNS (<2 years)	Any BD	1.70 (1.12–2.57)^b^	
	NR (NR)	NR (NR)	CNS (<1 year)	Any BD	2.91 (1.68–5.05)^b^	
	1,380 (NR)	14,932 (90)	LGG	Any BDs	0.71 (0.29–1.72)^b^	
	757 (NR)	14,932 (90)	HGG	Any BDs	0.64 (0.19–2.24)^b^	
	516 (NR)	14,932 (90)	MB	Any BDs	3.19 (1.29–7.87)^b^	
	402 (NR)	14,932 (90)	PNET	Any BDs	2.97 (1.21–7.28)^b^	
	187 (NR)	14,932 (90)	GCT	Any BDs	7.20 (2.10–24.63)^b^	
	292 (NR)	14,932 (90)	EPD	Any BDs	1.17 (0.13–10.61)^b^	
Zierhut et al., 2011 [24]	34 (3)	1,133 (51)	CNS and miscellaneous intracranial and intraspinal neoplasms	Abnormal ribs	2.00 (0.59–6.77)^b^	Estimates adjusted for sex and age.
Greenop et al., 2014 [60]	282 (14)	898 (38)	CBT	BD	1.17 (0.63–2.2)^a^^,^^b^	
Bailey et al., 2017 [59]	510 (19^a^)	3,102 (109^a^)	Any CBT	BD	1.0 (0.6–1.6)^b^	Estimates adjusted for sex and age.
	64 (3^a^)	3,102 (109^a^)	EPD	BD	1.4 (0.4–4.5)^b^	
	120 (5^a^)	3,102 (109^a^)	AST	BD	1.2 (0.5–2.9)^b^	
	206 (7^a^)	3,102 (109^a^)	Embryonal	BD	0.9 (0.4–1.9)^b^	
	109 (4^a^)	3,102 (109^a^)	Other gliomas	BD	1.0 (0.4–2.8)^b^	
***Central nervous system and miscellaneous intracranial and intraspinal neoplasms cohort studies***						
Windham et al., 1985 [27]	9 (22,856)	NR (NR)	Nervous system	Any BD	1.7 (0.9–3.2)^c^	Did not exclude any known genetic syndromes. Estimates are age standardized. The authors note that the RR = 34.7 was significant and that risk was limited to first two years of life.
	6 (NR)	NR (NR)	CNS tumor (malignant and benign)	CNS defect	34.7^c^* (NR)	
	NR (NR)	NR (NR)	Nervous system	Male	2.0^c^	
	NR (NR)	NR (NR)	Nervous system	Female	1.3^c^	
	NR (NR)	NR (NR)	Nervous system (0–4 years)	Male	2.6^c^*	
	NR (NR)	NR (NR)	Nervous system (0–4 years)	Female	1.3^c^	
	NR (NR)	NR (NR)	Nervous system (5–9 years)	Male	1.2^c^	
	NR (NR)	NR (NR)	Nervous system (5–9 years)	Female	1.4^c^	
Mili et al., 1993 [28]	10 (19,373)	77 (544,304)	Brain	Any BD	2.2 (1.04–4.0)^d^	Did not exclude any known genetic syndromes. Estimates adjusted for age. The authors also note adjusting for sex and race in some analyses.
Mili et al., 1993 [29]	3 (10,891)	52 (241,473)	Brain	Any BD	2.1 (0.4–6.2)^d^	Did not exclude any known genetic syndromes. Estimates adjusted for age and sex.
Agha et al., 2005 [33]	35 (45,200)	15 (45,200)	CNS	Any BD	2.5 (1.8–3.4)^c^	Did not exclude any known genetic syndromes. O/E ratio is age standardized.
	NA	NA	CNS	Other nervous system	5.7 (2.7–11.9)^g^	
Rankin et al., 2008 [30]	4 (NR)	198^a^ (NR)	Brain	Any BD	1.20 (0.45–3.24)^f^	Did not exclude any known genetic syndromes.
Carozza et al., 2012 [31]	35 (115,686)	394 (3,071,255^a^)	CNS	Any BD	1.11 (0.76–1.57)^f^	Genetic syndromes were not excluded. The authors note that excluding subjects with chromosomal anomalies resulted in a lower IRR for leukemia and total cancers but not for other cancers.
Fisher et al., 2012 [36]	34 (59,258)	NR	CNS	Non-chromosomal	1.80 (1.28–2.53)^e^	
	3 (6,327)	NR	CNS	Chromosomal	1.87 (0.60–5.79)^e^	
Botto et al., 2013 [32]	16 (44,151)	38 (147,940)	Brain	Structural	1.74 (1.0–3.1)^f^	Children with chromosomal anomalies were excluded.
	5 (44,151)	17 (147,940)	AST	Structural	1.22 (0.4–3.33)^f^	
	<5 (44,151)	13 (147,940)	MB	Structural	1.27 (0.4–3.9)^f^	
	7 (44,151)	8 (147,940)	Other brain	Structural	3.62 (1.3–10)^f^	
Sun et al., 2014 [38]	9 (4,484)	28 (1,547,126)	CNS (<1 year)	Nervous system (cohort entry on day of birth)	111.2 (52.46–235.61)^e^	Children with chromosomal anomalies were excluded. Estimates adjusted for calendar year and sex.
	NR (NR)	NR (NR)	CNS (<1 year)	Nervous system (cohort entry at BD diagnosis)	87.98 (31.06–249.20)^e^	
	17 (4,484)	330 (1,547,126)	CNS (1–15 years)	Nervous system (cohort entry on day of birth)	17.11 (10.51–27.86)^e^	
	NR (NR)	NR (NR)	CNS (1–15 years)	Nervous system (cohort entry at BD diagnosis)	13.21 (7.24–24.08)^e^	
	1 (4,484)	28 (1,547,126)	CNS (<1 year)	Circulatory system (cohort entry on day of birth)	2.24 (0.31–16.48)^e^	
	NR (NR)	NR (NR)	CNS (<1 year)	Circulatory system (cohort entry at BD diagnosis)	2.36 (0.33–17.08)^e^	
	13 (4,484)	330 (1,547,126)	CNS (1–15 years)	Circulatory system (cohort entry on day of birth)	2.56 (1.47–4.45)^e^	
	NR (NR)	NR (NR)	CNS (1–15 years)	Circulatory system (cohort entry at BD diagnosis)	1.43 (0.84–2.43)^e^	
Dawson et al., 2015 [34]	14 (32,273)	207^a^ (608,276)	CNS cancer and intracranial/spinal	N BDs	1.26 (0.74–2.17)^e^	Excluded syndromes known to be associated with cancer (e.g. DS) (N category).
	16 (32,273)	215^a^ (608,276)	CBT	N BDs	1.39 (0.84–2.31)^e^	
Janitz et al., 2016 [35]	11 (23,368)	91^a^ (567,867)	CNS	Any BD (w/chromosomal)	3.1 (1.6–5.7)^e^^†^	HRs were estimated at each age. The authors note that the proportional hazards assumption was not met for the overall association and that a continuous time interaction model was used for those noted with an ‘^†^’.
	NA	NA	CNS (<1 year)	Any BD (w/chromosomal)	17.9 (3.5–91.5)^e^	
	NA	NA	CNS (3 years)	Any BD (w/chromosomal)	3.6 (1.9–6.8)^e^	
	NA	NA	CNS (6 years)	Any BD (w/chromosomal)	1.9 (0.8–4.5)^e^	
	NA	NA	CNS (9 years)	Any BD (w/chromosomal)	1.3 (0.4–4.0)^e^	
	NA	NA	CNS (12 years)	Any BD (w/chromosomal)	1.0 (0.3–3.8)^e^	
	10 (22,109^a^)	91^a^ (567,867)	CNS	Non-chromosomal	2.9 (1.5–5.6)^e^^†^	
	NA	NA	CNS (<1 year)	Non-chromosomal	25 (4.8–130.0)^e^	
	NA	NA	CNS (3 years)	Non-chromosomal	3.4 (1.7–6.7)^e^	
	NA	NA	CNS (6 years)	Non-chromosomal	1.6 (0.6–4.1)^e^	
	NA	NA	CNS (9 years)	Non-chromosomal	1.0 (0.3–3.4)^e^	
	NA	NA	CNS (12 years)	Non-chromosomal	0.7 (0.2–3.1)^e^	
***Central nervous system and miscellaneous intracranial and intraspinal neoplasms nested case-control studies***						
Linet et al., 1996 [63]	570 (25)	2,850^a^ (129)	Brain	Any BD	1.0 (0.6–1.5)^b^	Estimates adjusted for matching variables.
	205 (8)	1,025^a^ (46)	Low grade AST	Any BD	0.9 (0.4–1.8)^b^	
	58 (3)	290^a^ (16)	High grade AST	Any BD	0.9 (0.3–3.3)^b^	
	93 (4)	465^a^ (23)	MB	Any BD	0.9 (0.3–2.6)^b^	
	54 (2)	270^a^ (15)	EPD	Any BD	0.7(0.1–2.9)^b^	
	160 (8)	800^a^ (29)	Other	Any BD	1.4 (0.6–3.1)^b^	
***Central nervous system and miscellaneous intracranial and intraspinal neoplasms case series with an external comparison group***						
Narod et al., 1997 [53]	4,698 (167)	NA	Brain/Spinal	Total	0.86; 0.56	Two hundred seventy-five cases with an established genetic cause were removed; authors report RRs but do not define the RR abbreviation. The authors note that 10 cases of hydrocephalus occurred in CNS tumor cases, which may be an effect rather than a cause of the tumor.
	4,698 (8)	NA	Brain/Spinal	Spina bifida (741)	1.4 (NRCT); 3.3** (BC)	
	4,698 (10)	NA	Brain/Spinal	Hydrocephalus (7423)	2.5*; 4.0***	
	4,698 (7)	NA	Brain/Spinal	Pyloric stenosis (7505)	1.4; 0.9	
	4,698 (6)	NA	Brain/Spinal	Skull (7560)	1.4; 1.4	
	4,698 (9)	NA	Brain/Spinal	Spine (7561)	0.98; 9.6***	
	4,698 (6)	NA	Brain/Spinal	Cardiac septal	0.57; 0.29	
	4,698 (26)	NA	Brain/Spinal	Genitourinary	0.65; 0.57	
	4,698 (12)	NA	Brain/Spinal	Spine and rib	1.0; 8.6***	
	4,698 (127)	NA	Brain/Spinal	Other	0.76; 0.46	
***Neuroblastoma and other peripheral nervous cell tumors case-control studies***						
Johnson et al., 1985 [66]	157 (3)	314 (2)	NB	Any BD	3.04 (0.50–18.38)^a^^,^^b^	The authors note that the cases and controls had diverse BDs.
Neglia et al., 1987 [67]	97(6)	388^a^(3)	NB	Physical anomalies	8.61 (2.00–43.86)^b^	After removal of 4 of the 6 anomalies identified in cases that were secondary to the tumor, the association was no longer significant.
Schumacher et al., 1992 [23]	88 (29^a^)	200 (11)	NB	Rib	8.44 (3.98–17.93)^a^^,^^b^	
Mann et al., 1993 [16]	31 (4)	31 (4)	NB	Any BD	1.0 (0.23–4.42)^a^^,^^b^	Matched pairs analysis
Altmann et al., 1998 [17]	56 (7)	NR (NR)	SNS	Any BD	7.2 (3.0–17.1)^b^	DS cases were not excluded. Any BD estimates adjusted for 6-month calendar period, gender, birth weight, gestational age, and maternal age.
	52 (7)	NR (NR)	NB	Any BD	7.9 (3.3–18.8)^b^	
	52 (2)	NR (NR)	NB	Eye/face/neck	26.6 (4.3–166)^b^	
	52 (2)	NR (NR)	NB	Gastrointestinal system	9.3 (1.9–46.1)^b^	
	52 (1)	NR (NR)	NB	Nervous system	18.3 (1.8–184)^b^	
Buck et al., 2001 [68]	130 (2)	301 (4)	NB	Any BD	1.2 (0.2–6.3)^b^	
Merks et al., 2005 [26]	61 (6)	881 (54)	NB	Cervical	1.67 (0.69–4.05)^a^^,^^b^	
Menegaux et al., 2005 [71]	538 (73)	504 (24)	NB	Any BD (ICD-10 740–759)	2.58 (1.57–4.25)^b^	The authors note that 9/11 eye anomalies reported among cases were most likely secondary to the tumor but the overall BD association remained unchanged when eye anomalies were excluded. Estimates adjusted for mother's educational level, mother's race and household income at birth.
	538 (23)	504 (3)	NB	Major	7.53 (2.23–25.5)^b^	
	538 (8)	504 (4)	NB	Minor	1.86 (1.06–3.25)^b^	
	538 (3)	504 (0)	NB	CNS (ICD-10 740–742)	ND	
	538 (11)	504 (1)	NB	Eye (743)	10.9 (1.38–85.8)^b^	
	538 (15)	504 (3)	NB	Cardiac (745–747)	4.27 (1.22–15.0)^b^	
	538 (6)	504 (2)	NB	Face (749–7502)	3.40 (0.68–17.1)^b^	
	538 (7)	504 (1)	NB	Digestive system (7505–751)	5.98 (0.7–50.8)^b^	
	538 (16)	504 (3)	NB	Genital and urinary (752–753)	5.84 (1.67–20.4)^b^	
	538 (9)	504 (1)	NB	Genital (752)	10.2 (1.27–82.6)^b^	
	538 (8)	504 (2)	NB	Urinary (753)	4.49 (0.93–21.7)^b^	
	538 (10)	504 (6)	NB	Musculoskeletal (754–756)	1.55 (0.55–4.35)^b^	
	538 (19)	538 (14)	NB	Other (757–759)	1.21 (0.59–2.49)^b^	
Chow et al., 2007 [70]	240 (23)	2,400 (116)	NB	Any BD	2.11 (1.32–3.40)^b^	Major anomalies are based on Cordier et al. [61], excluding 2 individuals with chromosomal anomalies. Estimates adjusted for birth year.
	240 (9)	2,400 (14)	NB	Major only	6.86 (2.92–16.08)^b^	
	240 (5)	2,400 (9)	NB	Cardiac only	5.84 (1.93–17.66)^b^	
	240 (2)	2,400 (25)	NB	Urogenital only	0.85 (0.20–3.62)^b^	
Urayama et al., 2007 [73]	508 (33)	1,015 (39)	NB	Any BD	1.77 (1.09–2.88)^b^	The OR = 1.77 is unadjusted. Other estimates adjusted for race/ethnicity, gestational age/ birth weight index, percentage of the adult population with a college degree, initiation of prenatal care, type of delivery and maternal pregnancy history.
	NR	NR	NB	Any BD	1.53 (0.87–2.68)^b^	
	228 (NR)	NR	NB (<1 year)	Any BD	1.57 (0.76–3.27)^b^	
	102 (NR)	NR	NB (1–4 years)	Any BD	1.04 (0.41–2.68)^b^	
Munzer et al., 2008 [69]	191 (12)	1,681 (57)	NB	Any BD	2.2 (1.1–4.5)^b^	Estimates adjusted for age and gender.
	191 (2)	1,681 (19)	NB	Minor BD	1.7 (0.4–7.7)^b^	
	191 (10)	1,681 (38)	NB	Major BD	2.4 (1.1–5.4)^b^	
	191 (0)	1,681 (5)	NB	Heart	-	
	191 (1)	1,681 (2)	NB	Digestive	4.6 (0.3–69)^b^	
	191 (4)	1,681 (10)	NB	Urinary	4.4 (1.1–17)^b^	
	191 (1)	1,681 (6)	NB	Genital organs	1.7 (0.2–15.0)^b^	
	191 (5)	1,681 (22)	NB	Skeleton	3.3 (1.1–9.6)^b^	
	191 (14)	1,681 (2)	NB	Others	0.7 (0.1–5.7)^b^	
	74 (7)	187 (2)	NB (<1 year)	Any BD	16.8 (3.1–90)^b^	
	74 (7)	187 (1)	NB (<1 year)	Major BD	31.3 (3.6–272)^b^	
	74 (0)	187 (1)	NB (<1 year)	Minor BD	-	
	117 (5)	1,494 (55)	NB (≥ 1 year)	Any BD	1.0 (0.3–2.9)^b^	
	117 (3)	1,494 (37)	NB (≥ 1 year)	Major BD	1.9 (0.4–8.6)^b^^b^	
	117 (2)	1,494 (18)	NB (≥ 1 year)	Minor BD	0.7 (0.2–3.0)^b^	
Zierhut et al., 2011 [24]	31 (2)	1,133 (51)	NB and other peripheral nervous cell tumors	Abnormal ribs	1.48 (0.34–6.40)^b^	Estimates adjusted for sex and age.
Parodi et al., 2014 [72]	153 (7)	1,044 (14)	NB	Any BD	4.9 (1.8–13.6)^b^	Estimates adjusted for gender, age, area of residence, maternal age, and maternal level of education.
Rios et al., 2016 [74]	357 (13)	1783 (47)	NB	Any BD	1.6 (0.8–3.0)^b^	Estimates adjusted for age and sex, birth order, maternal age, urban status of the area of residence and study
	357 (10)	1783 (43)	NB	1 BD	1.3 (0.6–2.6)^b^	
	357 (3)	1783 (4)	NB	≥2 BD	6.3 (1.3–29)^b^	
	188 (9)	544 (9)	NB (<18 months)	Any BD	3.6 (1.3–8.9)^b^	
	188 (8)	544 (9)	NB (<18 months)	1 BD	2.9 (1.1–7.9)^b^	
	188 (1)	544 (0)	NB (<18 months)	≥2 BD	-	
	169 (4)	1239 (38)	NB (≥18 months)	Any BD	0.8 (0.3–2.3)^b^	
	169 (2)	1239 (34)	NB (≥18 months)	1 BD	0.4 (1.1–1.8)^b^	
	169 (2)	1239 (4)	NB (≥18 months)	≥2 BD	4.1 (0.7–23.3)^b^	
	357 (1)	1783 (13)	NB	Cardiovascular	-	
	357 (1)	1783 (1)	NB	Digestive	-	
	357 (6)	1783 (15)	NB	Genitourinary	2.2 (0.8–6.0)b	
	357 (3)	1783 (14)	NB	Skeleton	-	
	357 (1)	1783 (0)	NB	CNS	-	
	357 (1)	1783 (4)	NB	Head and neck	-	
***Neuroblastoma and other peripheral nervous cell tumors cohort studies***						
Windham et al.,1985 [27]	2 (22,856)	NR (NR)	NB	Any BD	1.1 (0.4–3.4)^c^	Did not exclude any known genetic syndromes. Estimates are age standardized.
	NR (NR)	NR (NR)	NB	Any BD (Male)	1.1^c^	
	NR (NR)	NR (NR)	NB	Any BD (Female)	1.3^c^	
	NR (NR)	NR (NR)	NB (0–4 years)	Any BD (Male)	-	
	NR (NR)	NR (NR)	NB (0–4 years)	Any BD (Female)	1.3^c^	
	NR (NR)	NR (NR)	NB (5–9 years)	Any BD (Male)	6.3^c^	
	NR (NR)	NR (NR)	NB (5–9 years)	Any BD (Female)	-	
Mili et al., 1993 [28]	4 (19,373)	5 (544,304)	NB	Any BD	20.3 (5.5–52.1)^d^	Did not exclude any known genetic syndromes. Estimates adjusted for age. The authors also note adjusting for sex and race in some analyses.
Mili et al., 1993 [29]	2 (10,891)	32 (241,473)	NB	Any BD	2.2 (0.3–7.9)^d^	Did not exclude any known genetic syndromes. Estimates adjusted for age and sex.
Agha et al., 2005 [33]	14 (45,200)	7 (45,200)	SNS	Any BD	2.2 (1.4–3.4)^c^	Did not exclude any known genetic syndromes. O/E ratio is age standardized
	NA	NA	SNS	Other anomalies of the digestive system	5.6 (2.1–14.8)^g^	
Rankin et al., 2008 [30]	3 (NR)	77^a^ (NR)	NB	Any BD	2.32 (0.76–7.12)^f^	Did not exclude any known genetic syndromes.
Bjørge et al., 2008 [75]	8 (178^a^)	NR	NB	Any BD	2.3 (1.2–4.8)^e^	
	5 (83^a^)	NR	Adrenal medulla NB	Any BD	3.2 (1.3–7.8)^e^	
	3 (95^a^)	NR	Non-adrenal medulla NB	Any BD	1.6 (0.5–5.1)^e^	
Carozza et al., 2012 [31]	31 (115,686)	274 (3,071,225^a^)	NB	Any BD	1.43 (0.94–2.10)^f^	Genetic syndromes were not excluded. Excluding subjects with chromosomal anomalies resulted in a lower IRR for leukemia and total cancers but not for other cancers.
Fisher et al., 2012 [36]	15 (59,258)	NR	NB	Non-chromosomal	2.85 (1.69–4.78)^e^	
	1 (6,327)	NR	NB	Chromosomal	2.08 (0.29–14.82)^e^	
Botto et al., 2013 [32]	11 (44,151)	17 (147,940)	NB spectrum	Structural	2.68 (1.3–5.7)^f^	Children with chromosomal anomalies were excluded.
	8 (44,151)	15 (147,940)	NB	Structural	2.21 (0.9–5.2)^f^	
	<5 (44,151)	<5 (147,940)	Other peripheral nervous system tumor	Structural	6.20 (1–37.1)^f^	
Dawson et al., 2015 [34]	5 (32,273)	66^a^ (608,726)	NB/peripheral nervous system tumors	N BDs	1.41 (0.57–3.49)^e^	Excluded syndromes known to be associated with cancer (e.g. DS) (N category).
***Neuroblastoma and other peripheral nervous cell tumors case-cohort studies***						
Johnson et al., 2008 [76]	2 (145^a^)	122 (8,217^a^)	NB	Any BD	Too few cases to fit model	Fisher’s exact test p-value = 0.92.
***Neuroblastoma and other peripheral nervous cell tumors case series with an external comparison group studies***						
Narod et al., 1997 [53]	1,208 (71)	NA	NB	Total	1.4** (NRCT); 0.93 (BC)	Two hundred seventy-five cases with an established genetic cause were removed; authors report RRs but do not define the RR abbreviation.
	1,208 (5)	NA	NB	Ear, head, and neck (ICD-9 code 744)	3.3*; 2.2	
	1,208 (2)	NA	NB	Branchial cleft (7444)	8.3*; 9.5*	
	1,208 (3)	NA	NB	Septal defects (745)	1.1; 0.57	
	1,208 (2)	NA	NB	Tetralogy of Fallot (7452)	8.3*; 3.8	
	1,208 (2)	NA	NB	Pulmonary valve	5.5; 3.9	
	1,208 (2)	NA	NB	Other aorta	6.7; 118***	
	1,208 (3)	NA	NB	Cleft lip or palate	2.9; 1.3	
	1,208 (9)	NA	NB	Gastrointestinal (750–51)	3.1**; 2.4*	
	1,208 (4)	NA	NB	Pyloric stenosis (7505)	3.1*; 2.0	
	1,208 (2)	NA	NB	Imperforate anus (7512)	3.7; 4.7	
	1,208 (15)	NA	NB	Genitourinary (752–53)	1.5; 1.3	
	1,208 (20)	NA	NB	Musculoskeletal (754–56)	3.0***; 1.4	
	1,208 (8)	NA	NB	Foot deformity (7545–47)	3.3**; 1.4	
	1,208 (4)	NA	NB	Spinal (7561)	1.7; 16.7***	
	1,208 (4)	NA	NB	Cardiac septal	1.5; 0.76	
	1,208 (5)	NA	NB	Spine and rib	1.7; 14.3***	
	1,208 (12)	NA	NB	Other	0.48; 0.33	
	1,208 (16)	NA	NB and other sympathetic	Genitourinary	1.5; 1.3	
***Retinoblastoma and eye tumor case-control studies***						
Mann et al., 1993 [16]	6 (0)	6 (1)	RB	Any BD	-	Not significant (Fisher ‘s test)
Altmann et al., 1998 [17]	26 (5)	NR (NR)	RB	Any BD	15.0 (5.1–44.2)^b^	DS cases were not excluded. Any BD estimates adjusted for 6-month calendar period, gender, birth weight, gestational age and maternal age.
	26 (2)	NR (NR)	RB	All chromosomal	54.8 (7.7–391)^b^	
***Retinoblastoma and eye tumor cohort studies***						
Windham et al., 1985 [27]	2 (22,856)	NR (NR)	Eye	Any BD	2.3 (0.6–8.7)^c^	Did not exclude any known genetic syndromes. Estimates are age standardized.
	NR (NR)	NR (NR)	Eye	Any BD (Male)	2.4^c^	
	NR (NR)	NR (NR)	Eye	Any BD (Female)	2.2^c^	
	NR (NR)	NR (NR)	Eye (0–4 years)	Any BD (Male)	2.5^c^*	
	NR (NR)	NR (NR)	Eye (0–4 years)	Any BD (Female)	2.2^c^	
	NR (NR)	NR (NR)	Eye (5–9 years)	Any BD (Male)	-	
	NR (NR)	NR (NR)	Eye (5–9 years)	Any BD (Female)	-	
Mili et al., 1993 [28]	4 (19,373)	22 (544,304)	RB	Any BD	4.7 (1.3–12.1)^d^	Did not exclude any known genetic syndromes. Estimates adjusted for age. The authors also note adjusting for sex and race in some analyses.
Agha et al., 2005 [33]	4 (45,200)	2 (45,200)	RB	Any BD	2.2 (0.9–5.1)^c^	Did not exclude any known genetic syndromes. O/E ratio is age standardized.
	NA	NA	RB	Chromosomal	10.8 (1.4–71.1)^g^	
	NA	NA	RB	Anomalies of the eye	14.3 (2.0–99.1)^g^	
Rankin et al., 2008 [30]	2 (NR)	32^a^ (NR)	RB	Any BD	3.73 (0.99–14.10)^f^	Did not exclude any known genetic syndromes.
Carozza et al., 2012 [31]	13 (115,686)	125 (3,071,255^a^)	RB	Any BD	2.34 (1.21–4.16)^f^	Genetic syndromes were not excluded. The authors note that excluding subjects with chromosomal anomalies resulted in a lower IRR for leukemia and total cancers but not for other cancers.
Botto et al., 2013 [32]	6 (44,151)	7 (147,940)	RB	Structural	3.54 (1.2–10.5)^f^	Children with chromosomal anomalies were excluded.
Dawson et al., 2015 [34]	0 (32,273)	35 (608,726)	RB	N BDs	0^e^	Excluded syndromes known to be associated with cancer (e.g. DS) (N category).
***Retinoblastoma and eye tumor case series with an external comparison group studies***						
Narod et al., 1997 [53]	549 (36)	NA	RB	Total	1.6 (NRCT); 1.0 (BC)	Two hundred seventy-five cases with an established genetic cause were removed; authors report RRs but do not define the RR abbreviation.
	549 (2)	NA	RB	Cataract (743)	12.5*; 16.6*	
	549 (5)	NA	RB	Ventricular septal (7454)	6.1*; 4.5*	
	549 (3)	NA	RB	Other heart (746)	3.5; 2.6	
	549 (5)	NA	RB	Gastrointestinal (750–751)	3.8*; 1.8	
	549 (5)	NA	RB	Genitourinary (752–53)	1.1; 0.88	
	549 (8)	NA	RB	Musculoskeletal (754)	2.7*; 1.3	
	549 (5)	NA	RB	Cardiac septal	4.1*; 2.1	
	549 (5)	NA	RB	Genitourinary	1.1; 0.94	
	549 (0)	NA	RB	Spine and rib	0; 0	
	549 (8)	NA	RB	Other	0.67; 0.46	
***Renal tumors case-control studies***						
Wilkins et al., 1984 [77]	105 (NR)	210^a^ (NR)	WT	Any BD	NR	The authors noted that the BD frequency among cases and controls were similar.
Bunin et al., 1987 [78]	88 (10)	88 (NR)	WT	Wilms'-associated	1.5 (0.3–8.3)^b^	
	88 (13)	88 (NR)	WT	Non-Wilms'-associated	4.3 (1.0–27.6)^b^	
Kajtár et al., 1990 [79]	34 (13)	148 (41)	WT	Total vertebral	1.62 (0.74–3.52)^a^^,^^b^	
	34 (5)	148 (5)	WT	Spina bifida occulta affecting at least two vertebral arches	4.93 (1.34–18.14)^a^^,^^b^	
Schumacher et al., 1992 [23]	68 (16^a^)	200 (11)	WT	Rib	5.29 (2.31–12.09)^a^^,^^b^	
Mann et al., 1993 [16]	32 (5)	32 (1)	WT	Any BD	5.74 (0.63–52.24)^a^^,^^b^	Not significant (Fisher’s test)
Altmann et al., 1998 [17]	42 (4)	NR (NR)	Renal	Any BD	4.6 (1.6–13.8)^b^	DS cases were not excluded. Any BD estimates adjusted for 6-month calendar period, gender, birth weight, gestational age, and maternal age.
	40 (4)	NR (NR)	WT	Any BD	4.9 (1.6–14.5)^b^	
	40 (1)	NR (3)	WT	Eye/face/neck	18.9 (1.9–190)^b^	
	40 (1)	NR (5)	WT	All chromosomal	15.7 (1.7–153)^b^	
Méhes et al., 2003 [80]	50 (9)	180 (8)	WT	Spina bifida occulta	4.72 (1.72–12.98)^a^^,^^b^	
	50 (16)	180 (46)	WT	Total vertebral	1.37 (0.69–2.71)^a^^,^^b^	
	39 (14)	114 (20)	WT	Cutaneous signs of spinal dysraphism	2.63 (1.17–5.93)^a^^,^^b^	
Merks et al., 2005 [26]	133 (13)	881 (54)	WT	Cervical	1.66 (0.88–3.13)^a^^,^^b^	
Zierhut et al., 2011 [24]	20 (3)	1,133 (51)	Renal	Abnormal ribs	3.73 (1.05–13.22)^b^	Estimates adjusted for sex and age.
***Renal tumors cohort studies***						
Windham et al., 1985 [27]	4 (22,856)	NR (NR)	Renal	Any BD	2.2 (0.9–5.7)^c^	Did not exclude any known genetic syndromes. Estimates are age standardized.
	NR (NR)	NR (NR)	Renal	Male	-	
	NR (NR)	NR (NR)	Renal	Female	4.8^c^*	
	NR (NR)	NR (NR)	Renal (0–4 years)	Male	^-^	
	NR (NR)	NR (NR)	Renal (0–4 years)	Female	4.3^c^*	
	NR (NR)	NR (NR)	Renal (5–9 years)	Male	-	
	NR (NR)	NR (NR)	Renal (5–9 years)	Female	6.7^c^	
Mili et al., 1993 [28]	4 (19,373)	38 (544,304)	WT	Any BD	2.8 (0.7–7.1)^d^	Did not exclude any known genetic syndromes. Estimates adjusted for age. The authors also note adjusting for sex and race in some analyses. Minor defects were included only if they were associated with at least one major defect.
Agha et al., 2005 [33]	7 (45,200)	3 (45,200)	Renal	Any BD	1.5 (0.8–2.7)^c^	Did not exclude any known genetic syndromes.
Rankin et al., 2008 [30]	1 (NR)	59^a^ (NR)	WT	Any BD	1.01 (0.14–7.29)^f^	Did not exclude any known genetic syndromes.
Carozza et al., 2012 [31]	14 (115,686)	172 (3,071,255^a^)	Renal	Any BD	1.11 (0.59–1.91)^f^	Genetic syndromes were not excluded. Excluding subjects with chromosomal anomalies resulted in a lower IRR for leukemia and total cancers but not for other cancers.
Fisher et al., 2012 [36]	6 (59,258)	NR	WT	Non-chromosomal	1.45 (0.65–3.26)^e^	
	5 (6,327)	NR	WT	Chromosomal	13.43 (5.54–32.55)^e^	
Botto et al., 2013 [32]	<5 (44,151)	16 (147,940)	Kidney	Structural	1.03 (0.3–3.1)^f^	Children with chromosomal anomalies were excluded.
	<5 (44,151)	15 (147,940)	WT	Structural	1.10 (0.4–3.3)^f^	
	0 (44,151)	<5 (147,940)	Other kidney	Structural	0	
Dawson et al., 2015 [34]	6 (32,273)	55 (608,726)	Renal	N BDs	2.02 (0.87–4.69)^e^	Excluded syndromes known to be associated with cancer (e.g. DS) (N category).
***Renal tumors case-cohort studies***						
Puumala et al., 2008 [82]	7 (138^a^)	124 (8,752^a^)	WT	Any BD	3.20 (1.37–7.47)^e^	Estimate adjusted for sex and birth year.
***Renal tumors nested case-control studies***						
Lindblad et al., 1992 [81]	110 (NR)	550 (NR)	WT	Any BD	NR	The authors note that reported BD associations were not confirmed.
***Renal tumors case series with an external comparison group studies***						
Breslow et al., 1982 [83]	1,905^a^ (NR)	CPP: 53,257^a^ (NR); CDC Atlanta: 280,714^a^ (NR)	WT	CNS	1.12 (CPP); 2.47 (CDC)^a^^,^^c^	The ratio of the rates in NWTS versus the CPP and Atlanta are represented.
			WT	Aniridia (743.45)	840; 420^a^^,^^c^	
			WT	Eye, excluding aniridia cases (743)	0.93–1.48; 4.11^a^^,^^c^	
			WT	Ear, face, and neck (744)	0.16–4.2, 0.54^a^^,^^c^	
			WT	Cardiopulmonary system (745–8)	1.49; 1.48^a^^,^^c^	
			WT	Cleft palate-lip (749)	0.4; 0.42^a^^,^^c^	
			WT	Upper alimentary tract-digestive system (750–1)	0.92–1.19; 1.94^a^^,^^c^	
			WT	Cryptorchidism (752.5)	0.6–2.33; 1.5^a^^,^^c^	
			WT	Hypospadias (752.6)	2.05; 3.63^a^^,^^c^	
			WT	Genital (752, excluding 752.5 and 752.6)	0.6–2.33; 1.5^a^^,^^c^	
			WT	Fused kidney (753.3)	0.6–2.33; 1.5^a^^,^^c^	
			WT	Double-collecting system (753.3)	152; ND^a^^,^^c^	
			WT	Urinary system excluding fused kidney, double-collecting system (753)	2.21; 4.29^a^^,^^c^	
			WT	Clubfoot (754.5–754.7)	0.16–0.56; 0.95^a^^,^^c^	
			WT	Musculoskeletal system (754–6, excluding 754.5–754.7)	0.81–1.34; 2.07^a^^,^^c^	
			WT	Hair skin and nails (757)	0.48; 7.74^a^^,^^c^	
			WT	Chromosome defects, DS (758)	0.45; ND^a^^,^^c^	
			WT	Accessory spleen (759.0)	31.5; ND^a^^,^^c^	
			WT	Hemihypertrophy (759.8)	823.33; ND^a^^,^^c^	
Narod et al., 1997 [53]	1,148 (106)	NA	WT	Total	2.2***(NRCT); 1.5***(BC)	Two hundred seventy-five cases with an established genetic cause were removed; authors report RRs but do not define the RR abbreviation.
	1,148 (3)	NA	WT	Spina bifida (741)	1.4; 5.0*	
	1,148 (2)	NA	WT	Microcephaly (7421)	3.2; 5.7	
	1,148 (2)	NA	WT	Transposition of the great vessels (7451)	11.8*; 4.4	
	1,148 (7)	NA	WT	Ventricular septal (7454)	4.1**; 3.0*	
	1,148 (2)	NA	WT	Congenital aortic stenosis (7463)	4.3; 15.4*	
	1,148 (3)	NA	WT	Pyloric stenosis (7505)	2.5; 1.6	
	1,148 (2)	NA	WT	Ovary (7520)	18.2*; 25.0**	
	1,148 (2)	NA	WT	Uterus (7523)	11.8**; 100***	
	1,148 (8)	NA	WT	Cryptorchidism (7525)	2.2; 2.7*	
	1,148 (5)	NA	WT	Hypospadias (7526)	2.9; 1.9	
	1,148 (14)	NA	WT	Urinary system (753)	3.7***; 58.3***	
	1,148 (5)	NA	WT	Congenital hip dislocation (7543)	2.5; 1.4	
	1,148 (3)	NA	WT	Lower limb (7556)	2.1; 10.3**	
	1,148 (2)	NA	WT	Skull and facial bones (7560)	1.9; 2.0	
	1,148 (3)	NA	WT	Spinal (7561)	1.3; 13.0**	
	1,148 (2)	NA	WT	Chondrodystrophy (7564)	8.7*; 7.4	
	1,148 (3)	NA	WT	Spina bifida	2.2; 5.0*	
	1,148 (10)	NA	WT	Cardiac septal	4.1***; 2.0	
	1,148 (29)	NA	WT	Genitourinary	2.9***; 2.6***	
	1,148 (4)	NA	WT	Spine and rib	1.4; 12.1***	
	1,148 (41)	NA	WT	Other	1.6**; 0.74	
	NR (2)	NA	Renal carcinoma	Spine and rib	37**; 339***	
***Hepatic tumors case-control studies***						
Mann et al., 1993 [16]	6 (2)	6 (0)	HB	Any BD	-	Not significant (Fisher’s exact test)
Altmann et al., 1998 [17]	7 (1)	NR (NR)	Hepatic	Any BD	9.3 (0.9–94.2)	DS cases were not excluded. Estimates adjusted for 6-month calendar period, gender, birth weight, gestational age, and maternal age.
Venkatramani et al., 2014 [84]	COG:387 (1); UPDB:29 (0)	387 (1); 290 (0)	HB	Cleft lip or palate	COG: 1.05 (0.07–16.9)^b^; UPDB: -	Excluded children with Beckwith-Wiedemann Syndrome and familial adenomatous polyposis. COG estimates adjusted for sex; UPDB estimates adjusted for birth weight, maternal age, and maternal education.
	387 (4); 29 (0)	387 (2); 290 (0)	HB	Spina bifida or other spinal	2.12 (0.39–11.7)^b^; -	
	387 (37); 29 (0)	387 (30); 290 (1)	HB	Large/multiple birth marks	1.33 (0.81–2.21)^b^; -	
	387 (1); 29 (0)	387 (0); 290 (0)	HB	Trisomy 18	-; -	
	387 (2); 29 (0)	387 (1); 290 (0)	HB	Other genetic conditions	2.11 (0.19–23.4)^b^; -	
	387 (0); 29 (0)	387 (1); 290 (1)	HB	Down syndrome	-	
	387 (1); 29 (1)	387 (0); 290 (1)	HB	Rib	-; 4.3 (0.23–80.0)^b^	
	387 (3); 29 (0)	387 (0); 290 (0)	HB	Small head/microcephaly	-; -	
	387 (18); 29 (3)	387 (6); 290 (2)	HB	Kidney, bladder, sex organ	4.75 (1.74–13.0)^b^; 12.0 (1.61–89.6)^b^	
	387 (4); 29 (0)	387 (2); 290 (1)	HB	Hypospadias	2.1 (0.38–11.4)^b^; <0.001 (NA)^b^	
	387 (12); 29 (2)	387 (3); 290 (0)	HB	Any kidney/bladder	4.3 (1.2–15.3)^b^; >99 (NA)^b^	
	387 (6); 29 (1)	387 (5); 290 (1)	HB	Sex organ	1.24 (0.37–4.1)^b^; 25.7 (1.63, >99)^b^	
	NR; 29 (2)	NR; 290 (2)	HB	Heart or pulmonary	-; 7.4 (0.87–62.7)^b^	
***Hepatic tumors cohort studies***						
Agha et al., 2005 [33]	3 (45,200)	0 (45,200)	Hepatic	Any BD	-	Did not exclude any known genetic syndromes. O/E ratios are age standardized.
	NA	NA	Hepatic	Other anomalies of the digestive system	11.7 (2.9–46.7)^g^	
	NA	NA	Hepatic	Chromosomal	8.0 (1.1–56.8)^g^	
Carozza et al., 2012 [31]	16 (115,686)	45 (3,071,255^a^)	Liver	Any BD	1.00 (0.52–1.83)^f^	Genetic syndromes were not excluded. The authors note that excluding subjects with chromosomal anomalies resulted in a lower IRR for leukemia and total cancers but not for other cancers.
Botto et al., 2013 [32]	8 (44,151)	<5 (147,940)	Liver	Structural	16.54 (3.5–77.9)^f^	Children with chromosomal anomalies were excluded.
	7 (44,151)	<5 (147,940)	HB	Structural	14.47 (3.0–69.7)^f^	
	<5 (44,151)	0 (147,940)	Other liver	Structural	-	
Dawson et al., 2015 [34]	4 (32,273)	10^a^ (608,726)	Hepatic	N BDs	7.54 (2.36–24.03)^e^	Excluded syndromes known to be associated with cancer (e.g. DS) (N category).
Janitz et al., 2016 [35]	≤5 (23,368)	<9^a^ (567,867)	Hepatic	Any BD (w/chromosomal)	25.0 (7.2–86.2)^e^	The exact number of hepatic tumor cases was suppressed.
	≤5 (22,109^a^)	<9^a^ (567,867)	Hepatic	Non-chromosomal	NR	
***Hepatic tumors case-cohort studies***						
Spector et al., 2008 [85]	5 (36^a^)	112 (7,788^a^)	HB	Any BD	5.87 (1.88–18.3)^e^	Adjusted for birth weight, birth year, and sex.
***Hepatic tumors case series with an external comparison group studies***						
Narod et al., 1997 [53]	NR (2)	NA	HB	Spina bifida	2.0 (NRCT); 4.7 (BC)	Two hundred seventy-five cases with an established genetic cause were removed; authors report RRs but do not define the RR abbreviation.
	165 (3)	NA	Liver	Genitourinary	2.1; 1.9	
	165 (0)	NA	Liver	Spine and Rib	0; 0	
***Malignant bone tumors case-control studies***						
Schumacher et al., 1992 [23]	55 (3^a^)	200 (11)	OS	Rib	0.99 (0.27–3.68)^a^^,^^b^	
	35 (6^a^)	200 (11)	ES	Rib	3.55 (1.22–10.35)^a^^,^^b^	
Mann et al., 1993 [16]	30 (3)	30 (2)	Bone	Any BD	1.56 (0.24–10.05)^a^^,^^b^	
Gelberg et al., 1997 [12]	130 (NR)	130 (NR)	OS	Any BD	NR	Age range 0–24 years. No significant associations were reported.
Altmann et al., 1998 [17]	8 (2)	NR (NR)	Bone	Any BD	23.2 (3.8–143)^b^	DS cases were not excluded. Any BD estimates adjusted for 6-month calendar period, gender, birth weight, gestational age, and maternal age.
	8 (1)	NR (5)	Bone	All chromosomal	28.6 (2.9–280)^b^	
Merks et al., 2005 [26]	48 (3)	881 (54)	OS	Cervical	1.02 (0.31–3.39)^a^^,^^b^	
	39 (3)	881 (54)	ES	Cervical	1.28 (0.38–4.28)^a^^,^^b^	
Durmaz et al., 2011[20]	23 (20^a^)	200 (70^a^)	OS	Any Minor	12.4 (3.6–42.1)^a^^,^^b^	Patients with known genetic syndromes were excluded. Data on 19 specific minor anomalies was also reported.
Zierhut et al., 2011 [24]	39 (2)	1,133 (51)	Bone	Abnormal ribs	0.95 (0.21–4.31)^b^	
	21 (2)	1,133 (51)	OS	Abnormal ribs	1.81 (0.38–8.53)^b^	
	15 (0)	1,133 (51)	ES and related sarcomas of bone	Abnormal ribs	-	
	3 (0)	1,133 (51)	Other specified and unspecified malignant bone	Abnormal ribs	-	
***Malignant bone tumors cohort studies***						
Agha et al., 2005 [33]	6 (45,200)	5 (45,200)	Bone	Any BD	1.3 (0.7–2.4)^c^	Did not exclude any known genetic syndromes. O/E ratio is age standardized.
	NA	NA	Bone	Other musculoskeletal	4.3 (1.1–17.3)^g^	
Carozza et al., 2012 [31]	1 (115,686)	19 (3,071,255^a^)	Bone	Any BD	0.63 (0.02–3.99)^f^	Genetic syndromes were not excluded. The authors note that excluding subjects with chromosomal anomalies resulted in a lower IRR for leukemia and total cancers but not for other cancers.
Botto et al., 2013 [32]	0 (44,151)	<5 (147,940)	OS	Structural	-	Children with chromosomal anomalies were excluded.
	<5 (44,151)	<5 (147,940)	ES	Structural	2.07 (0.2–22.8)^f^	
Dawson et al., 2015 [34]	2 (32,273)	32^a^ (608,726)	Bone	N BDs	1.19 (0.29–4.96)^e^	Excluded syndromes known to be associated with cancer (e.g. DS) (N category).
***Malignant bone tumors case series with an external comparison group studies***						
Narod et al., 1997 [53]	NR (1)	NA	Bone	Spina bifida	20.0 (NRCT); 50.0* (BC)	Two hundred seventy-five cases with an established genetic cause were removed; authors report RRs but do not define the RR abbreviation.
	396 (26)	NA	ES	Total	1.58; 1.0	
	396 (2)	NA	ES	Spina bifida (741)	4.3; 9.5*	
	396 (2)	NA	ES	Cataracts (7433)	14.3*; 23.0**	
	396 (2)	NA	ES	Orbit (7436)	8.3; 13.3*	
	396 (5)	NA	ES	Genitourinary (752–3)	1.5; 1.2	
	396 (10)	NA	ES	Musculoskeletal (754–6)	2.0; 1.4	
	396 (2)	NA	ES	Spinal (7561)	2.6; 25.3**	
	396 (2)	NA	ES	Osteodystrophy (7565)	33.9**; 41.7**	
	396 (5)	NA	ES	Other	0.70; 0.37	
	396 (1)	NA	ES	Cardiac septal	1.1; 0.58	
	396 (5)	NA	ES	Genitourinary	1.5; 1.3	
	549 (1)	NA	OS	Genitourinary	0.21; 0.19	
	NR (6)	NA	Bone	Spine and rib	2.4; 22.2***	
***Soft tissue and other extraosseous sarcomas case-control studies***						
Schumacher et al., 1992 [23]	98 (24^a^)	200 (11)	STS	Rib	5.57 (2.60–11.95)^a^^,^^b^	
Mann et al., 1993 [16]	43 (6)	43 (1)	STS	Any BD	6.81 (0.78–59.22)^a^^,^^b^	
Savitz et al., 1994 [15]	26 (3)	212 (11)	STS	Major	2.4 (0.6–9.4)^b^	
Yang et al., 1995 [86]	249^a^ (56)	302 (55)	RMS	Any BD	1.3 (0.85–2.02)^b^	
	249^a^ (15)	302 (8)	RMS	Major	2.36 (0.92–6.18)^b^	
	249^a^ (36)	302 (46)	RMS	Minor	0.94 (0.57–1.55)^b^	
Altmann et al., 1998 [17]	43 (3)	NR (NR)	STS	Any BD	3.5 (1.0–11.8)^b^	DS cases were not excluded. Any BD estimates adjusted for 6-month calendar period, gender, birth weight, gestational age and maternal age.
	23 (3)	NR (NR)	RMS	Any BD	7.9 (2.2–28.8)^b^	
	23 (1)	NR (9)	RMS	Genitourinary	18.2 (2.1–157)^b^	
Merks et al., 2005 [26]	68 (5)	881 (54)	RMS	Cervical	1.22 (0.47–3.15)^a^^,^^b^	
Durmaz et al., 2011[20]	11 (11^a^)	200 (70^a^)	RMS	Any BD	∞ (ND-∞)^a^^,^^b^	Patients with known genetic syndromes were excluded. Data on 19 specific minor anomalies was also reported.
Zierhut et al., 2011 [24]	30 (1)	1,133 (51)	STS and other extraosseous sarcomas	Abnormal ribs	0.64 (0.08–4.91)^b^	Estimates adjusted for sex and age.
	15 (1)	1,133 (51)	RMS	Abnormal ribs	1.35 (0.17–10.62)^b^	
	15 (0)	1,133 (51)	Other specified or unspecified STS	Abnormal ribs	-	
***Soft tissue and other extraosseous sarcomas cohort studies***						
Mili et al., 1993 [29]	2 (10,891)	18 (241,473)	Sarcoma	Any BD	4.1 (0.5–14.8)^d^	Did not exclude any known genetic syndromes. Estimates adjusted for age and sex. Minor defects were included only if they were associated with at least one major defect.
Agha et al., 2005 [33]	7 (45,200)	4 (45,200)	STS	Any BD	1.9 (1.0–3.5)^c^	Did not exclude any known genetic syndromes.
Rankin et al., 2008 [30]	2 (NR)	40^a^ (NR)	RMS	Any BD	2.98 (0.77–11.52)^f^	Did not exclude any known genetic syndromes. RMS includes other STS as noted in a table footnote.
Carozza et al., 2012 [31]	15 (115,686)	102 (3,071,225^a^)	STS	Any BD	2.12 (1.09–3.79)^f^	Genetic syndromes were not excluded. The authors note that excluding subjects with chromosomal anomalies resulted in a lower IRR for leukemia and total cancers but not for other cancers.
Fisher et al., 2012 [36]	6 (59,258)	NR	RMS	Non-chromosomal	2.26 (1.00–5.11)^e^	
	0 (6,327)	NR	RMS	Chromosomal	-	
Botto et al., 2013 [32]	<5 (44,151)	5 (147,940)	RMS	Structural	3.31 (0.9–12.3)^f^	Children with chromosomal anomalies were excluded.
	0 (44,151)	<5 (147,940)	Fibrosarcoma	Structural	-	
	<5 (44,151)	<5 (147,940)	Other STS	Structural	1.38 (0.1–13.8)^f^	
Sun et al., 2014 [38]	1 (4,484)	33 (1,547,126)	Mesothelial and soft tissue (<1 year)	Nervous system (cohort entry on day of birth)	10.47 (1.43–76.58)^e^	Children with chromosomal anomalies were excluded. Estimates adjusted for calendar year and sex.
	NR (NR)	NR (NR)	Mesothelial and soft tissue (<1 year)	Nervous system (cohort entry at BD diagnosis)	24.22 (3.30–177.63)^e^	
	5 (4,484)	87 (1,547,126)	Mesothelial and soft tissue (1–15 years)	Nervous system (cohort entry on day of birth)	19 (7.72–46.81)^e^	
	NR (NR)	NR (NR)	Mesothelial and soft tissue (1–15 years)	Nervous system (cohort entry at BD diagnosis)	23.02 (9.34–56.72)^e^	
	2 (24,643)	33 (1,547,126)	Mesothelial and soft tissue (<1 year)	Circulatory system (cohort entry on day of birth)	3.81 (0.91–15.86)^e^	
	NR (NR)	NR (NR)	Mesothelial and soft tissue (<1 year)	Circulatory system (cohort entry at BD diagnosis)	-	
	4 (4,484)	874 (1,547,126)	Mesothelial and soft tissue (1–15 years)	Circulatory system (cohort entry on day of birth)	2.99 (1.10–8.14)^e^	
	NR (NR)	NR (NR)	Mesothelial and soft tissue (1–15 years)	Circulatory system (cohort entry at BD diagnosis)	0.91 (0.13–6.53)^e^	
Dawson et al., 2015 [34]	2 (32,273)	53^a^ (608,726)	STS	N BDs	0.71 (0.17–2.90)^e^	Excluded syndromes known to be associated with cancer (e.g. DS) (N category).
Janitz et al., 2016 [35]	≤5 (23,368)	<30^a^ (567,867)	STS	Any BD (w/chromosomal)	3.7 (1.3–10.6)^e^	The exact number of STS cases was suppressed.
	≤5 (22,109^a^)	<30^a^ (567,867)	STS	Non-chromosomal	3.9 (1.4–11.2)^e^	
***Soft tissue and other extraosseous sarcomas case series studies with an external comparison group studies***						
Ruymann et al., 1988 [87]	10 (115)	NWTS: NR (1,905); CPP: NR (53,257)	RMS	Genitourinary	NWTS:1.18^a^^,^^c^; CPP: 3.14^a^^,^^c^	Authors presented rates for two external comparison groups: 1) the NWTS and 2) the CPP. Rate ratios were calculated as the ratio of the rate in study population to the rate in the external comparison group.
	9 (115)	NR (1,905); NR (53,257)	RMS	CNS	16.64 ^a^^,^^c^; 18.62 ^a^^,^^c^	
	13 (115)	NR (1,905); NR (53,257)	RMS	Upper alimentary tract/ digestive systems	7.68 ^a^^,^^c^; 7.05 ^a^^,^^c^	
	4 (115)	NR (1,905); NR (53,257)	RMS	Cardiopulmonary	2.68 ^a^^,^^c^; 4.26 ^a^^,^^c^	
	5 (115)	NR (1,905); NR (53,257)	RMS	Accessory spleens	6.9 ^a^^,^^c^; 217 ^a^^,^^c^	
	NR (115)	NR (1,905); NR (53,257)	RMS	Musculoskeletal	0.38 ^a^^,^^c^; 0.31 ^a^^,^^c^	
	NR (115)	NR (1,905); NR (53,257)	RMS	Aniridia	ND	
	NR (115)	NR (1,905); NR (53,257)	RMS	Hemihypertrophy	0.35 ^a^^,^^c^; 43.5 ^a^^,^^c^	
Narod et al., 1997 [53]	NR (2)	NA	RMS	Cardiac septal	1.1 (NRCT); 0.56 (BC)	Two hundred seventy-five cases with an established genetic cause were removed; authors report RRs but do not define the RR abbreviation.
	1,248 (18)	NA	STS	Genitourinary	1.7*; 1.5	
	1,248 (3)	NA	STS	Spine and Rib	1.0; 8.3*	
***Germ cell tumors*, *trophoblastic tumors*, *and neoplasms of gonads case-control studies***						
Swerdlow et al., 1982 [91]	87 (2)	10,128 (NR)	Testicular	Genitourinary	2.99^b^ (P = 0.15)	P-values calculated by Fisher’s exact test.
	87 (2)	10,128 (NR)	Testicular	Inguinal hernias	2.05^b^ (P > 0.05)	
Schumacher et al., 1992 [23]	29 (1^a^)	200 (11)	Yolk sac carcinoma	Rib	0.61 (0.87–4.94)^a^^,^^b^	
Mann et al., 1993 [16]	41 (7)	41 (1)	GCT	Any BD	8.24 (0.96–70.32)^a^^,^^b^	Pedigree was shown for one case with a teratoma.
Shu et al., 1995 [89]	105 (NR)	639 (NR)	GCT	Any BD	1.4 (0.6–3.5)^b^	
Altmann et al., 1998 [17]	21 (4)	NR (NR)	Gonadal and GCT	Any BD	8.9 (2.6–30.3)^b^	DS cases were not excluded. Estimate adjusted for 6-month calendar period, gender, birth weight, gestational age, and maternal age.
Merks et al., 2005 [26]	34 (5)	881 (54)	Germ cell	Cervical	2.64 (0.98–7.09)^a^^,^^b^	
Johnson et al., 2009 [88]	Males: 81^a^ (23); Females: 192^a^ (35)	Males: 179^a^ (27); Females: 240 (40)	GCT	Any BD	Males: 2.5 (1.3–4.9)^b^; Females: 1.1 (0.7–1.8)^b^	Estimates adjusted for child's age.
	82^a^ (8), NA	180^a^ (2), NA	GCT	Cryptorchidism	10.8 (2.1–55.1)^b^; NA	
	83^a^ (6), 193^a^ (2)	181^a^ (7), 241 (9)	GCT	Hernia	1.8 (0.6–5.8)^b^; 0.3 (0.1–1.3)^b^	
	83^a^ (5), 195^a^ (12)	181^a^ (7), 241 (12)	GCT	Congenital heart	1.5 (0.5–5.1)^b^; 1.2 (0.5–2.8)^b^	
	83^a^ (3), 193^a^ (15)	181^a^ (7), 241 (14)	GCT	Large/multiple birthmarks	1.0 (0.2–4.1)^b^; 1.4 (0.6–2.9)^b^	
	83^a^ (2), 195^a^ (5)	180^a^ (3), 241 (2)	GCT	Skeletal	1.6 (0.2–10.0)^b^; 3.1 (0.6–16.4)^b^	
	82^a^ (5), 193^a^ (8)	181^a^ (5), 241 (8)	GCT	Other	3.0 (0.1–11.1)^b^; 1.3 (0.5–3.4)^b^	
Hall et al., 2017 [90]	451 (23)	273,519 (1,527)	GCT	Any BD	9.12 (5.92–14.05)^b^	Estimates adjusted for birth year and maternal race (White v. non-White).
	451 (7)	273,519 (72)	GCT	Ear/face/neck	47.92 (21.86–105.04)^b^	
	181 (2)	273,519 (1,527)	Yolk sac tumors	Any BD	NR	
	181 (0)	273,519 (72)	Yolk sac tumors	Ear/face/neck	-	
	216 (19)	273,519 (1,527)	Teratomas	Any BD	15.43 (9.46–25.18)^b^	
	216 (7)	273,519 (72)	Teratomas	Ear/face/neck	93.7 (42.14–208.32)^b^	
***Germ cell tumors*, *trophoblastic tumors*, *and neoplasms of gonads cohort studies***						
Agha et al., 2005 [33]	3 (45,200)	4 (45,200)	GCT, trophoblastic and other gonadal carcinoma & other	Any BD	0.8 (0.4–1.7)^c^	Did not exclude any known genetic syndromes. O/E ratio is age standardized.
	NA	NA	GCT	Other musculoskeletal	12.9 (1.2–19.4)^g^	
Carozza et al., 2012 [31]	17 (115,686)	60 (3,071,225^a^)	GCT	Any BD	5.19 (2.67–9.41)^f^	Genetic syndromes were not excluded. The authors note that excluding subjects with chromosomal anomalies resulted in a lower IRR for leukemia and total cancers but not for other cancers.
Fisher et al., 2012 [36]	8 (59,258)	NR	Non-CNS GCT	Non-chromosomal	2.98 (1.46–6.07)^e^	
	1 (6,327)	NR	Non-CNS GCT	Chromosomal	4.32 (0.60–30.82)^e^	
Botto et al., 2013 [32]	6 (44,151)	6 (147, 940)	GCT, trophoblastic, and gonadal	Structural	4.14 (1.3–12.8)^f^	Children with chromosomal anomalies were excluded.
	<5 (44,151)	<5 (147,940)	Teratoma	Structural	5.51 (1.2–24.6)^f^	
	<5 (44,151)	<5 (147,940)	Other GCT	Structural	2.76 (0.5–16.5)^f^	
Dawson et al., 2015 [34]	2 (32,273)	28^a^ (608,726)	Gonadal and GCT	N BDs	1.34 (0.32–5.63)^e^	Excluded syndromes known to be associated with cancer (e.g. DS) (N category).
Janitz et al., 2016 [35]	6 (23,368)	11^a^ (567,867)	GCT	Any BD (w/chromosomal)	13.6 (5.0–36.7)^e^	
	6 (22,109^a^)	11^a^ (567,867)	GCT	Non-chromosomal	14.3 (5.3–38.7)^e^	
***Germ cell tumors*, *trophoblastic tumors*, *and neoplasms of gonads nested case-control studies***						
Wanderas et al., 1998 [92]	32 (0)	3,028 (99)	Testicular cancer	Any BD	-	0–4 year olds
***Germ cell tumors*, *trophoblastic tumors*, *and neoplasms of gonads case series studies with an external comparison group studies***						
Narod et al., 1997 [53]	544 (43)	NA	Gonadal and GCT	Total	1.9*** (NRCT); 1.3 (BC)	Two hundred seventy-five cases with an established genetic cause were removed; authors report RRs but do not define the RR abbreviation.
	544 (3)	NA	Gonadal and GCT	Other nervous (742)	3.7*; 3.2	
	544 (3)	NA	Gonadal and GCT	Septal defects (745)	2.6; 1.3	
	544 (18)	NA	Gonadal and GCT	Musculoskeletal (754–6)	2.7***; 1.9**	
	544 (6)	NA	Gonadal and GCT	Spinal (7561)	5.7***; 54.5***	
	544 (1)	NA	Gonadal and GCT	Spina bifida	1.5; 3.5	
	544 (6)	NA	Gonadal and GCT	Genitourinary	1.3; 1.1	
	544 (6)	NA	Gonadal and GCT	Spine and rib	4.3**; 37.5***	
	544 (19)	NA	Gonadal and GCT	Other	1.4; 0.93	
	NR (3)	NA	GCT	Cardiac septal	6.4*; 3.3	
***Other and unspecified malignant tumors case-control studies***						
Méhes et al., 1985 [19]	55 (35)	Siblings: 44 (26); Controls: 55 (17)	Solid	Minor	1.21 (0.54–2.74)^a^^,^^b^; 3.91 (1.77–8.65)^a^^,^^b^	
Mann et al., 1993 [16]	22 (1)	28 (0)	Epithelial	Any BD	-	
	28 (1)	28 (1)	Other	Any BD	1.00 (0.06–16.82)^a^^,^^b^	
Altmann et al., 1998 [17]	7 (0)	NR	Carcinoma, malignant epithelial	Any BD	-	DS cases were not excluded. Estimate adjusted for 6-month calendar period, gender, birth weight, gestational age, and maternal age. Cases with other malignancy types had no malformations.
	2 (0)	NR	Other	Any BD	-	
Merks et al., 2005 [26]	124 (8)	881 (54)	Other	Cervical	1.06 (0.49–2.28)^a^^,^^b^	
Loder et al., 2007 [25]	72^a^ (10)	200 (16)	Solid tumor	Abnormal rib number (normal = 24 ribs)	1.90 (0.80–4.30)^b^	Solid tumors included WT (n = 17), OS (n = 14), NB (n = 8), RMS (n = 5), Ewing sarcoma (n = 3), and other types (n = 9). Children with a known BD or DS were excluded.
Durmaz et al., 2011 [20]	14 (14^a^)	200 (70^a^)	WT/GCT	Any minor	∞ (NR-∞)^a^^,^^b^	Patients with known genetic syndromes were excluded. Data on 19 specific minor anomalies was also reported.
	12 (10^a^)	200 (70^a^)	Others	Any minor	9.3 (2.0–43.6)^a^^,^^b^	
Santos et al., 2016 [109]	281 (55)	176 (29)	Solid tumor	Solitary café-au-lait spots	1.23 (0.75–2.02)^a^^,^^b^	A known syndrome diagnosis was suspected in 4 cases
	281 (28)	176 (12)	Solid tumor	Multiple café-au-lait spots	1.51 (0.75–3.06)^a^^,^^b^	
***Other and unspecified malignant tumors cohort studies***						
Windham et al., 1985 [27]	5 (22,856)	NR (NR)	Other	Any BD	1.2 (0.5–3.2)^c^	Did not exclude any known genetic syndromes. Estimates are age standardized.
	NR (NR)	NR (NR)	Other	Any BD (Male)	0.4^c^	
	NR (NR)	NR (NR)	Other	Any BD (Female)	2.2^c^	
	NR (NR)	NR (NR)	Other (0–4 years)	Any BD (Male)	^-^	
	NR (NR)	NR (NR)	Other (0–4 years)	Any BD (Female)	3.1^c^	
	NR (NR)	NR (NR)	Other (5–9 years)	Any BD (Male)	2.2 ^c^	
	NR (NR)	NR (NR)	Other (5–9 years)	Any BD (Female)	-	
Agha et al., 2005 [33]	1 (45,200)	1 (45,200)	Other	Any BD	1.1 (0.3–4.4)^c^	Did not exclude any known genetic syndromes. O/E ratios are age standardized.
	3 (45,200)	2 (45,200)	Malignant epithelial	Any BD	1.6 (0.6–4.0)^c^	
	NA	NA	Carcinomas	Respiratory system	10.9 (1.5–77.2)^g^	
	NA	NA	Carcinomas	Urinary system	11.8 (1.7–83.7)^g^	
Rankin et al., 2008 [30]	0 (NR)	73^a^ (NR)	All other cancers	Any BD	-	Did not exclude any known genetic syndromes.
Carozza et al., 2012 [31]	2 (115,686)	19 (3,071,225^a^)	Other epithelial	Any BD	2.98 (0.34–12.34)^c^	Genetic syndromes were not excluded. The authors note that excluding subjects with chromosomal anomalies resulted in a lower IRR for leukemia and total cancers but not for other cancers.
	1 (115,686)	11 (3,071,225^a^)	Other and unspecified	Any BD	5.05 (0.12–34.75)^c^	
Botto et al., 2013 [32]	0 (44,151)	<5 (147, 940)	Miscellaneous	Structural	ND	Children with chromosomal anomalies were excluded.
Sun et al., 2014 [38]	3 (4,484)	91 (1,547,126)	Other systems (<1 year)	Nervous system (cohort entry on day of birth)	11.4 (3.61–36.0)^e^	Children with chromosomal anomalies were excluded. Estimates adjusted for calendar year and sex.
	NR (NR)	NR (NR)	Other systems (<1 year)	Nervous system (cohort entry at BD diagnosis)	15.73 (3.87–63.92)^e^	
	7 (4,484)	563 (1,547,126)	Other systems (1–15 years)	Nervous system (cohort entry on day of birth)	4.1 (1.95–8.65)^e^	
	NR (NR)	NR (NR)	Other systems (1–15 years)	Nervous system (cohort entry at BD diagnosis)	2.84 (1.06–7.59)^e^	
	5 (4,484)	75 (1,547,126)	Other systems (<1 year)	Circulatory system (cohort entry on day of birth)	4.19 (1.69–10.35)^e^	
	NR (NR)	NR (NR)	Other systems (<1 year)	Circulatory system (cohort entry at BD diagnosis)	1.57 (0.22–11.30)^e^	
	22 (4,484)	464 (1,547,126)	Other systems (1–15 years)	Circulatory system (cohort entry on day of birth)	3.1 (2.02–4.76)^e^	
	NR (NR)	NR (NR)	Other systems(1–15 years)	Circulatory system (cohort entry at BD diagnosis)	0.68 (0.25–1.82)^e^	
Dawson et al., 2015 [34]	2 (32,273)	33^a^ (608,726)	Other epithelial/ melanoma	N BDs	1.17 (0.28–4.88)^e^	Excluded syndromes known to be associated with cancer (e.g. DS) (N category).
	18 (32,273)	235^a^ (608,726)	Embryonal	N BDs	1.43 (0.88–2.30)^e^	
	3 (32,273)	72^a^ (608,726)	Other	N BDs	0.80 (0.25–2.53)^e^	
***Other and unspecified case series studies with an external comparison group studies***						
Narod et al., 1997 [53]	NR (6)	NA	Carcinoma/Other	Genitourinary	1.1; 1.0	Two hundred seventy-five cases with a known genetic cause were removed; the RR abbreviation in undefined.
	NR (1)	NA	Carcinoma	Cardiac septal	0.78; 0.40	

^¥^For case-control and nested case-control studies the total number of cancer cases (comparison group 1) and non-cancer cases (comparison group 2) is shown in columns 2–3 with the number in parentheses indicating the number of individuals with BDs in each comparison group. For cohort studies, comparison group 1 is noted as above; comparison group 2 shows the number of cancer cases among the number without BDs in parentheses. For case-cohort studies, comparison group 1 is the same as for other study designs; comparison group 2 shows the total number of individuals with cancer among the total number of individuals in the sub-cohort in parentheses.

*p<0.05;

**p<0.01;

***p<0.001;

^a^Calculated based on information;

^b^Odds Ratio;

^c^Rate Ratio

^d^Standardized Incidence Ratio;

^e^Hazard Ratio;

^f^Incidence Rate Ratio;

^g^O/E ratio;

^h^Relative Risk

We identified 24 articles reporting findings for associations between pediatric cancer overall and BDs.

#### Case-control studies (n = 12)

Three studies reported ~2–4.5 fold increased odds of childhood cancer in association with any/BDs or major BDs [15–17], while one reported no association [18]. For minor anomalies, four studies reported increased odds of varying magnitude for minor anomalies overall (ORs = 4.25 and 44.6 [19, 20]), and specific minor anomalies (OR >2-fold [21, 22]). One study reported positive associations ranging from 2.6–14.5 for a number of BD types [17]. Finally, four studies reported generally positive associations between rib anomalies and childhood cancer with ORs ranging from 1.44 to 5.49 [23–26].

#### Cohort studies (n = 12)

Consistently elevated risks ranging from 1.8–3.05 were reported for associations between any BDs/major BDs and pediatric cancers [27–35]. Increased cancer risks were within the same range or lower when children with chromosomal anomalies were excluded [32, 34–36]. One cohort study reported increased risks in children with different BD types ranging from 1.69–15.52 with the exception of musculoskeletal abnormalities and limb reduction defects [31]. Janitz et al. reported increased risks for CNS, eye/ear, cardiovascular, orofacial, gastrointestinal, genitourinary, and musculoskeletal abnormalities with stronger risks at younger ages (except orofacial where results were not reported) and when individuals with chromosomal BDs were excluded [35]. Birthmarks (not including nevus simplex or pigmented nevi or café-au-lait spots <3 cm or fewer than 6) were positively associated with childhood cancers (HRs 2.81 and 2.03 for those diagnosed at 0–8 and 1–8 years respectively) [37]. Botto et al. examined a number of specific BD types in children without chromosomal anomalies observing that a majority increased risk [32]. Finally, Sun et al. reported BDs of both the circulatory and nervous system were positively associated with pediatric cancer with generally weaker associations for older children and when the follow-up period started at the time of BD diagnosis [38].

### Leukemia (Table 3)

We identified 36 articles reporting findings for associations between leukemia and hematological malignancies and BDs.

#### Case-control studies (n = 24)

Risk estimates ranged from 1.07 to 11.6 [15–17, 39–47] for associations between any/major BDs and leukemia overall or specific leukemia subtypes. Shu et al. observed no leukemia cases with BDs other than one case with Down syndrome [48]. Studies excluding DS cases from overall or sub-analyses generally reported weaker associations [39–41, 44–46, 49]. Several studies reported positive associations between minor BDs and leukemia/hematological malignancies ranging from 1.48 to 67.48 [19, 20, 45, 50–52].

Cleft lip or palate was inconsistently associated with lymphatic and myeloid leukemia [40, 43, 49]. Several studies observed positive associations between hematologic malignancies and birthmarks [43, 46, 50] and rib anomalies [23–26]. Minor BDs of the hand, foot, eye, nose, mouth, and ear were all positively associated with hematological malignancies in one study [20]. Finally, inconsistent results were reported for a number of other specific BDs [41, 43, 45, 46, 53].

#### Cohort studies (n = 11)

In six studies, positive associations were observed between leukemias and any BDs/major BDs ranging from 1.1–21.97 [27–31, 33]. Associations were generally weaker in studies/analyses with DS cases were excluded [27–29, 31, 32, 34–36]. Rankin et al. noted, however, that “the association between congenital anomalies and childhood leukemia remained after exclusion of Down Syndrome cases” [30]. Notably, Dawson et al. reported a positive association between other leukemias (types other than ALL or AML) and non-chromosomal BDs [34]. Finally, Sun et al. examined nervous and circulatory system BDs and risk of lymphatic and haematopoietic tissue malignancies in children without chromosomal anomalies and reported positive associations that were stronger for infants versus children 1–15 years. Associations were generally weaker when follow-up time was counted from the BD diagnosis rather than from birth [38].

#### Case series with an external comparison group studies (n = 1)

Narod et al. examined a number of specific abnormalities and did not report any consistently increased risks for leukemia [53].

### Lymphoma (Table 3)

We identified 17 articles reporting findings for associations between lymphoma and BDs.

#### Case-control studies (n = 9)

Four studies reported inconsistent associations between lymphoma/lymphoma subtypes and any BDs/major BDs with ORs ranging from 0.7–4.43 [15–17, 54]. Based on 19 lymphoma cases, Mangani et al. reported 0 lymphoma cases with BDs [47]. Rib anomalies specifically were inconsistently associated with lymphoma subtypes [23, 24, 26]; however, one study combining lymphoma with leukemia in their analysis reported a significant OR of 2.0 [25].

#### Cohort studies (n = 7)

Lymphoma was positively associated with any BD in three of four cohort studies [30, 31, 33, 35]. Fisher et al. and Janitz et al. reported positive associations between non-chromosomal anomalies and lymphoma [35, 36], while both Botto et al. and Dawson et al. observed risks for lymphoma/lymphoma subtypes in both directions in children with non-chromosomal BDs and those not known to be related to cancer [32, 34]. Certain musculoskeletal anomalies were associated with lymphoma in one study [33].

#### Case series with an external comparison group studies (n = 1)

Narod et al. reported findings for cardiac septal defects, genitourinary abnormalities, and spine and rib abnormalities with a significant positive association for spine and rib abnormalities for the British Columbia registry comparison group only [53].

### CNS and miscellaneous intracranial and intraspinal neoplasms (Table 3)

We identified 31 articles reporting findings for associations between CNS tumors and BDs.

#### Case-control and nested case-control studies (n = 19)

Risk estimates for associations between CNS tumors and any BDs/major BDs ranged from 1.0 to 4.7 [15–17, 55–60]. Two studies did not report risk estimates; however, one reported no association and the other reported a decreased frequency of BDs (excluding minor BDs) in cases versus controls [61, 62]. Altmann et al. reported an increased odds of astrocytoma in children with any BD [17]. However, three studies [58, 59, 63] found weak to no evidence that children with any BD/major BDs have an increased risk for specific CNS subtypes with the exception of other gliomas [58] where a 2–3 fold increased risk was reported. Importantly, these results were unchanged after excluding seven CNS tumor cases with Neurofibromatosis Type 1 [58]. Partap et al.’s results contrast with these findings by showing increased odds of a BD history for MBs and PNETs compared to controls and inverse associations for gliomas [57].

Gold et al. reported a strong positive association only between brain tumors and club foot [64]. Another study reported universally positive associations for hand, foot, eye, nose, mouth, and ear minor BDs [20]. Birthmarks/deformities were not associated with brain tumors in one study [65]. Altmann et al. reported highly significant and strong associations between CNS tumors and nervous system and eye/face/neck abnormalities [17]. Finally, several investigators reported that brain tumor cases with varying subtypes had higher odds of rib abnormalities than controls [23–26].

#### Cohort studies (n = 11)

Six studies reported positive associations for any BD and CNS tumors ranging from 1.11 to 2.9 [27–31, 33]. Fisher et al. reported increased HRs for CNS tumors in both children with and without chromosomal BDs of 1.87 and 1.80 [36]. Botto et al. reported an increased brain tumor incidence in children with structural BDs that was stronger for other brain tumors [32]. Dawson et al. reported a weak imprecise association of 1.26 between BDs not known to be related to cancer and CNS tumors [34]. Janitz et al. reported increased risks in children with non-chromosomal BDs that decreased with increasing age [35].

For specific BD types, nervous system abnormalities were strongly associated with CNS tumors in three studies [27, 33, 38]. Finally, circulatory system malformations were positively associated with CNS tumors in one study [38].

#### Case series with an external comparison group studies (n = 1)

Narod et al. reported inconsistent or inverse associations for total BD and most specific abnormalities with the exception of hydrocephalus where both RRs were positive and significant [53].

### Neuroblastoma and other peripheral nervous cell tumors (Table 3)

We identified 26 articles reporting findings for associations between NB and BDs.

#### Case-control studies (n = 14)

Risk estimates from 11 studies reporting associations between any BDs/physical anomalies and NB/sympathetic nervous system tumors ranged from 1.0 to 8.61 [16, 17, 66–74]. Major and minor BDs were positively associated with NB in four [69–71, 74] and two studies, respectively [69, 71]. Several studies examining specific BDs generally observed positive associations [69–71], particularly for digestive system/gastrointestinal and genitourinary anomalies [17, 69, 71, 74] and during infancy [69]. Rib anomalies were positively associated with NB/other peripheral nerve tumors in three studies to varying degrees [23, 24, 26].

#### Cohort and case-cohort studies (n = 11)

Children with any BD had a consistently higher risk of NB across studies with RRs from 1.1–20.3 [27–31, 33, 75]. Fisher et al. and Botto et al. [32, 36] also reported that non-chromosomal BDs were associated with NB and other peripheral nervous system tumors with individuals in the BD group having a >2-fold higher risk, while Dawson et al. reported a lower risk estimate of 1.41 for individuals with BDs not known to be related to cancer [34]. Finally, a case-cohort study reported a similar percentage of cases and sub-cohort members had BDs recorded in their birth records [76]. For specific anomalies, Agha et al. reported more observed than expected cases of SNS tumors in those with other anomalies of the digestive system [33].

#### Case series with an external comparison group studies (n = 1)

Narod et al. reported results that were imprecise and in opposite directions for total BD. Generally positive associations were observed for specific BDs, particularly for cardiac and gastrointestinal abnormalities [53].

### Retinoblastoma and eye tumors (Table 3)

We identified 10 articles reporting findings for associations between RB/eye tumors and BDs.

#### Case-control studies (n = 2)

Results were mixed with Mann et al. finding no BDs in RB cases and Altmann et al. reporting a strong positive OR of 15.0 [16, 17]. For specific BDs, strong associations were reported for chromosomal anomalies in a single study.

#### Cohort and case-cohort studies (n = 7)

Of seven studies, six reported positive associations ranging from 2.2–4.7 between BDs overall or those that excluded individuals with chromosomal anomalies [27, 28, 30–33]. Dawson et al. reported no RB cases [34]. Chromosomal and eye anomalies were strongly associated with RB in one study [33].

#### Case series with an external comparison group studies (n = 1)

Narod et al. reported inconsistent results for associations between RB and BDs and strong associations between RB and cataracts and ventricular septal defects [53].

### Renal tumors (Table 3)

We identified 21 articles reporting findings for associations between renal tumors and BDs.

#### Case-control and nested case-control studies (n = 10)

Wilkins et al. reported similar frequencies of any BD in WT cases and controls [77], while two other groups reported positive associations [16, 17]. Stronger associations were reported for non-WT associated BDs than WT-associated BDs in one study [78]. Two studies reported significant positive associations between WT and spina bifida [79, 80]. WT was also reported as positively associated with eye/face/neck BDs and chromosomal BDs [17]. WT/renal carcinomas were positively associated with rib abnormalities in three studies [23, 24, 26]. Finally, Lindblad et al. reported that associations between BDs and WT were not confirmed in a nested case-control study [81].

#### Cohort and case-cohort studies (n = 9)

Risk estimates for cohort and case-cohort studies examining associations between any BD and WT or renal tumors ranged from 1.0–3.2 [27, 28, 30, 31, 33, 82]. Fisher et al. reported significant risks for WT only in children with chromosomal BDs [36]. In two studies excluding children with chromosomal anomalies and syndromes known to predispose toward cancer, results were mixed [32, 34].

#### Case series with an external comparison group studies (n = 2)

Breslow et al. compared the rate of different BD types in a case series to two external comparison groups and observed consistent strongly increased rates of aniridia, double-collecting system, and hemihypertrophy [83]. Narod et al. reported consistent findings across the two different comparison groups for genitourinary and reproductive organ associated BDs. Renal carcinomas were strongly associated with spine and rib malformations in one study [53].

### Hepatic tumors (Table 3)

We identified 10 articles reporting associations between hepatic tumors and BDs.

#### Case-control studies (n = 3)

There was no significant association between HB and any BDs in one study [16], while another reported a non-significant 9.3-fold increased odds of BDs in hepatic tumor cases versus controls [17]. For specific abnormalities, both spina bifida and genitourinary conditions were positively associated with HB in one study [84]. Other abnormalities were examined only in single studies.

#### Cohort and case-cohort studies (n = 6)

One study reported three hepatic tumor cases in individuals with BDs and none in the comparison group [33]. Carozza et al. reported no association between liver tumors and any BD [31], while Spector et al. reported an almost 6-fold increased risk of HB in association with any BD [85]. Both Botto et al. and Dawson et al. reported strongly increased IRRs for liver tumors in individuals with BDs after exclusion of children with chromosomal BDs and syndromes known to be associated with cancer [32, 34]. In the Janitz et al. study, there were too few hepatic tumor cases to examine statistical associations [35]. Digestive and chromosomal anomalies were positively associated with hepatic tumors in one study [33].

#### Case series with an external comparison group studies (n = 1)

Narod et al. reported positive associations between spina bifida and genitourinary abnormalities and HB [53].

### Malignant bone tumors (Table 3)

We identified 12 articles reporting associations between BDs and bone tumors.

#### Case-control studies (n = 7)

One study reported no association between OS and any BDs [12]. Two studies reported positive but imprecise associations (ORs = 1.56 and 23.2) between bone cancer and any BD [16, 17]. Minor BDs were associated with OS in one study (OR = 12.4) [20]. One study reported a positive association between bone tumors and chromosomal anomalies [17]. Finally, three studies reported inconsistent associations between rib anomalies and bone cancer (OS or ES) [23, 24, 26].

#### Cohort studies (n = 4)

Mixed results were reported for any BD/BDs not known to be related to cancer and bone cancer in three studies [31, 33, 34]. Botto et al. reported a two-fold non-significant increased incidence of ES in individuals with non-chromosomal structural BDs [32]. An ~ 4-fold excess of bone tumors was reported in children with other musculoskeletal BDs in one study [33].

#### Case series with an external comparison group studies (n = 1)

Narod et al. reported consistently strong associations using two different comparison groups between bone tumors overall and spina bifida; however, the association was based on only one case. Strong positive associations were also reported between osteodystrophy and cataracts and ES using both comparison groups [53].

### Soft tissue and other extraosseous sarcomas (Table 3)

We identified 19 articles reporting associations between BDs and soft tissue and other extraosseous sarcomas.

#### Case-control studies (n = 8)

Four studies reported positive associations between any BDs/major BDs and STS/RMS ranging from 1.3 to 7.9 [15–17, 86]. Minor BDs were observed in 100% of RMS cases and only 35% of controls in one study [20]. Further evidence for a positive association between genitourinary malformations and STS was reported for RMS [17]. Three studies examined associations between rib anomalies and STS/RMS reporting inconsistent associations [23, 24, 26]. Minor BDs were not positively associated with RMS [86].

#### Cohort studies (n = 9)

Five cohort studies examined associations between any BDs and STS/RMS, with all finding positive associations ranging from 1.9 to 4.1 [29–31, 33, 35]. Three studies reported positive associations between RMS and non-chromosomal (structural) BDs, while one study reported a non-significant inverse for STS for BDs not known to be related to cancer [32, 34–36]. Finally Sun et al., reported strong positive associations between nervous system BDs and mesothothelial and soft tissue cancers across age groups and weaker associations for circulatory system BDs [38].

#### Case series with an external comparison group studies (n = 2)

One study reported markedly higher rates of CNS anomalies, upper alimentary tract/digestive systems, cardiopulmonary anomalies, and accessory spleens in the RMS cohort than in the two comparison groups [87]. Narod et al. reported that genitourinary and spine and rib malformations were positively associated with STS but this was inconsistent across comparison groups used for RR calculations. Cardiac septal defects were not significantly associated with RMS [53].

### Germ cell tumors (GCT), trophoblastic tumors, and neoplasms of gonads (Table 3)

We identified 16 articles reporting associations between BDs and GCTs, trophoblastic tumors, and neoplasms of gonads.

#### Case-control and nested case-control studies (n = 9)

Several studies reported positive associations between any BDs and gonadal tumors/GCTs ranging from 1.1 to 9.12 [16, 17, 88–90]. Genitourinary defects and inguinal hernias were positively associated with testicular tumors [91]. Johnson et al. reported a strong association with cryptorchidism in males [88]. Merks et al. reported an ~3-fold increased odds of cervical anomalies in GCT cases compared to controls [26]. Hall et al. reported an increased odds of ear/face/neck anomalies in GCT cases and those with teratomas specifically [90]. In a nested case-control study, no cases had BDs in the 0–4 year old age group [92].

#### Cohort studies (n = 6)

Among two cohort studies, Agha et al. reported no association between germ cell, trophoblastic and other gonadal carcinoma and any BD, while Carozza et al. reported a significant 5-fold increased risk [31, 33]. Fisher et al., Botto et al., and Janitz et al. reported significant positive associations between GCTs/Gonadal and GCT and non-chromosomal BDs, while Dawson et al. reported a weak positive association for BDs not known to be related to cancer [32, 34–36]. GCTs were strongly associated with other musculoskeletal anomalies in one study [33].

#### Case series with an external comparison group studies (n = 1)

Narod et al. reported consistent increased risks between gonadal and GCT tumors and total BDs and weak non-significant associations between these tumors and genitourinary defects. Musculoskeletal, spinal, and spine and rib malformations were consistently positively associated with [53].

### Other tumors (Table 3)

Several studies examined other childhood cancers and various subtype groupings for their association with BDs. For completeness, we include these results; however, given the heterogeneous nature of the BDs/cancer types and outcomes examined, we do not summarize the findings.

### Quality assessment (S2 and S3 Tables)

The mean percent total quality point score was higher for cohort (88% ± 13%) than case-control (62% ± 19%) studies. The three case-cohort study reports that were based on the same parent study were of high quality each receiving 89% of the total quality points, while the three nested case-control studies received a mean of ~85% ± 6% of the total quality points (data not shown).

## Discussion

Associations between BDs and pediatric cancer have been extensively studied. Overall conclusions on pediatric cancer risk in children with BDs are limited by heterogeneity in study design, subject selection (including inclusion/exclusion criteria), measurement, definitions, and length of follow-up for ascertaining BDs, as well as covariate adjustment in models. For example, in studies ascertaining individuals with BDs from BD surveillance systems, standardized definitions using published classification schemes were used to group individuals with BDs according to BD type (e.g. major, minor, and specific BD types), while for studies ascertaining BDs through parental questionnaire, coding schemes were often elusive. We strongly emphasize that future studies on this topic should employ and clearly report in their methods a standardized classification system such as that reported by Rasmussen et al. for the National Birth Defects Prevention Study [93], which will facilitate pooling and meta-analysis studies that are needed to more precisely quantify pediatric cancer risk in children with BDs. In spite of the differences in methodology used by studies on this topic that limit overall conclusions, several noteworthy findings emerged from this review.

An increased risk for pediatric cancer overall in association with BDs clearly exists with most case-control and cohort studies reporting positive associations. A seminal Nordic study of 5.2 million individuals, not included in our review because it did not present pediatric cancer specific risk estimates, linked medical birth and cancer registries to examine cancer risk in children with BDs [94]. Cancer risk was significantly increased in individuals with non-chromosomal BDs by ~1.4–1.5 fold, with stronger risks at younger ages, which was also reported in several studies included in this review [34, 35, 38]. Moreover, a strong association was observed in both Norway and Sweden (>8 fold) between nervous system abnormalities and CNS tumors [94]. Collectively, our review and the Nordic data provide strong evidence for an overall increased cancer risk in children with BDs.

For leukemia, most studies excluding DS cases reported relatively weak or no evidence for an increased risk of leukemia in children with any BD/major BDs. For example, when considering the results from several cohort studies with overall higher quality than case-controls studies, generally weak associations were reported between BDs and leukemia when DS cases were excluded [28, 29, 32, 34–36] with the exception of one study [30]. It is noteworthy that consistent associations with rib anomalies and minor malformations have been reported in several studies [19, 20, 23–26, 50, 52]. These data suggest that minor BDs may be linked to leukemia development but most of the observed positive associations between major BDs and leukemia are likely explained by inclusion of DS cases.

For CNS tumors, strong consistent associations with CNS abnormalities were reported in several studies [17, 27, 33, 38, 53]. When interpreting these results, it is important to consider the timing of the BD diagnosis. BDs detected only as a result of tests or procedures associated with the tumor diagnosis may lead to over-ascertainment of BDs in cases compared to controls, a potential issue raised by Altmann et al. [17]. In addition, it is also important to consider whether the abnormality occurred secondary to the tumor, a concern noted by Narod et al. for the association between brain/spinal tumors and hydrocephalus [53]. Evidence against these possibilities was provided by Sun et al. who reported a strong positive association between nervous system abnormalities and CNS tumors when follow-up was initiated at the point of the BD diagnosis (i.e. prior to the cancer diagnosis) [38].

For NB, many studies reported consistent positive associations with BDs overall, although there was inconsistency for specific BD types [17, 26, 28–34, 36, 53, 66–75] with the possible exception of gastrointestinal BDs [17, 53, 69, 71]. It is important to note that some of the observed anomalies were noted as secondary to the tumor [67, 71]. Further research is needed to clarify specific abnormalities associated with NB and to confirm the timing of the abnormality as congenital.

For RB/eye tumors, most cohort studies reported consistent positive associations with any BD and structural defects [27, 28, 30–33], some of which may be explained if cases with partial monosomy 13q were included that is associated with a number of anomalies and an increased RB risk [95]. Since ~ 40% of RB tumors arise from a germline mutation [96], there is biological plausibility for developmental abnormalities associated with RB haploinsufficiency. However, despite mouse studies demonstrating lethality for embryos with *Rb1-/-* genotypes as well as neural and hematopoietic abnormalities; heterozygotes did not have any observable defects [97].

WT was associated with a number of abnormalities, some of which are due to known syndromic causes of WT including Beckwith-Wiedemann and WAGR (Wilms tumor, Aniridia, Genitourinary anomalies and intellectual disability) syndromes. WAGR syndrome is caused by a chromosome 11p deletion and is associated with genitourinary abnormalities and aniridia (absence of the colored part of the iris) [98]. In two recent cohort studies excluding children with chromosomal anomalies, associations between BDs and WT were weak and imprecise [32, 36]. It is interesting to note, however, that a greater frequency of non-WT associated BDs were observed in children with WT versus controls in one study [78], a finding that could suggest an underlying genetic predisposition.

For liver cancer, where most cases are HBs, most studies provided evidence of a positive association with any BD [17, 32, 34, 85] that may stem from genitourinary abnormalities that were positively associated with HB in three different study populations [53, 84]. It is unclear if inclusion of Beckwith-Wiedemann Syndrome or Familial Adenomatous Polyposis cases in some of studies explains the observed associations.

Although a three studies detected positive associations between BDs (especially for bone BDs) and bone tumors or bone tumor subtypes [16, 17, 53], cohort study results are mixed and based on small numbers [31–34]. For STSs, increased risks in children with BDs are evident from most case-control and cohort studies [15–17, 20, 29–33, 35, 36, 86]. However, as with other cancer types, conclusions about particular abnormalities associated with risk are limited by the heterogeneous nature of BDs observed/assessed in different studies.

For germ cell and other gonadal tumors, there is a relatively consistent pattern of an increased risk in association with BDs across studies [16, 17, 31, 32, 34–36, 53, 88, 89] that could be partially be due to cryptorchidism but only one study provided risk estimates in males [88].

Although not completely consistent, one of the most intriguing findings is the observed association between rib anomalies and many different childhood cancer types [23–26]. Although some of these associations could be due to detection bias as a result of diagnostic tests for malignancy, the Zierhut et al. study was not subject to this type of bias [24].

The biology underlying associations between pediatric cancer and BDs is poorly understood. If the observed statistical associations are causal, a leading theory is that a common genetic abnormality impairing normal development may subsequently predispose toward both BDs and malignancy [99–103]. Since family history is absent in most individuals with non-syndromic BDs, genetic aberrations could arise through *de novo* mutations in the parental germline or through somatic mutations or epimutations arising early in development. For example, Beckwith-Wiedemann syndrome, can result from genetic and epigenetic mosaicism accompanied by a variably increased risk of malignancy (typically HB or WT) in the affected tissues depending on the causative genetic lesion [104]. The striking number of studies showing an association between rib abnormalities and pediatric/adolescent cancer risk may provide an important clue to underlying genetic commonalities. The finding has frequently been attributed to possible mutations in homeobox genes [25, 53] but no genetic cause has been identified so far explaining this statistical observation.

Our study is the first systematic and most comprehensive review on this topic to date. However, there are limitations to consider. At the expense of specificity, we used broad search terms to capture articles examining associations between BDs and pediatric cancers, identifying >14,000 articles. To be as comprehensive as possible, we identified additional relevant articles through review of article references lists, IARC’s review [11], and PubMed searches. Despite these efforts, it is still possible that we missed some eligible articles; however, it seems unlikely that they are systematically missing and therefore their exclusion should not bias our overall conclusions. In addition, we did not include gray literature [105] (e.g. meeting abstracts and PhD theses) and therefore publication bias may influence general conclusions.

### Conclusions and future directions

Clear positive associations exist between BDs and pediatric cancer with evidence for increased risks for specific cancer/BD type combinations such as CNS abnormalities and CNS cancer, rib anomalies and a number of cancer types, and genitourinary abnormalities and HB. With advances in mutation detection through next generation sequencing technologies it may be possible to identify genetic causes underlying some of these cases, which will provide insight into overlaps between genes impacting both development and malignancy and provide a basis for identification of high risk populations among children with congenital abnormalities.

## Supporting information

S1 Checklist PRISMA 2009 checklist. (DOC)

S1 Table Search strategy. ^a^Search run without the english language limit.
(DOCX)

S2 Table Quality metrics for case-control, nested case-control, and case-cohort studies. ^a^Case-cohort study
^b^Nested case-control study
(DOCX)

S3 Table Quality metrics for cohort studies. (DOCX)
