# Supplementary material for: Pediatric cancer risk in association with birth defects: A systematic review
Source: PLoS One. 2017 Jul 27;12(7):e0181246. doi: 10.1371/journal.pone.0181246 (PMC5716403; doi:10.1371/journal.pone.0181246)
Supplement: S1 Table — aSearch run without the english language limit. (DOCX) [file pone.0181246.s002.docx]

| **S1 Table.** Search strategy. | | | |
| --- | --- | --- | --- |
| **Database name (interface)** | **Search strategy** | **Result (No. of articles found)** | **Date of search** |
| Medline (Pubmed) | Search (((((((("Congenital Abnormalities"[Mesh] OR "Congenital Abnormalities"[tiab] OR "congenital abnormality"[tiab] OR "congenital defect"[tiab] OR "congenital defects"[tiab] OR "birth defect"[tiab] OR "birth defects"[tiab] OR "birth characteristics"[tiab] OR "congenital anomaly"[tiab] OR "congenital anomalies"[tiab] OR "birth anomaly"[tiab] OR "birth anomalies"[tiab] OR "birth malformations"[tiab] OR "congenital malformation"[tiab] OR "congenital malformations"[tiab])))) AND (((("Neoplasms/epidemiology"[Mesh] OR "Tumor"[tiab] OR "Tumors"[tiab] OR "Tumour"[tiab] OR "Tumours"[tiab] OR "cancer"[tiab] OR "cancers"[tiab] OR "Neoplasia"[tiab] OR "Neoplastic"[tiab] OR "Neoplasm"[tiab] OR "Neoplasms"[tiab] OR "carcinoma"[tiab] OR "malignancy"[tiab] OR "malignancies"[tiab] OR "malignant"[tiab]) OR (("Leukemia"[Mesh] OR "Myeloproliferative Disorders"[Mesh] OR "Myelodysplastic-Myeloproliferative Diseases"[Mesh] OR "Lymphoma"[Mesh]) OR ("Central Nervous System Neoplasms"[Mesh] OR "Glioma"[Mesh] OR "Neuroectodermal Tumors, Primitive"[Mesh]) OR ("Neuroblastoma"[Mesh] OR "Retinoblastoma"[Mesh] OR "Kidney Neoplasms"[Mesh] OR "Liver Neoplasms"[Mesh] OR "Hepatoblastoma"[Mesh] OR "Bone Neoplasms"[Mesh] OR "Osteosarcoma"[Mesh] OR "Chondrosarcoma"[Mesh] OR "Sarcoma"[Mesh] OR "Adrenocortical Carcinoma"[Mesh] OR "Thyroid Neoplasms"[Mesh] OR "Nasopharyngeal Neoplasms"[Mesh] OR "Melanoma"[Mesh] OR "Neoplasms, Germ Cell and Embryonal"[Mesh] OR "Neoplasms, Gonadal Tissue"[Mesh]))))) AND (((((("Risk"[Mesh] OR "Risk Assessment"[Mesh] OR "Risk Factors"[Mesh] OR "Odds Ratio"[Mesh] OR "Incidence"[Mesh] OR "Prevalence"[Mesh] OR "Probability"[Mesh:noexp] OR "Epidemiologic Studies"[Mesh]) OR (risk[ti] OR odds[ti] OR likelihood[ti] OR incidence[ti] OR prevalence[ti] OR propensit*[ti] OR probabilit*[ti] OR frequen*[ti] OR correlat*[ti] OR connect*[ti] OR epidemiolog*[ti] OR associat*[ti] OR relate*[ti] OR relationship[ti])))) OR "registries"[MeSH Terms]))) NOT ((((((("case report"[Title]) OR "case study"[Title]) OR "case reports"[Publication Type]) OR "review"[Publication Type]) OR "Neoplasms, Experimental"[Mesh]))))) AND English[Filter]) AND Humans[Filter] | 4735  315 | September 10, 2015  May 12, 2017^a^ |
| Embase | 'congenital disorder'/exp OR 'congenital disorder' OR 'congenital abnormalities':ab,ti OR 'congenital abnormality':ab,ti OR 'congenital defect':ab,ti OR 'congenital defects':ab,ti OR 'birth defect':ab,ti OR 'birth defects':ab,ti OR 'congenital anomaly':ab,ti OR 'congenital anomalies':ab,ti OR 'birth anomaly':ab,ti OR 'birth anomalies':ab,ti OR 'birth malformations':ab,ti OR 'congenital malformation':ab,ti OR 'congenital malformations':ab,ti AND ('neoplasm'/exp/dm_ep OR 'neoplasm'/exp OR 'neoplasm' OR 'tumor':ab,ti OR 'tumors':ab,ti OR 'tumour':ab,ti OR 'tumours':ab,ti OR 'cancer':ab,ti OR 'cancers':ab,ti OR 'neoplasia':ab,ti OR 'neoplastic':ab,ti OR 'neoplasm':ab,ti OR 'neoplasms':ab,ti OR 'carcinoma':ab,ti OR 'malignancy':ab,ti OR 'malignancies':ab,ti OR 'malignant':ab,ti) AND ('risk'/exp OR 'risk' OR 'risk assessment'/exp OR 'risk assessment' OR 'risk factor'/exp OR 'risk factor' OR 'incidence'/exp OR 'incidence' OR 'prevalence'/exp OR 'prevalence' OR 'probability'/exp OR 'probability' OR 'epidemiology'/exp OR 'epidemiology' OR risk:ti OR odds:ti OR likelihood:ti OR incidence:ti OR prevalence:ti OR 'register'/exp OR 'register') NOT ('case report':ti OR 'case study':ti OR 'case report'/exp OR 'case report' OR 'review'/exp OR 'review' OR 'experimental neoplasm'/exp OR 'experimental neoplasm') AND [humans]/lim AND [english]/lim | 9,672 | October 25, 2015 |

^a^Search run without the english language limit
