# Supplementary material for: Pediatric cancer risk in association with birth defects: A systematic review
Source: PLoS One. 2017 Jul 27;12(7):e0181246. doi: 10.1371/journal.pone.0181246 (PMC5716403; doi:10.1371/journal.pone.0181246)
Supplement: S2 Table — aCase-cohort study bNested case-control study (DOCX) [file pone.0181246.s003.docx]

| **S2 Table.**  Quality metrics for case-control, nested case-control, and case-cohort studies. | | | | | | | | | | |
| --- | --- | --- | --- | --- | --- | --- | --- | --- | --- | --- |
| **Reference** | **Selection** | | | | **Comparability** | | **Exposure** | | | **Total** |
| Adami et al., 1996 [54] | 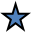 | 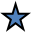 | 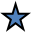 | 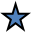 |  | 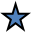 | 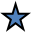 | 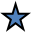 |  | 7 |
| Ager et al., 1965 [39] | 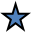 |  | 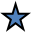 | 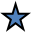 |  | 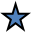 | 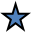 |  | 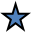 | 6 |
| Altmann et al., 1998 [17] | 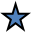 | 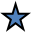 | 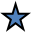 | 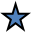 | 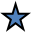 | 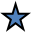 | 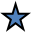 | 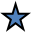 | 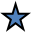 | 9 |
| Baptiste et al., 1989 [55] | 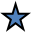 |  | 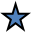 |  |  | 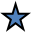 |  |  |  | 3 |
| Bailey et al., 2017[59] | 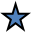 | 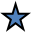 | 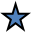 | 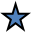 |  | 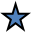 |  | 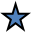 |  | 6 |
| Birch et al., 1990 [56] | 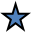 |  |  |  |  | 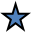 | 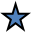 |  |  | 3 |
| Buck et al., 2001 [68] | 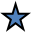 | 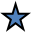 | 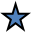 |  | 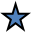 |  |  | 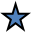 | 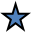 | 6 |
| Bunin et al., 1987 [78] | 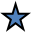 |  | 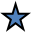 | 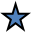 |  |  | 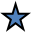 |  |  | 4 |
| Chow et al., 2007 [70] | 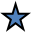 | 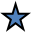 | 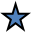 | 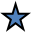 | 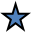 | 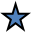 | 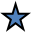 | 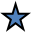 | 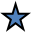 | 9 |
| Citak et al., 2011 [51] | 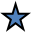 |  |  | 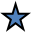 |  | 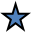 | 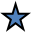 | 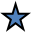 |  | 5 |
| Citak et al., 2013 [22] | 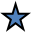 |  |  | 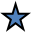 |  | 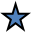 | 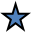 | 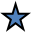 |  | 5 |
| Cnattingius et al., 1995 [49] | 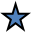 | 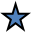 | 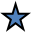 | 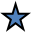 |  | 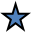 | 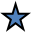 | 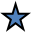 | 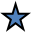 | 8 |
| Cnattingius et al., 1995 [41] | 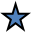 | 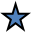 | 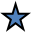 | 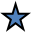 |  | 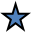 | 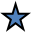 | 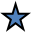 | 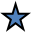 | 8 |
| Cordier et al., 1994 [61] | 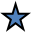 |  | 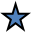 |  | 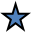 |  | 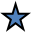 |  |  | 4 |
| Durmaz et al., 2011 [20] | 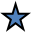 |  |  | 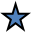 |  |  | 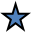 | 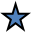 | 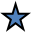 | 5 |
| Gelberg et al., 1997 [12] | 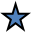 | 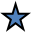 | 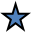 |  | 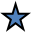 | 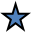 | 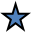 |  |  | 6 |
| Gold et al., 1994 [64] | 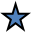 | 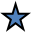 | 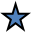 | 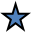 | 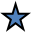 | 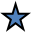 |  |  |  | 8 |
| Greenop et al., 2014[60] |  |  |  |  |  |  |  |  |  | 6 |
| Hall et al., 2017[90] |  |  |  |  |  |  |  |  |  | 7 |
| Infante-Rivard et al., 2001 [42] |  |  |  |  |  |  |  |  |  | 5 |
| Johnson et al., 1985 [66] |  |  |  |  |  |  |  |  |  | 4 |
| Johnson et al., 1987 [62] |  |  |  |  |  |  |  |  |  | 5 |
| Johnson et al., 2008 [76]^a^ |  |  |  |  |  |  |  |  |  | 8 |
| Johnson et al., 2009 [88] |  |  |  |  |  |  |  |  |  | 5 |
| Johnson et al., 2010 [46] |  |  |  |  |  |  |  |  |  | 6 |
| Kajtár et al., 1990 [79] |  |  |  |  |  |  |  |  |  | 4 |
| Lindblad et al., 1992 [81]^b^ |  |  |  |  |  |  |  |  |  | 7 |
| Linet et al., 1996 [63]^b^ |  |  |  |  |  |  |  |  |  | 8 |
| Loder et al., 2007 [25] |  |  |  |  |  |  |  |  |  | 3 |
| Magnani et al., 1990 [47] |  |  |  |  |  |  |  |  |  | 4 |
| Mallol-Mesnard et al., 2008 [58] |  |  |  |  |  |  |  |  |  | 7 |
| Mann et al., 1993 [16] |  |  |  |  |  |  |  |  |  | 5 |
| McCredie et al., 1994 [65] |  |  |  |  |  |  |  |  |  | 6 |
| Méhes et al., 1985 [19] |  |  |  |  |  |  |  |  |  | 4 |
| Méhes et al., 1998 [52] |  |  |  |  |  |  |  |  |  | 4 |
| Méhes et al., 2003 [80] |  |  |  |  |  |  |  |  |  | 4 |
| Menegaux et al., 2005 [71] |  |  |  |  |  |  |  |  |  | 4 |
| Merks et al., 2005 [26] |  |  |  |  |  |  |  |  |  | 5 |
| Merks et al., 2008 [21] |  |  |  |  |  |  |  |  |  | 6 |
| Mertens et al., 1998 [43] |  |  |  |  |  |  |  |  |  | 4 |
| Munzer et al., 2007 [69] |  |  |  |  |  |  |  |  |  | 7 |
| Neglia et al., 1988 [67] |  |  |  |  |  |  |  |  |  | 4 |
| Parodi et al., 2014 [72] |  |  |  |  |  |  |  |  |  | 8 |
| Partap et al., 2011 [57] |  |  |  |  |  |  |  |  |  | 8 |
| Podvin et al., 2006 [44] |  |  |  |  |  |  |  |  |  | 9 |
| Puumala et al., 2007 [82]^a^ |  |  |  |  |  |  |  |  |  | 8 |
| Rios et al., 2016 [74] |  |  |  |  |  |  |  |  |  | 8 |
| Roganovic et al., 2002 [50] |  |  |  |  |  |  |  |  |  | 5 |
| Rudant et al., 2013 [45] |  |  |  |  |  |  |  |  |  | 5 |
| Santos et al., 2016 [109] |  |  |  |  |  |  |  |  |  | 3 |
| Savitz et al., 1994 [15] |  |  |  |  |  |  |  |  |  | 5 |
| Schumacher et al., 1992 [23] |  |  |  |  |  |  |  |  |  | 4 |
| Shu et al., 1988 [48] |  |  |  |  |  |  |  |  |  | 6 |
| Shu et al., 1995 [89] |  |  |  |  |  |  |  |  |  | 6 |
| Spector et al., 2007 [85]^a^ |  |  |  |  |  |  |  |  |  | 8 |
| Stewart et al., 1958 [18] |  |  |  |  |  |  |  |  |  | 5 |
| Swerdlow et al., 1982 [91] |  |  |  |  |  |  |  |  |  | 5 |
| Urayama et al., 2006 [73] |  |  |  |  |  |  |  |  |  | 6 |
| Venkatramani et al., 2014 [84] |  |  |  |  |  |  |  |  |  | 8 |
| Wanderas et al., 1998 [92]^b^ |  |  |  |  |  |  |  |  |  | 8 |
| Wilkins et al., 1984 [77] |  |  |  |  |  |  |  |  |  | 6 |
| Yang et al., 1995 [86] |  |  |  |  |  |  |  |  |  | 3 |
| Zack et al., 1991 [40] |  |  |  |  |  |  |  |  |  | 8 |
| Zierhut et al., 2011 [24] |  |  |  |  |  |  |  |  |  | 5 |

^a^Case-cohort study

^b^Nested case-control study
