# Supplementary material for: Pediatric cancer risk in association with birth defects: A systematic review
Source: PLoS One. 2017 Jul 27;12(7):e0181246. doi: 10.1371/journal.pone.0181246 (PMC5716403; doi:10.1371/journal.pone.0181246)
Supplement: S3 Table — (DOCX) [file pone.0181246.s004.docx]

| **S3 Table.** Quality metrics for cohort studies. | | | | | | | | | | |
| --- | --- | --- | --- | --- | --- | --- | --- | --- | --- | --- |
| **Reference** | **Selection** | | | | **Comparability** | | **Outcome** | | | **Quality score** |
| Agha et al., 2005 [33] | 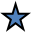 | 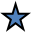 | 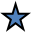 | 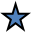 | 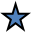 | 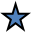 | 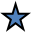 | 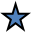 | 8 | |
| Bjørge et al., 2008 [75] | 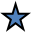 | 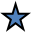 | 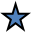 | 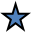 |  |  | 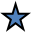 | 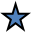 | 6 | |
| Botto et al., 2013 [32] | 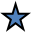 | 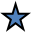 | 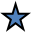 | 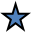 | 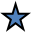 | 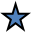 | 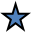 | 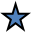 | 8 | |
| Carozza et al., 2012 [31] | 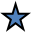 | 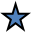 | 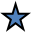 | 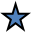 | 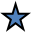 | 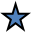 | 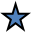 | 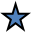 | 8 | |
| Dawson et al., 2015 [34] | 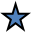 | 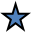 | 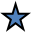 | 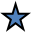 | 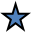 | 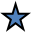 | 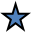 | 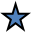 | 8 | |
| Fisher et al., 2012 [36] | 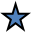 | 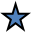 | 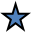 | 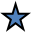 |  |  | 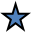 | 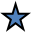 | 6 | |
| Johnson et al., 2007 [37] | 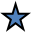 | 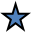 | 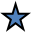 | 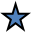 | 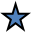 | 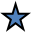 | 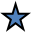 | 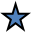 | 8 | |
| Mili et al., 1993 [28] | 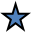 | 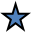 | 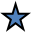 | 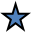 |  | 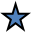 | 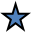 | 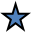 | 7 | |
| Mili et al., 1993 [29] | 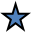 | 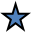 | 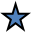 | 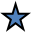 |  | 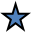 | 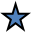 | 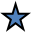 | 7 | |
| Rankin et al., 2008 [30] | 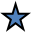 | 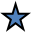 | 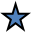 |  |  |  | 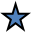 | 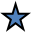 | 5 | |
| Sun et al., 2014 [38] | 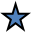 | 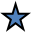 | 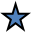 | 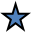 |  | 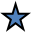 | 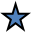 | 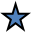 | 7 | |
| Windham et al., 1985 [27] | 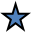 | 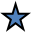 | 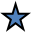 |  |  | 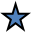 | 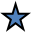 | 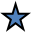 | 6 | |
| Janitz et al., 2016 [35] | 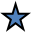 | 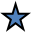 | 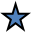 |  | 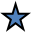 | 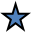 | 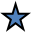 | 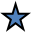 | 7 | |
